# Minimizing habitat conflicts in meeting net-zero energy targets in the western United States

**DOI:** 10.1073/pnas.2204098120

**Published:** 2023-01-19

**Authors:** Grace C. Wu, Ryan A. Jones, Emily Leslie, James H. Williams, Andrew Pascale, Erica Brand, Sophie S. Parker, Brian S. Cohen, Joseph E. Fargione, Julia Souder, Maya Batres, Mary G. Gleason, Michael H. Schindel, Charlotte K. Stanley

**Affiliations:** ^a^Environmental Studies, University of California Santa Barbara, Santa Barbara, CA 93106; ^b^Evolved Energy Research, Denver, CO 80211; ^c^Energy & Resources Group, University of California, Berkeley, CA 94720; ^d^Montara Mountain Energy, Pacifica, CA 94044; ^e^Energy Systems Management, University of San Francisco, San Francisco, CA 94117; ^f^School of Chemical Engineering, University of Queensland, Brisbane, QLD 4072, Australia; ^g^The Nature Conservancy, Sacramento, CA 95811; ^h^The Nature Conservancy, Minneapolis, MN 55415; ^i^JASenergies, San Francisco, CA 94117; ^j^The Nature Conservancy, Portland, OR 97214

**Keywords:** carbon neutrality, renewables, biodiversity, land use, siting

## Abstract

The scale and pace of energy infrastructure development required to achieve net-zero greenhouse gas (GHG) emissions are unprecedented, yet our understanding of how to minimize its potential impacts on land and ocean use and natural resources is inadequate. Using high-resolution energy and land-use modeling, we developed spatially explicit scenarios for reaching an economy-wide net-zero GHG target in the western United States by 2050. We found that among net-zero policy cases that vary the rate of transportation and building electrification and use of fossil fuels, nuclear generation, and biomass, the “High Electrification” case, which utilizes electricity generation the most efficiently, had the lowest total land and ocean area requirements (84,000 to 105,000 km^2^ vs. 88,100 to 158,000 km^2^ across all other cases). Different levels of land and ocean use protections were applied to determine their effect on siting, environmental and social impacts, and energy costs. Meeting the net-zero target with stronger land and ocean use protections did not significantly alter the share of different energy generation technologies and only increased system costs by 3%, but decreased additional interstate transmission capacity by 20%. Yet, failure to avoid development in areas with high conservation value is likely to result in substantial habitat loss.

## Significance

Reaching net-zero greenhouse gas emissions requires a major build-out of low-carbon infrastructure—including wind and solar farms and transmission lines—that potentially require significant land and ocean area. We studied the land and ocean use implications of a national net-zero target in the western United States. We identify multiple options for limiting the land/ocean use impacts of reaching net zero, including energy technology choices and protecting high conservation value areas. The scenario with the highest level of protection and that most rapidly and extensively electrifies buildings, cars, and industry has the lowest overall impacts. We found that strong land/ocean use protections incur a small (∼3%) energy cost premium while greatly reducing siting conflicts that would likely delay renewable energy deployment.

A growing number of countries and subnational governments have adopted policies that aim to reach net-zero greenhouse gas emissions by mid-century in order to avoid serious consequences of climate change (1). A number of states in the United States have adopted an economy-wide target of net-zero emissions by mid-century, while more than a dozen have 100% clean electricity goals (2). Achieving any of these goals hinges on the rapid decarbonization of the energy system, with electric sector decarbonization leading the way (3, 4). The most recent roadmaps for reaching net zero in the United States find that the projected pace of wind and solar power plant development frequently reaches 3 to 4 times the current build rate with total wind and solar capacity reaching 10 to 30 times and biomass use reaching 2 to 5 times their current levels by mid-century (5).

The area of land and ocean that would be required over the next 30 y by this level of low-carbon energy infrastructure expansion is large (5). The potential ecological and social impacts of this development on landscapes, seascapes, and communities all across the United States could be significant, depending on the type, scale, and location of the power plants, transmission lines, and bioenergy resources involved (6). The unintended consequences of large-scale solar and wind development (7, 8) have already spurred “green vs. green” conflicts (9) between clean energy advocates and conservationists over the siting of renewable energy facilities in sensitive landscapes or seascapes (10).

Previous studies have found that environmental and/or social siting constraints on the development of wind and solar power have the potential to significantly affect costs and optimal technology mix (6, 11, 12). Recent advances in power sector modeling have improved the representation of siting constraints and the spatial specificity of model outputs (12). However, most prior studies focused on energy, and siting constraints have examined a limited geographic area, one or two generation technologies in isolation, no spatially explicit treatment of transmission infrastructure needs, and/or the requirements of a clean electricity target only, rather than an economy-wide net-zero target (6, 12, 13). The Net Zero America study (14) developed economy-wide net-zero pathways with a full suite of technologies, along with illustrative maps of infrastructure development but did not examine the potential habitat loss of siting this infrastructure or the effects that avoiding infrastructure development in ecologically and culturally sensitive locations could have on energy system technologies and costs.

In this study, we examine the potential habitat loss and land/ocean use impacts of developing the complete set of onshore wind, offshore wind, large-scale solar, distributed solar, transmission, and bioenergy resources needed to reach economy-wide net-zero greenhouse gas (GHG) emissions by 2050 for the 11 western US states (i.e., Arizona, California, Colorado, Idaho, Montana, Nevada, New Mexico, Oregon, Utah, Washington, and Wyoming). The modeled scenarios achieve economy-wide net-zero emissions for the whole United States by 2050, but we explore the specific implications of achieving this target in the western United States by representing the energy system, including supply, demand, and transmission infrastructure, and their land and ocean use impacts, in much greater detail and realism for the western region (*SI Appendix*, *Energy Modeling Methods*). We also examine how much and where this infrastructure might be located in order to best protect ecosystems and avoid potential siting conflicts that could undermine decarbonization goals. We combined high-resolution energy and geospatial modeling with detailed land use and conservation data to develop spatially explicit representations of different net-zero energy system portfolios using a variety of assumptions about energy policy and environmental (i.e., land/ocean use) protections. This study advances prior work along several key dimensions: a) sectoral scope (economy-wide); b) emissions target (net zero); c) technology coverage (all major technologies, including offshore wind, biomass, and direct air capture); d) transmission infrastructure (realistic, spatially explicit modeling of inter and intra-state power lines and spur-lines); e) geographic scope (region-wide emissions target); f) land/ocean use siting criteria (highly detailed, state-specific datasets); and g) social metrics (multiple socioeconomic indicators).

The objective of this study was to identify ways to minimize the negative land and ocean use impacts of renewable energy development needed to achieve net-zero emissions. To achieve this objective analytically, we asked the following questions: 1) What are the land and ocean area requirements across a range of net-zero and siting scenarios? 2) How does protecting land and ocean areas with high conservation value affect the cost and technology choices of reaching net zero? 3) What are the ecological and landscape impacts of protecting areas with high conservation value and how do the demographics of host communities vary across scenarios?

To answer these questions, we first develop and apply siting constraints based on detailed environmental and land-use data to identify the locations available for energy development. Using the resulting candidate project areas, we model the routes and costs of power lines using techno-economic and environmental (i.e., land/ocean use) criteria. We then provide these as inputs to RIO, a high-resolution capacity expansion model of the energy supply system, which determines the cost-optimal energy portfolio (5) for each scenario. We spatially downscaled the onshore wind, offshore wind, solar, and transmission requirements for each portfolio using an empirical approach that relies on historical siting trends. Finally, to perform a “strategic environmental assessment,” we quantified the area of agricultural lands, natural lands, ecologically sensitive lands (e.g., critical habitat for certain focal species) and intact landscapes affected by development in each scenario. We did not assess air, water, pollution, soil, or other highly local environmental impacts. For social impacts, we evaluated the average income, percent living below poverty, percent unemployed, and total population living within a certain vicinity of an infrastructure project for each scenario.

We examined a wide range of scenarios, combining three energy cases that reach net-zero economy-wide with three levels of environmental protection. The main energy cases include a high rate of electrification (High Electrification), a slow rate of electrification (Slow Electrification), and no fossil fuel or additional nuclear power by 2050 (Renewables Only) (Table 1). Sensitivities that modified other parameters including the build rate for wind and solar, the extent of regional energy trade, and the extent of biomass use are reported in (*SI Appendix*, Table S10). Siting Levels 1, 2, and 3 (SL1, SL2, and SL3, respectively) represent increasing levels of land and ocean use protection from the development of onshore wind, offshore wind, large-scale solar, power lines, and biomass (*SI Appendix*, Tables S10, S17–S20 and Figs. S1 and S2). In recognition of Tribal sovereignty, this analysis did not include renewable resources within Tribal lands as candidates for development (15). For comparison purposes, we also modeled a reference scenario with no carbon constraints and no land/ocean use protections that emits 1008 MT(C)/y in 2050 (*SI Appendix*, Fig. S15) and an Electricity Only case that achieves net zero in the power sector only (Table 1).

**Table 1.** Description of energy cases and environmental siting levels

	Name	Description
ENERGY Economy-wide cases	High electrification	Demand: High energy efficiency, 100% sales of electric building technologies by 2040, 100% ZEV sales by 2040, fuel switching for some process heat and other fuel use, direct reduced iron-making (DRI), which uses hydrogen and electricity instead of coal, in iron and steel, carbon capture on cement (5). Supply: All generation technologies allowed; Carbon target: Net-zero economy-wide emissions by 2050
	Renewables only	Demand: Same as high electrification. Supply: No fossil fuel usage or nuclear generation by 2050. Carbon target: Net-zero economy-wide emissions by 2050
	Slow electrification	Demand: High energy efficiency, 100% sales of electric building technologies by 2060, 100% ZEV sales by 2060, 20-y delay in fuel switching for process heat, other fuel use, and DRI in iron and steel, carbon capture on cement. Supply: All generation technologies allowed. Carbon target: Net-zero economy-wide emissions by 2050
ENERGY Comparison cases	Electricity only	Demand: Reference case electricity demand (demand for fuels is outside of system boundaries). Supply: Electricity system only. Carbon target: Net-zero emissions constraint in only the electricity sector by 2050, which by itself does not meet an economy-wide net-zero goal but reflects the vast majority of the most ambitious targets adopted by Western states
	Reference	Demand: Existing energy efficiency, low electrification of buildings, 10% EV adoption, no industry electrification. Supply: All generation technologies allowed. Carbon target: None
ENVIRONMENTAL Siting levels	Siting level 1 (SL1)	Wind and solar: Exclude legally protected areas (Category 1, eg., national parks, wilderness areas, wildlife refuges, conservation easements). Biomass: all feedstocks included; exclude potential supply from conservation reserve program land
	Siting level 2 (SL2)	Wind and solar: Exclude administratively protected areas (Category 2; e.g., critical habitat for threatened and endangered species, wetlands, areas of critical environmental concern) and Category 1. Biomass: No net expansion of land for purpose-grown herbaceous biomass crops. Land for purpose-grown biomass is restricted to land that is currently used to grow bioenergy feedstocks. Specifically, land available for herbaceous biomass crops (miscanthus and switchgrass) is limited to the share of land currently cultivated for corn that is eventually consumed as corn ethanol, which is phased out in all net-zero scenarios by 2050 (14)
	Siting level 3 (SL3)	Wind and solar: Exclude areas with high conservation value (Category 3; e.g., priority and crucial habitat, intact grasslands, prime farmlands), category 2 and category 3. Biomass: Same as siting level 2

## Results

### Effect of Energy System Choices and Siting Constraints on Land and Ocean Area Requirements.

**Fig. 1.**
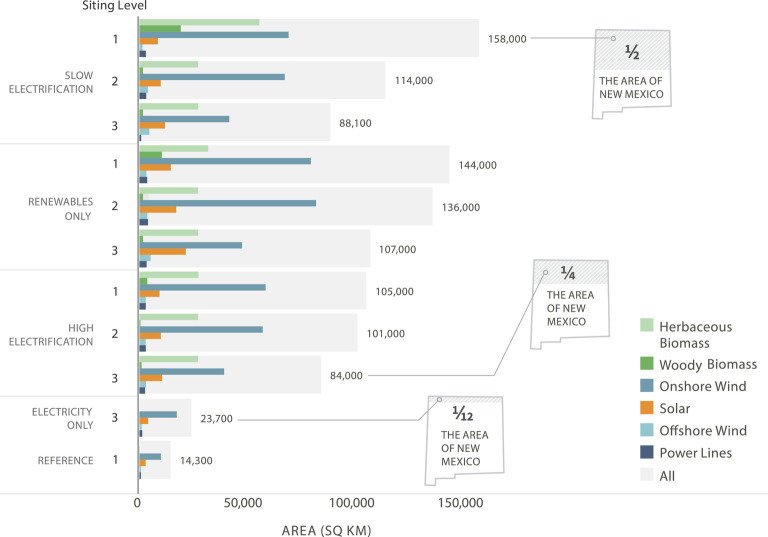
Land and ocean area for renewable resource and additional biomass resources required in 2050 for each scenario. Gray bars indicate the total area summed across the grouped bars for each scenario. Onshore and offshore wind plant area shown represents the total area, which includes spacing between turbines. The land requirements for hydropower, natural gas, coal, and nuclear are not included because their capacities either remained constant or declined over time. Direct air capture (DAC) and geothermal plants are also not included because their land requirements were minuscule compared to wind, solar, transmission, and biomass. However, all renewable generation used to power DAC is reflected in the area requirements (17, 18).

**Fig. 2.**
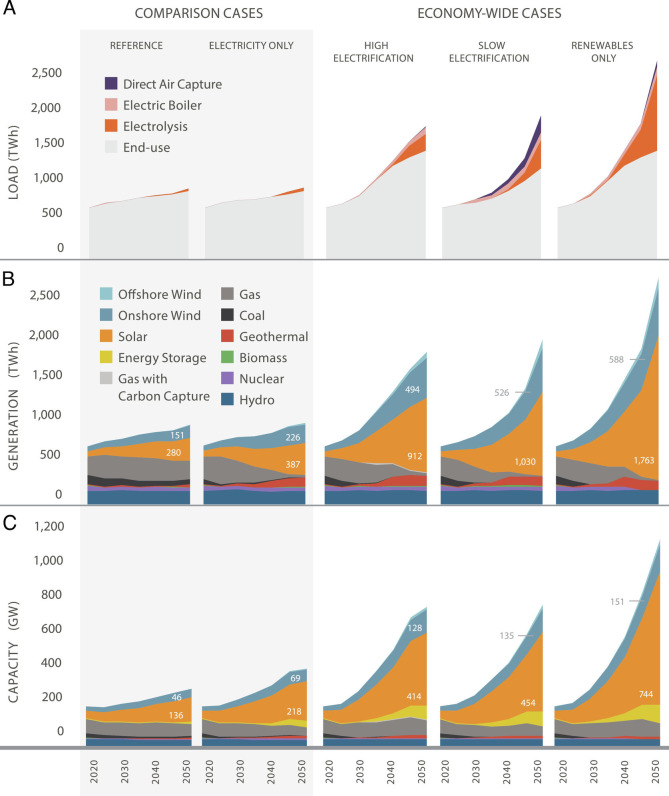
Electricity demand (Load) (*A*), generation (*B*), and installed capacity (*C*) across the western United States for scenarios that achieve different decarbonization targets (reference, electricity only, and economy-wide net-zero cases) or with different decarbonization pathways (High Electrification, Slow Electrification, and Renewables Only). The reference case uses Siting Level 1 (SL1) protections, while all other scenarios have Siting Level 3 (SL3) protections. Values for onshore wind and all solar generation and capacity (large-scale, urban infill, and rooftop) are labeled.

Wind, solar, and biomass—the lowest-cost forms of carbon-neutral primary energy in a net-zero system—are land-intensive (16). As a result, the area of land and ocean required to supply primary energy in a renewables-based system is sizable (Fig. 1). The need to decarbonize electricity generation while simultaneously electrifying transportation, buildings, and industry drove the growth in renewable and transmission capacity requirements. Our results show that by 2050, in comparison to the reference case system that was not carbon constrained, the total electricity-generating capacity requirement of a high-renewables, net-zero energy system was 3 to 4.5 times greater, transmission capacity was 47% to 65% greater, and biomass consumption was up to 2.5 times greater (Fig. 2, *SI Appendix*, Table S21). These capacity increases resulted in land and ocean area requirements 5 to 11 times greater than in the reference case (Fig. 1).

The unprecedented scale and pace of this energy development has major land and ocean use implications. By 2050, onshore wind, offshore wind, large-scale solar, transmission lines, spur-lines, and purpose-grown herbaceous and woody biomass resources constituted most of the additional area requirements for energy supply in the western United States (Fig. 1). The total area requirements for these technologies ranged from 55,000 to 158,000 km^2^ across the economy-wide net-zero scenarios or roughly one-fourth to one-half the area of the state of New Mexico (Fig. 1).

The net-zero scenario with the lowest total land use requirement is High Electrification under Siting Level 3, which protects lands and waters with high conservation value (Fig. 1). Across all levels of environmental protection, the High Electrification case was consistently less land intensive than the Slow Electrification and Renewables Only cases. This is largely because the Slow Electrification case, which reduces the rate of electrification, actually resulted in slightly higher renewable capacity and generation requirements by 2050 in order to meet higher demand for biogenic carbon used in electrically produced fuels arising from slower transportation electrification (Fig. 2 and *SI Appendix*, Table S21). The Renewables Only case, which completely eliminated nuclear generation and fossil fuel use, was able to avoid the additional energy associated with carbon capture and geological sequestration. Nonetheless, it had the highest renewable capacity requirements in part because the total elimination of fossil fuels meant more synthetic renewable fuel production (Fig. 2 and *SI Appendix*, Table S21). Since area requirements scale with generation capacity and biomass demand, net-zero scenarios that use more energy have greater land and ocean area needs. For a more thorough explanation and discussion of energy portfolio dynamics and results, *SI Appendix*, *pg. 35*.

The reduction in the relative area requirements for High Electrification compared to other economy-wide cases was largest under the least protective siting constraints, SL1 (33% relative to Slow Electrification and 27% relative to Renewables Only) but was less under the most protective Siting Level 3, particularly for Slow Electrification (only a 5% reduction). Two factors explain this. First, there is high demand for and unconstrained availability of herbaceous and woody biomass under SL1. Second, the area occupied by onshore wind, which had the largest area requirement of all technologies, was significantly lower in SL3, while higher-density solar PV and offshore wind projects increase. Note that wind area requirements include spacing between turbines as this better represents the extent of possible avian impacts and siting challenges.

Direct electricity supply and use are matched within the 11 western states, but both fossil and renewable (e.g., biogenic) fuels are allowed to be imported from and exported to other states, as is the current norm with petroleum and natural gas. As such, land and ocean use requirements reported in Fig. 1 may include those outside of the western United States.

### Effects of Siting Constraints on a Net-Zero Energy System.

We found that avoiding development in high conservation-value locations when siting renewable energy and transmission, along with limiting land requirements for biomass, had relatively small impacts on energy supply portfolios and modest impacts on costs. They did not significantly alter the main types of energy resources used—solar and wind generation remained the dominant, lowest-cost primary energy supply in all Siting Levels.

#### Onshore wind vs. solar.

**Fig. 3.**
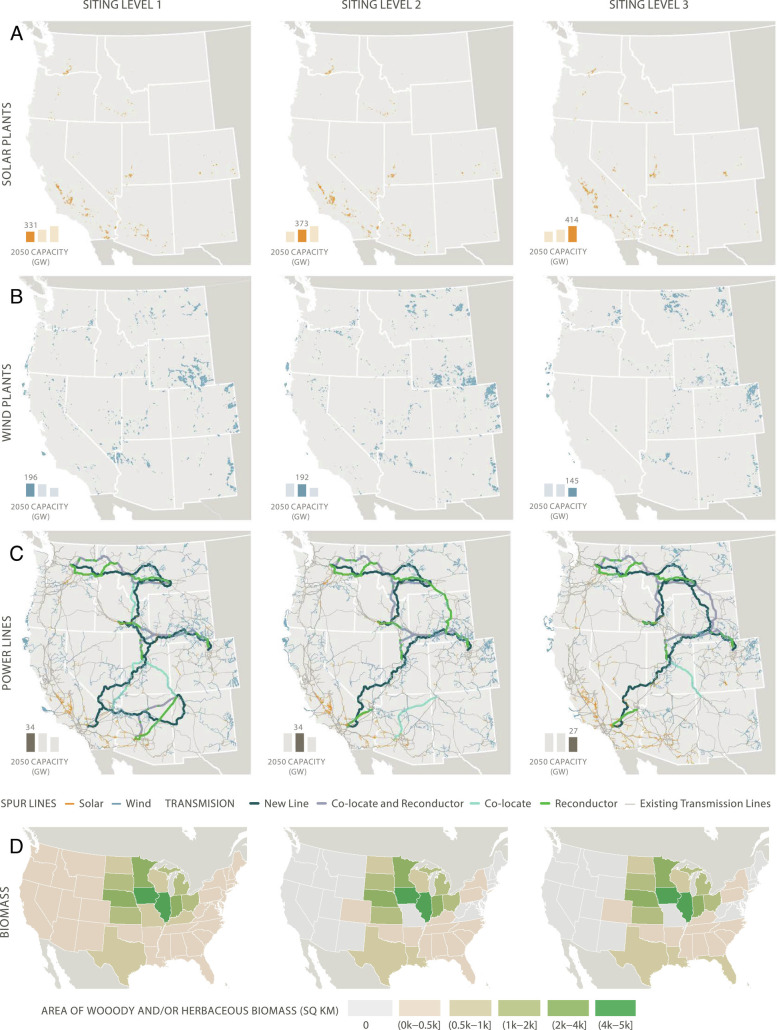
Additional build-out by 2050 of large-scale solar plants (*A*), wind plants (*B*), transmission and spur lines (*C*), and the western United States share of purpose-grown biomass area requirements (*D*) under different levels of environmental protection and supply constraints for the High Electrification case (Siting Level 1 in the left column through Siting Level 3 in the right column). The total capacity for each infrastructure type in gigawatts (GW) is indicated by the bars in the lower left corner of each map. Total solar capacity bars include large-scale, urban infill, and rooftop solar, whereas the map shows the location of only additional large-scale solar plants. Biomass maps indicate the area in each state required to supply the western US states’ share of purpose-grown biomass. Siting Levels 2 and 3 restrict purpose-grown herbaceous biomass cultivation to land dedicated to growing corn for corn ethanol. These modifications preclude downscaling biomass demand beyond the state level.

Increasing ecosystem protections shifted resource development from onshore wind to solar PV, often with battery storage (Fig. 3 and *SI Appendix*, Table S21). Onshore wind generation was the most sensitive to increasing levels of land-use protections mainly because there are fewer areas economically suitable for wind development, and wind has much lower total land use efficiency than solar (6). In the High Electrification case, as siting constraints increased from SL1 to SL3, wind generation decreased by 26% and solar increased by 25%. The differences were greatest in the scenarios that required the highest levels of renewable generation (e.g., wind decreased 37% and solar increased 52% in the Renewables Only case; *SI Appendix*, Table S21). The differences were more pronounced—and in some cases even moved opposite to the overall regional trend—for some individual states, as a function of changing relative costs among states when some resources were eliminated (Fig. 3). While total onshore wind capacity decreased from 180 GW to 129 GW in the High Electrification case with more protections, it nonetheless increased in Montana, while declining precipitously in Wyoming, Nevada, and New Mexico (Fig. 3). The specific locations of future power plants and their associated impacts may vary significantly as a result of protections (Fig. 3 and *SI Appendix*, Fig. S23).

#### Transmission.

Increased ecosystem protections reduced additional transmission capacity due to the redistribution of new energy infrastructure away from the interior and toward the coast and closer to the majority of customer load (Fig. 3). New transmission capacity was 20% lower in SL3 relative to SL1 (Fig. 3 and *SI Appendix*, Fig. S22 and Table S21). Nonetheless, new transmission and upgrades were required in all net-zero scenarios. In the core cases, transmission capacity would need to increase 30% to 70% above today’s level (*SI Appendix*, Fig. S22). New transmission lines predominantly high-voltage direct current (HVDC), lines comprised nearly half of the interstate capacity additions by 2050 across most scenarios. Importantly, the remaining half consisted of colocated high-voltage alternating current (HVAC), involving additional transmission towers along existing rights-of-way, and reconductored lines, which upgrades conductor wires from 230 kV to 500 kV on existing transmission towers (approximately 40% and 10%, respectively, or 15 GW total, in High Electrification SL3; *SI Appendix*, Fig. S22)—both significantly reduce land disturbance.

#### Distributed solar.

Large land requirements were reduced, but not eliminated, by increasing rooftop PV and urban infill PV. For example, the 82-GW increase in solar capacity resulting from maximizing siting protections (SL3) in the High Electrification scenarios could be fully met by distributed solar if it could reach the relatively ambitious level of 35% of its technical potential (80.5 GW for new rooftop and 73.5 GW for new urban infill). However, distributed solar alone is insufficient to meet economy-wide solar needs, which reached as high as 414 GW in the High Electrification SL3 scenario (*SI Appendix*, Table S21).

#### Offshore wind.

While distributed and large-scale solar PV technologies are nearly interchangeable after adjusting for efficiencies, wind and solar resources tend to be more complementary than interchangeable (5, 6, 19) and wind has a high value in a solar-dominated power system. Despite this, across scenarios, offshore wind capacity was a relatively small part (7 to 27 GW) of the capacity mix (Fig. 2 and *SI Appendix*, Table S21). Compared to the East coast, where studies have found offshore wind to play a major role in any low-carbon generation mix (20), the West coast has higher costs due to greater ocean depth, longer transmission distances, and the relative abundance of competing onshore resources. The largest build-out of offshore wind capacity (27 GW) was in the Renewables Only cases under SL3, in which onshore wind availability was insufficient (*SI Appendix*, Table S21). Environmental siting exclusions did not appear to limit the selection of offshore wind in any scenario.

#### Biomass.

Biomass is the most land-intensive form of energy production (16, 21). Even in the absence of land-use constraints (SL1), biomass demand was limited by its challenging economics. In the High Electrification case, biomass requirements in the western states were nearly identical across Siting Levels 3.2 exajoules (EJ) in SL1 and 3.3 EJ in SL2; *SI Appendix*, Table S21 and Fig. S20, though where the purpose-grown biomass would be sourced changes (Fig. 1*D*). Limits to biomass availability had a larger impact in the Slow Electrification and Renewable Only scenarios, with Slow Electrification SL1 having the highest biomass consumption (5 EJ), declining to 3.2 EJ in SL3 (*SI Appendix*, Table S21). Since there are no limits on interstate transport or trade of biomass for synthetic fuel production, a significant fraction of purpose-grown—nonwaste and nonresidue—biomass (primarily miscanthus and switchgrass) would be sourced from midwestern states responsible for growing most of the corn for corn ethanol and southern states where woody biomass is most cost-effectively produced (Fig. 1*D* and Table 1). In a sensitivity analysis in which we further limited biomass supply to only wastes and residues (Limited Biomass case), there was a substantial reduction in biomass consumption (from 3.3 to 2.0 EJ). This reduction was in part compensated by greater wind and solar generation used for synthetic fuel production, but also by higher fossil fuel use in transportation offset by higher DAC (45 Mt/y) (*SI Appendix*, Table S21 and Figs. S17 and S20).

#### System costs.

**Fig. 4.**
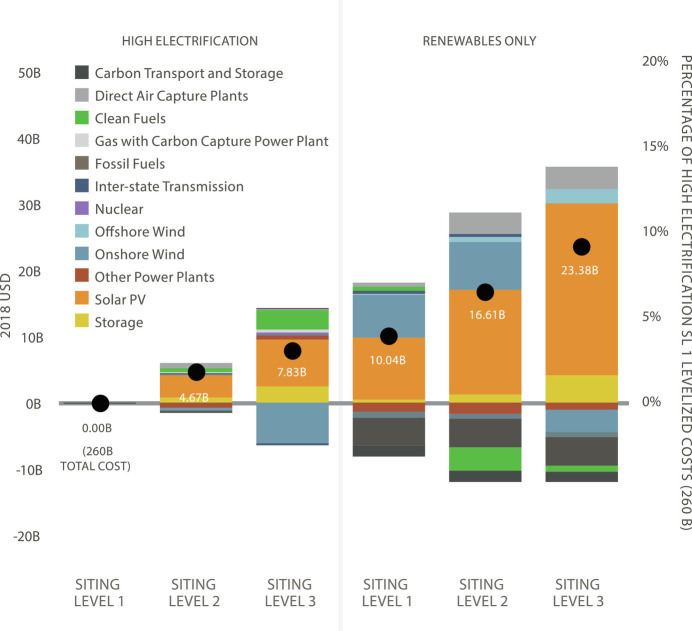
Net annual levelized supply-side cost in 2050 for scenarios is shown relative to the High Electrification Siting Level 1 scenario. Bars above the x-axis represent an increase in costs, while bars below represent a decrease in relative cost. Labeled points provide the net cost. The secondary y-axis shows the percentage cost increases compared with the High Electrification SL1 scenario, which costs 260 billion USD.

The relatively limited effects of siting constraints on the technology mix also extended to energy costs, with SL3 protections resulting in only a modest increase in net costs (*SI Appendix*, Table S21 and Fig. 4). Higher spending on solar, storage, and clean fuels under more stringent land use protections were partially offset by savings from lower installed onshore wind and transmission capacity. In the High Electrification case, the additional energy system cost for achieving the highest level of protection (SL3) was $7.8B USD per year by 2050, a 3% increase over SL1. Costs increased further in order to achieve additional policy objectives (e.g., 14% for Renewables Only) or due to additional constraints on resource availability explored in sensitivity analyses (e.g., 3.8% for Limited Biomass; *SI Appendix*, Table S21 and Fig. 4).

### Environmental and Social Impacts of Renewable Energy Development.

#### Ecological impacts.

**Fig. 5.**
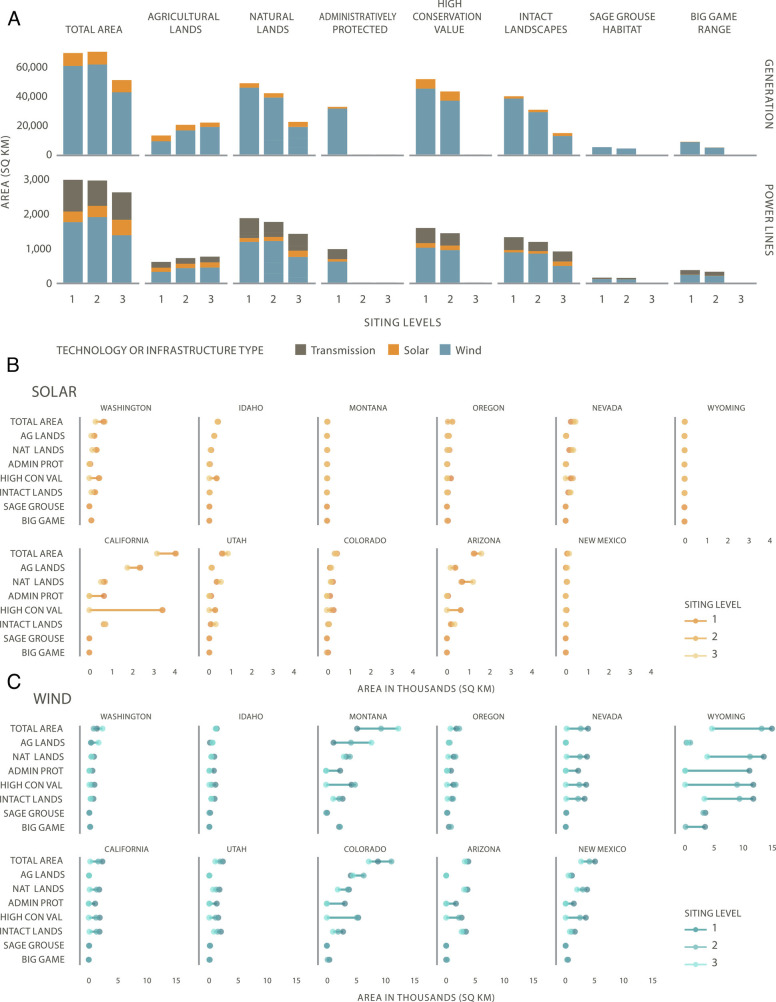
Land use, land cover, and select ecological impacts of solar and onshore wind project areas (generation) and transmission lines selected for each Siting Level under the High Electrification case (*A*). Impacts of selected solar and onshore wind project areas for the same metrics are reported by state (*B* and *C*). The length of the lines connecting points for each metric indicates the difference between Siting Levels or the degree of impact of land use protections. Natural lands comprise grasslands, shrubland, and conifer land cover classes.

We found that land and ocean use protections avoided renewable energy development on lands and waters with high conservation value, while still providing more than adequate wind and solar energy to meet net-zero targets. Without such protections, between 60% and 70% (up to 42,000 km^2^) of onshore wind and solar development occurred in High Conservation Value areas in the High Electrification case (Fig. 5*A*). Of the ecological categories examined, intact landscapes and wildlife corridors benefited the most from siting protections for onshore wind; in SL3 scenarios, development was reduced by 60 to 70% in these areas (Fig. 5*A* and *SI Appendix*, Fig. S27). Despite relatively large landscape-level impacts across all Siting Levels, the impacts on habitats of sensitive and significant focal taxa such as Sage Grouse and big game were relatively small, with energy development affecting less than 3% of total Sage Grouse habitat in SL1 and SL2 (Fig. 5*A*); however, impacts to Sage Grouse habitat in individual states were more significant (Fig. 5*C*).

#### Land use impacts.

A significant share of new solar, wind, and transmission development occurred on agricultural lands across all policy scenarios (Fig. 5*A* and *SI Appendix*, Fig. S27). Across all core cases, increasing ecosystem protections doubled the total share of wind and solar development on agricultural lands, while more than halving the capacity sited on natural lands (shrublands, forest, and grasslands) (Fig. 5*A* and *SI Appendix*, Fig. S27). For solar, 40 to 50% of capacity was sited on agricultural lands across siting levels, but the types of agricultural land changed (Fig. 5*A*). With only SL1 or SL2 protections, a large share of the solar was sited on prime farmland (80 to 90%; *SI Appendix*, Fig. S24), but when prime farmland was excluded from development in SL3, solar development shifted to other, nonprime agricultural lands. For onshore wind, agricultural land’s share increased from 15 to 30% of total onshore wind area as more siting protections were applied. A similar shift from natural to agricultural lands was observed for transmission (Fig. 5*A*).

Impacts were not uniformly distributed across the western states. For example, in the High Electrification case, under SL1 and SL2, nearly 90% of the total solar power plant area in California occurred on High Conservation Value lands, compared to 50% in Arizona (Fig. 5*B*). For onshore wind, the vast majority of Sage Grouse habitat and big game range impacts were concentrated in Montana and Wyoming, with Sage Grouse habitat areas accounting for about 20% of all wind development in Wyoming under SL1 and SL2 (Fig. 5*C*). For transmission, the largest build-outs and associated impacts occurred in Washington, Utah, and Idaho (Fig. 3). Impacts also do not follow a linear trend across Siting Levels for some states. For example, the amount of solar development on natural lands in Arizona increases under SL3 because of a reduction in solar power plants located on agricultural lands in scenarios that protect prime farmland (Fig. 5*B*).

#### Potential social implications.

Siting constraints can alter which communities host large-scale renewable energy infrastructure and receive the associated benefits and impacts. The presence of renewable energy in a community is typically viewed favorably by some members of the community (due to the economic and climate benefits) and unfavorably by other members of the community due to cultural, environmental, aesthetic, noise, or other concerns (22). We evaluated each scenario using a suite of social vulnerability metrics. We found that changing Siting Levels had minor effects on average income, percent living below the poverty line, percent unemployed, and population density of communities hosting wind and solar projects (*SI Appendix*, Fig. S28). However, these social metrics did differ between technologies (*SI Appendix*, Fig. S28). Wind projects tended to be sited in more affluent and less densely populated areas compared to solar projects (*SI Appendix*, Fig. S28). This is likely a result of solar projects being located relatively closer to loads and substations in more urban areas, which are not only denser, but also have a greater range of socioeconomic statuses.

**Fig. 6.**
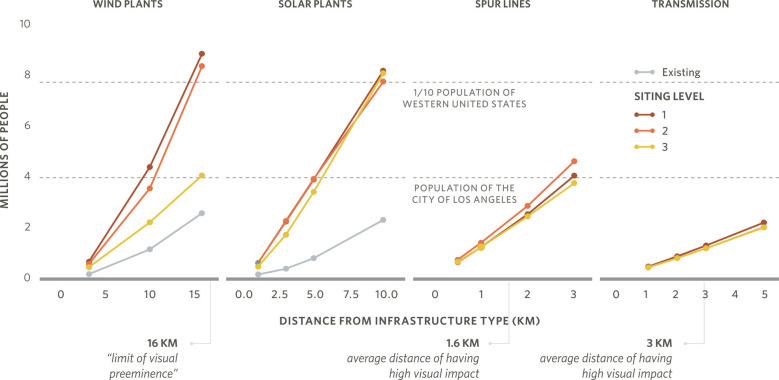
Human population found within a certain distance from all selected wind plants, solar plants, spur lines, and transmission lines for each Siting Level in the High Electrification case. Population counts were estimated for the buffered distances indicated by the points. Visual impact studies have identified 16 km as the limit of visual preeminence for wind plants, 1.6 km for 230-kV lines, and 2-3 km for 500-kV transmission towers. The population of the city of Los Angeles and a fraction of the population of the western United States are provided for reference.

We evaluated the changes in population density and population count at various distances from selected wind and solar farms as a function of siting constraints. Though counterintuitive, we found that the population density at any given distance within 16 km from new wind or solar energy infrastructure generally decreased with increasing ecosystem and land use protections (*SI Appendix*, Fig. S29). This may be due to shifting more development into rural areas, which have lower population density. This, combined with lower wind capacity, resulted in fewer people being in close proximity to renewable infrastructure (Fig. 6). In total, 9.5 to 12.5 million people in the western United States (roughly 12 to 15% of the current western population) could be living within 16 km of a wind plant, 3 km of a solar plant, 3 km of a new transmission line or expanded/upgraded corridor, or 1.6 km of a spur line (Fig. 6), distances within which high visual impacts have been reported (23–26).

## Discussion and Conclusions

We find that the scale, pace, and land use requirements of the energy infrastructure build-out required to achieve net-zero economy-wide emissions are unprecedented. Yet, if this transition is adequately planned, it is technically feasible, affordable, and environmentally sustainable. The energy policies adopted to meet net-zero goals can strongly influence, directly or indirectly, where generation, transmission, and bioenergy production will be located and how much land and ocean area will be required for it, by virtue of how they shape electricity and fuel supply, demand, and delivery. We find a factor of three difference between the most and least area-intensive net-zero scenarios. To avoid unintended environmental consequences, it is important to consider how the adoption of different energy technologies impacts overall land and ocean use.

### Energy Pathway Decisions Will Strongly Affect Renewable and Transmission Land Use.

Policies that lead to rapidly electrifying vehicles, buildings, and industry result in more efficient use of primary energy resources, reducing the capacity requirements for solar and wind, the demand for biofuels, and consequently the area of land/ocean required. On the other hand, policies that eliminate all fossil fuel use by 2050 are technically feasible, but the last increment of fossil fuels to be eliminated comes at a high cost in increased demand for electricity, biomass, and land. Policies that retain, rather than retire, gas thermal generating capacity, allowing it to be available for infrequent use when needed for reliability, will help to reduce costs, land requirements for infrastructure siting, and the need for biomass.

The scale of bioenergy use differed widely across policy cases (*SI Appendix*, Table S21), generally increasing when other energy supplies are limited. At the same time, we found that net-zero scenarios are feasible without expanding the land area used for purpose-grown biomass. In the Limited Biomass sensitivity, energy crops’ land area shrinks relative to today. While this avoids about 22,000 km^2^ of land use, by 2050, the Limited Biomass sensitivity uses 11% of the petroleum required in the reference scenario compared to only 4% of the reference scenario for High Electrification SL3. Limited Biomass is also associated with a 4% point increase in costs compared to the High Electrification case. While there are no precedents for policy levers or market incentives to achieve biomass-limited outcomes in the United States, results indicate that the availability of sustainable sources of biomass beyond wastes and residues may prove important for limiting fossil fuel use and additional costs.

### Siting Constraints Effectively Protect Areas with High Conservation Value at Low Cost, While Reducing the Number of People Impacted by Development.

We found that without new siting constraints, a net-zero energy system could significantly impact areas with high conservation value (e.g., intact lands, wildlife corridors). Conversely, we found that these impacts can be dramatically reduced through land/ocean use protections and restricting cultivation of purpose-grown biomass to areas currently under bioenergy crop production. Ecosystem protections can be achieved at a relatively low cost premium, about a 3% increase in energy system net cost in 2050 (i.e., when increasing protections from SL1 to SL3). Even 3% may be an overestimate because it does not reflect the possible higher mitigation (27, 28) or permitting costs of projects in areas with higher ecological value, which are also more likely to be canceled (29). We found that increasing siting protections actually reduces transmission requirements. With greater granularity and realism in representing the transmission system compared to previous studies (13, 30), we found about half of new transmission capacity can be met by upgrading existing lines or expanding existing right-of-ways, which significantly limits land impacts and siting hurdles.

The support of farmers and rural communities will be essential for protecting habitat because implementing more ecosystem protections shifts development from natural to agricultural lands. This comes with its own challenges, but we have shown that it is possible to avoid the use of prime farmland. Additionally, agrivoltaics (siting solar panels alongside crops) can increase the land use efficiency per unit area of both solar energy and food production (31, 32). Solar development on marginal or fallowed lands can provide farmers with alternative income sources (33). Involving stakeholders early in energy planning and siting processes can help ensure that communities benefit from projects colocated on working lands or within fishing areas (10, 34).

Siting choices involve many real-world social tradeoffs. On the one hand, new infrastructure can create some local economic opportunities (e.g., tax revenue). On the other, it also alters the viewshed, potentially precludes other beneficial land uses, and could reduce property values (35). The acceptable balance of benefits and costs for any project will be largely determined by local stakeholders. We found that roughly 12% of the current population in the western states may be affected at least visually by a net-zero build-out by midcentury assuming High Electrification—and more in other cases. While Tribal lands were not included in the renewable resource assessment out of respect for Tribal sovereignty, energy development may occur in neighboring lands and waters, with possible viewshed, socioeconomic, or cultural impacts. It is critical to include Tribal participation early in low-carbon transition planning to ensure community benefits and the protection of cultural landscapes.

### Conclusion.

Given the doubling and tripling of build rates for low-carbon infrastructure required by mid-century, current capabilities in policy, planning, and stakeholder engagement in support of environmentally and socially responsible infrastructure siting may be insufficient. A sense of urgency stems from the need for a rapid ramp-up in the rate of solar and wind development, in contrast with a well-documented reduction in the capacity and increase in the costs to build large infrastructure projects in the United States (36).

Generating spatially explicit scenarios, like those demonstrated in this study, can help minimize siting conflicts in order to meet climate targets. A key feature of the approach is high spatial detail, which enables local stakeholders, land use planners, and conservation organizations to concretely envision and participate effectively in the planning process. It recognizes that successfully building a net-zero energy system rests on place-based decisions. While some region-wide assumptions made in this study limit its direct application in planning and policy (e.g., states are likely to adopt different environmental permitting standards), our spatially explicit planning framework is flexible enough to be implemented at multiple jurisdictional scales and should be replicated in inclusive stakeholder processes that ground assumptions in local realities.

## Materials and Methods

### Methods Overview.

**Fig. 7.**
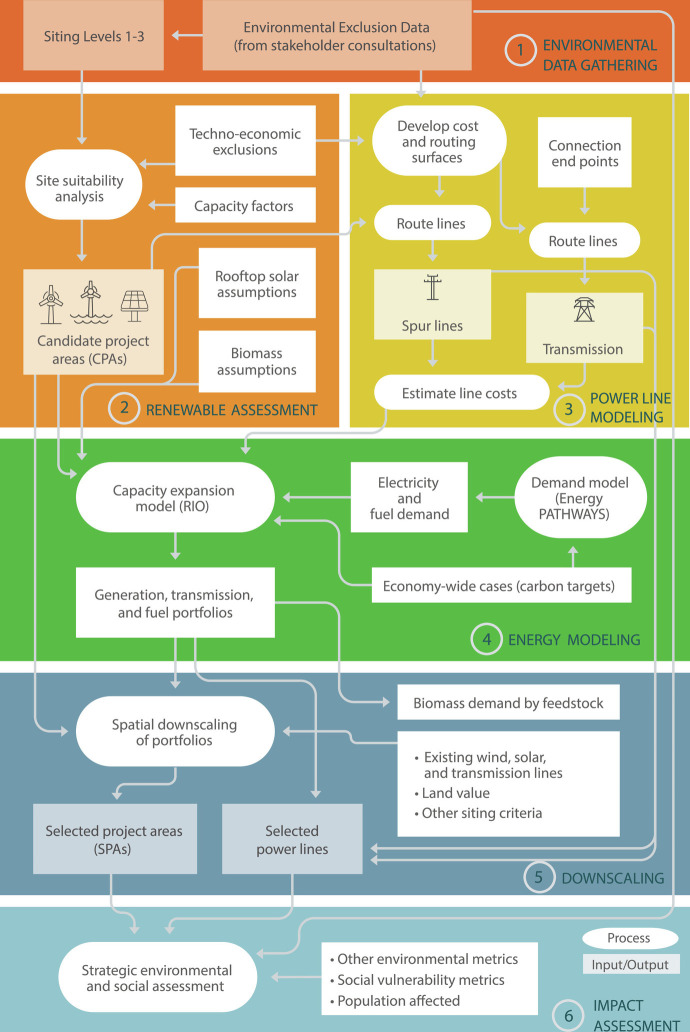
Methodological framework.

The methodology contains six key stages, summarized in Fig. 7 and described briefly below. Please refer to *SI Appendix* for more detailed descriptions, figures, and tables supporting the assumptions and steps used in each stage of the analysis.

In stage 1, we developed siting constraints on energy infrastructure development that represent increasing levels of ecosystem protection, starting with land and waters that are already protected (Siting Level 1) and expanding protections to areas requiring administrative review for development (Siting Level 2) and finally to areas with high conservation value (Siting Level 3; representing ecological, agricultural, cultural, and other natural resource values; *SI Appendix*, Fig. S2 and Tables S17–S20).

Stage 2 involved spatially explicit resource assessments for onshore wind, offshore wind, utility-scale ground-mounted solar PV, distributed urban-infill PV, and constraining the biomass supply curve. We combined environmental Siting Levels (stage 1) with socioeconomic and technical spatial datasets (*SI Appendix*, Tables S2 and S3), to identify suitable sites for development of each technology and develop a supply curve. For offshore wind and solar PV, gridded data on wind distributions and solar radiation were used to simulate capacity factors. We then applied the Optimal Renewable Energy Build-out (ORB) framework (37) and the MapRE (Multicriteria Analysis and Planning for Renewable Energy) Zoning Tools (38) to create maps of suitable areas and subdivide them into utility-scale candidate project areas (CPAs). Urban-infill PV potential assessment used a different set of techno-economic exclusions (e.g., imperviousness, urban areas; *SI Appendix*, Fig. S4). Different levels of purpose-grown biomass feedstocks (14, 39) were removed in the higher Siting Levels (*SI Appendix*, Tables S1 and S10) and for the Limited Biomass case.

In stage 3, we spatially modeled transmission lines and spur lines (i.e., gen-ties). This involved developing cost and line routing surfaces using the environmental data from stage 1 and techno-economic data representing siting criteria such as slope, terrain, and wildfire risk. Multipliers based on these data were used to represent the relative difficulty and cost of power line siting over diverse terrains (*SI Appendix*, Table S7). For interstate transmission, substation start- and end-points in each state (*SI Appendix*, Fig. S6) were used in least-cost path analysis to generate routes (*SI Appendix*, Figs. S8 and S11). We updated the initial corridor transmission capacities, identified congestion levels, and determined the feasibility of upgrades, reconductoring, and new HVDC and HVAC lines (*SI Appendix*, Table S4). We used the cost surface and substation requirements to estimate costs of each transmission supply option and spur line.

For stage 4, we used the outputs of stages 2 and 3 to develop energy portfolios using the EnergyPATHWAYS (EP) and RIO models (5). EP is a detailed stock-rollover accounting model that tracks infrastructure stocks, energy demand by type, and cost every year for all energy-consuming technologies, as new stocks replace old stocks over time (for example, battery electric vehicles replacing internal combustion engine vehicles). Time-varying electricity and fuel demand outputs from EP were then input into RIO, a linear programming model that combines capacity expansion with sequential hourly operations over a sampling of representative days to find the lowest-cost solution for decarbonized energy supply. The energy system modeling included multiple scenarios that reached net-zero greenhouse gas emissions economy-wide in the 11 western states and two scenarios that reached net zero in the electricity sector only. *SI Appendix*, Table S10 for descriptions of all cases and scenarios. While all energy sector emissions have been accounted for in RIO and the Land Use and Carbon Scenario Simulator (LUCAS) model (40) was used to estimate the net emissions from the land sector, we did not account for potential net emissions from land use and land cover change resulting from the production of purpose-grown biomass (which is only possible under Siting Level 1) due to the low spatial resolution of the biomass supply curve used (39).

In stage 5, we downscaled the quantities of onshore wind, offshore wind, solar PV, and transmission in each RIO portfolio (outputs of stage 4). Biomass demand was disaggregated into specific feedstocks in order to estimate land use requirements for purpose-grown biomass (*SI Appendix*, Table S14). Onshore wind and solar were downscaled using an empirical, random forest approach implemented in R. This approach brought more factors into the downscaling than levelized cost alone, by extrapolating historic siting trends using multiple possible siting criteria (e.g., distance to nearest substation, capacity factor, environmental sensitivity; *SI Appendix*, Table S11). Offshore wind sites were selected based on total levelized cost since there are no existing offshore wind farms along the western US coastline for empirical analysis. For downscaling transmission, a load-carrying capacity threshold was applied to determine whether a line should be built (*SI Appendix*, Table S12).

Finally, in stage 6, we assessed the potential environmental and social impacts of each downscaled portfolio (results of stage 5). We estimated the area of each land cover type or ecological metric impacted by wind, solar, or power line development (*SI Appendix*, Table S16). For social metrics, we calculated the area-weighted average median income, percent living below poverty, percent unemployed, and population density for each infrastructure type. Finally, we estimated the human population residing within several buffered distances of each infrastructure type.

## Supplementary Material

Appendix 01 (PDF)

## Data Availability

Generated key inputs (environmental exclusion categories) and outputs (e.g., renewable resource areas) are available for download on Zenodo (DOI: 10.5281/zenodo.7460026). Historical Ventyx spatial data on the U.S. transmission network used in the regression-based downscaling stage of the analysis are part of a proprietary subscription-based dataset, called the Velocity Suite, that was purchased under a non-disclosure agreement. This dataset is available for anyone to purchase using the following link: https://new.abb.com/enterprise-software/energy-portfolio-management/market-intelligence-services/velocity-suite. The selected project area results associated with a given scenario identify possible locations for new energy generation based on the criteria selected by the authors and using regression methods. This study is based on scenario analysis and is not a siting study capable of making prescriptions or predictions of which areas will or should be developed. However, many of these lands are privately-owned so the data could easily be mis-interpreted by users or landowners as identifying lands which are targeted or sanctioned for renewable energy development by the organizations involved in the study. These data are not publicly available due to the risk of mis-interpretation and the legal and ethical risks associated with a possible change in market value associated with this identification. However, the code used to generate these selected project areas, i.e., the site selection methods and process are publicly available on Zenodo (linked with data).
