# Supplementary material for: Minimizing habitat conflicts in meeting net-zero energy targets in the western United States
Source: Proc Natl Acad Sci U S A. 2023 Jan 19;120(4):e2204098120. doi: 10.1073/pnas.2204098120 (PMC9942791; doi:10.1073/pnas.2204098120)
Supplement: Supplementary file 1 — Appendix 01 (PDF) [file pnas.2204098120.sapp.pdf]

Supporting Information

Minimizing habitat conflicts in meeting net zero energy targets in the Western United States

Grace C. Wu*, Ryan A. Jones, Emily Leslie, James H. Williams, Andrew Pascale, Erica Brand, Sophie S. Parker, Brian S. Cohen, Joseph E. Fargione, Julia Prochnik, Maya Batres, Mary G. Gleason, Michael H. Schindel, Benjamin M. Sleeter, Charlotte K. Stanley

***Corresponding author. E-mail: gracecwu@ucsb.edu**

This PDF file includes:

Supplementary text
Figs. S1 to S29
Tables S1 to S21
SI References

Contents

1 Access to data, code, and results **2**
- A Input data . . . 2
- B Code . . . 2
- C Results . . . 2

2 Additional methods **3**
- A Stage 1: Development of environmental exclusions and Siting Levels . . . 3
  - A.1 Overview . . . 3
  - A.2 Environmental exclusion categories . . . 3
  - A.3 Environmental Siting Levels . . . 5
- B Stage 2. Renewable resource assessment and supply curve development . . . 5
  - B.1 Utility-scale solar, onshore wind, and offshore wind . . . 5
  - B.2 Urban infill solar and rooftop PV . . . 6
  - B.3 Biomass assumptions . . . 7
  - B.4 Accounting for existing power plant footprints . . . 10
- C Step 3. Power line modeling . . . 10
  - C.1 Overview . . . 10
  - C.2 Inter-state transmission modeling . . . 10
  - C.3 Spur line modeling . . . 18
- D Step 4. Energy modeling . . . 21
  - D.1 Creating supply curves from candidate project areas . . . 22
  - D.2 Scenarios . . . 22
- E Stage 5. Downscaling and land use estimates . . . 23
  - E.1 Onshore wind and utility-scale solar . . . 23
  - E.2 Offshore wind . . . 26
  - E.3 Power lines . . . 26
  - E.4 Land use estimates for other technologies . . . 26
- F Stage 6. Strategic environmental assessment . . . 27

3 Additional results **35**
- A Energy modeling results . . . 35
- B Sensitivity results . . . 35

Supporting Information Text

1. Access to data, code, and results

A. Input data. The Renewable Resource Areas (Fig. S3) and Environmental Exclusion (Fig. S2) spatial data will be available for download upon publication of this paper. The data will also be presented in an interactive online webmap.

Historical Ventyx spatial data on the U.S. transmission network used in the regression-based downscaling stage of the analysis are part of a proprietary subscription-based dataset, called the Velocity Suite, that was purchased under a non-disclosure agreement. This dataset is available for anyone to purchase using the following link: https://new.abb.com/enterprise-software/energy-portfolio-management/market-intelligence-services/velocity-suite

B. Code. Code to perform ORB (Optimal Renewable Energy Build-out) analysis, including resource assessment (site suitability; Step 2), site selection (Step 4), and strategic environmental assessment (Step 5) will be available on the following Github repository upon the publication of the paper: https://github.com/grace-cc-wu/PoPWest. A generic version of the site suitability model using only raster inputs and the model used to create candidate project areas are available for free (open source) on the MapRE website as Script Tool B-1 and B-2, respectively.

C. Results. All generated results will be publicly available on https://github.com/grace-cc-wu/PoPWest except the selected project area locations. The selected project area results associated with a given scenario identify possible locations for new energy generation based on the criteria selected by the authors and using regression methods. This study is based on scenario analysis and is not a siting study capable of making prescriptions or predictions of where which areas will or should be developed. However, many of these lands are privately-owned so the data could easily be mis-interpreted by users or landowners as identifying lands which are targeted or sanctioned for renewable energy development by the organizations involved in the study. These data are not publicly available due to the risk of mis-interpretation and the legal and political risks associated with a possible change in market value associated with this identification.

2. Additional methods

A. Stage 1: Development of environmental exclusions and Siting Levels.

A.1. Overview. Stage 1 was the development of siting constraints on energy infrastructure development that represent increasing levels of ecosystem protection, starting with land and waters that are already protected and expanding protections to areas not currently protected. This information was compiled based on the expert knowledge of the co-authors in consultation with other scientists at the state chapters of The Nature Conservancy (TNC) in each of the eleven western states and other stakeholders in these states.

A.2. Environmental exclusion categories. The compilation of environmental data for this study followed methods and conventions used in the original Power of Place study (1, 2) and other prior work (3–8). We separated our study area into two geographic realms: terrestrial and marine. For the terrestrial realm, we began with the final list of 168 data layers used in the original Power of Place study and updated these to incorporate changes made by external parties in datasets over time. For the marine realm, we used data from publicly available sources and expert guidance from TNC marine and geospatial scientists to sort and compile data. As our analysis only considers wind power technologies offshore, we included data pertinent to informing environmental exclusions only for this technology type in the marine realm.

For each realm, we aggregated environmental data into three categories, which we refer to as Environmental Exclusion Categories. The categories range from lands or waters with high levels of protection due to legal restrictions that prevent development (Category 1), to lands or waters that have low to no formal protection yet high conservation values (Category 3). The definitions of the three Environmental Exclusion Categories for the terrestrial and marine realms are as follows (see Tables S17–S19 and the full spreadsheet linked here for an exhaustive list of individual datasets in each Category):

For the terrestrial realm:

- **Category 1 (Legally protected)** Areas with existing legal restrictions against energy development. (Examples: National Wildlife Refuge, National Parks)

- **Category 2 (Administratively protected)** Areas where the siting of energy requires consultation or triggers a review process to primarily protect ecological values, cultural values, or natural characteristics. This Category includes areas with existing administrative and legal designations by federal or state public agencies where state or federal law requires consultation or review. Lands owned by non-governmental organizations (NGOs) that have conservation obligations are also included in this Category. Multiple-use federal lands such as Forest Service lands without additional designations were not included in this Category, although in some prior studies they have been. (Examples: Critical Habitat for Threatened or Endangered Species, Sage Grouse Priority Habitat Management Areas, vernal pools, and Wetlands)

- **Category 3 (High conservation value)** Areas with high conservation value as determined through multi-state or ecoregional analysis (e.g., state, federal, academic, NGO) primarily characterizing the ecological characteristics of a location or wildlife corridor. This category may also include lands that have social, economic, or cultural value. Prime farmlands as determined by the U.S. Department of Agriculture (USDA) are also included in this Category (though they were only included as a solar exclusion, not as a wind exclusion). Despite their conservation value, these lands typically do not have formal conservation protections. (Examples: Prime Farmland, Important Bird Areas, big game priority habitat and corridors, The Nature Conservancy Ecologically Core Areas)

For the marine realm:

- **Category 1 (Legally protected)**: Areas with existing legal restrictions against energy development, or where development would be very difficult based on assumptions about the underlying intentions of the designations for these areas. (Examples: State and Federal Marine Reserves, National Marine Sanctuaries)

- **Category 2 (Administratively protected with a high level of review)**: Locations that require a higher level of approval/review due to existing restrictions. Areas where the seafloor is protected and anchoring of wind infrastructure may trigger review.

- **Category 3 (Administratively protected with a low level of review or no review)**: Locations that require less approval/review for development than those contained in Category 2, and other areas of high conservation value.

In contrast with Wu et al. (2020) (1), we elected to use three Environmental Exclusion Categories instead of four. This was necessary to limit the number of scenarios run in Energy Pathways and RIO modeling. The original Power of Place Category 4 lands captured Landscape Intactness. These were lands without formal conservation protection that have potential conservation value based on their contribution to intact landscape structure, including lands that maintain habitat connectivity or have high landscape intactness (low habitat fragmentation). Most of the lands (85%) captured in the original Power of Place study's Category 4 were incorporated into Category 3 lands in this analysis through the inclusion of data from TNC's Resilient and Connected Network (RNC) (9). The following data from the RCN were included in Category 3 for all 11 states:

- 10 Resilient, Diffuse Flow (Climate Informed), Recognized Biodiversity Value

- 30 Resilient, Diffuse Flow, Recognized Biodiversity

- 50 Resilient, Concentrated Flow (Climate Informed), Recognized Biodiversity
- 60 Resilient, Concentrated Flow (Climate Informed)
- 70 Mostly Resilient, Concentrated Flow, Recognized Biodiversity
- 80 Mostly Resilient, Concentrated Flow
- 91 Resilient, Recognized Biodiversity

Additionally, we included the following layers from the RCN into Category 3 for the Pacific Northwest RCN area (Oregon and Washington) only:

- 20 Resilient, Diffuse Flow (Climate Informed)
- 40 Resilient, Diffuse Flow

The draft list of data layers and categorization decisions were subjected to several rounds of review, and comments were incorporated from The Nature Conservancy (TNC) state chapters and select peer NGOs. After review and refinement, we generated a final list of 180 data layers for Categories 1, 2, and 3 (SM Tables S17–S19). See the full spreadsheet linked here for more detailed descriptions of data, rationale for their categorization, and their sources. For each of the three Categories in both the terrestrial and marine realms, the constituent data layers were aggregated into a single layer. These three aggregated layers were applied in the site suitability analysis (Stage 2) and in Stage 6: Strategic Impact Assessment.

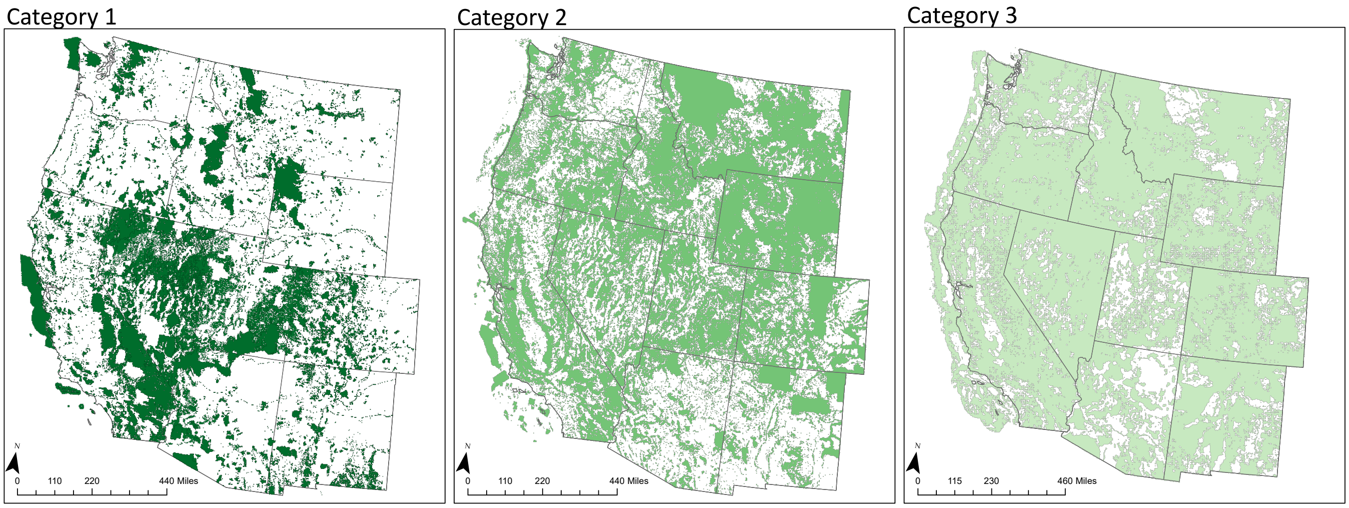

Fig. S1. Environmental Exclusion Categories

Indigenous Lands We recognize the sovereignty of Indigenous Nations, and supports Indigenous Nations in their self-determination and self-sufficiency, in all cases, including when it comes to energy decisions and development. In respect of the sovereignty of indigenous nations, indigenous lands are not included in the analysis. Indigenous lands cover about 50 million acres and represent about 6.25% of the study area.

The datasets that were used in the original Power of Place that were excluded from this analysis included:

- US Census Bureau, Tribal Lands, Cartographic Boundary Shapefiles - American Indian/Alaska Native Areas/Hawaiian Home Lands, https://www.census.gov/geo/maps-data/data/cbf/cbf_aiannh.html
- USGS Protected Areas Database of the US, PAD-US, Native Allotments, Native Allotments provide for the division of tribally held lands into individually-owned parcels, https://gapanalysis.usgs.gov/padus/
- USGS Protected Areas Database of the US, PAD-US, Native American Lands, Federal territory managed by Native American tribes for the Bureau of Indian Affairs, https://gapanalysis.usgs.gov/padus/
- WSDOT, WSDOT - Tribal Reservation and Trust Lands1, http://www.wsdot.wa.gov/mapsdata/geodatacatalog/default.htm

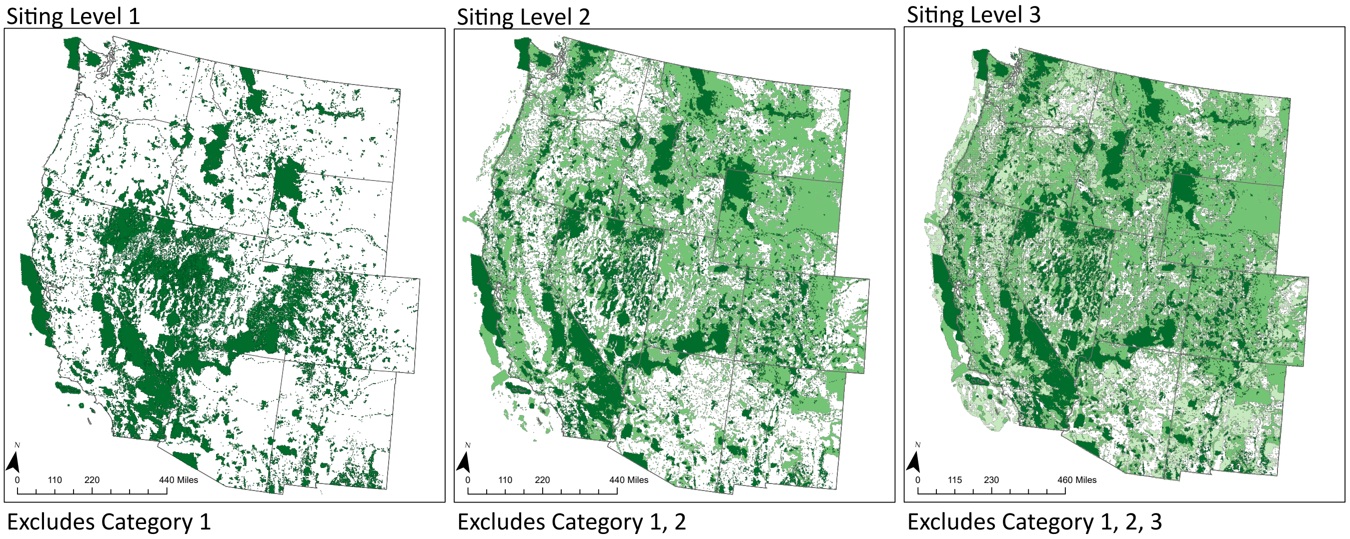

Fig. S2. Environmental Siting Levels

A.3. Environmental Siting Levels. Using the three Environmental Exclusion Categories and the technical and economically suitable areas, we created four supply curves, which are referred to as Siting Levels (SL) 1, 2, and 3 (Fig. S2). All Siting Levels use the same set of technical and economically suitable areas, but are additive in their use of the Environmental Exclusion Categories. That is, Siting Level 1 excludes only land area datasets in Category 1; Siting Level 2 excludes land area datasets in Categories 1 and 2; and Siting Level 3 excludes land area datasets in Categories 1, 2, and 3. As such, as the Siting Level increases, more land is protected from development.

B. Stage 2. Renewable resource assessment and supply curve development.

B.1. Utility-scale solar, onshore wind, and offshore wind. Stage 2 involved conducting spatially-explicit resource assessments for the following renewable energy technologies: onshore wind, offshore wind, utility-scale ground-mounted solar PV, and distributed urban-infill PV. Additionally, we used existing studies to construct supply curves for biomass and adoption assumptions for rooftop PV.

Capacity factor estimates For offshore wind, we calculated annual average capacity factors for each gridded cell within the WIND Toolkit Offshore Summary Dataset using 7 year average weilbull parameters at 100m hub height and the NREL 5 MW offshore reference turbine power curve (from the System Advisory Model (10)). Because the WIND Toolkit offshore wind dataset only covers federal waters about 50 nautical miles offshore, we interpolated the capacity factor values for state waters, which extends 5-10 km offshore, using an inverse distance weighting approach. While an imperfect approach, very few CPAs exist in state waters after applying the siting exclusions, so we expect the method for estimating CFs in state waters to have little impact on the overall offshore wind supply curve. We then used SAM to estimate wake losses using the following parameter inputs: NREL 5 MW reference offshore turbine, the Park (WaSP) wake effects model with 0.1 turbulence, a 100 MW wind farm, and Northern California and Northwestern Oregon AWS truepower representative wind profiles. This yielded a wake effects loss of about 8.9%. Other losses included availability (5.5%), turbine performance (3.95%), and environmental (2.39%).

For utility scale, ground-mounted solar, we used the SAM and the the Physical Solar Model (PSM) version 3 of the National Solar Radiation Database (NSRDB) to estimate annual average CFs for each 10 km grid cell assuming single axis tracking solar PV technology. We estimated capacity factors for 2010 and 2011 (two of the three historic demand demand years in RIO) and then averaged the two years. We used the NSRDB API and the SAM Python code generator (PySSC Python library) to automate the process. For SAM technology parameters, we used the Yingli Energy China 275-35B module with Emerson SPV-5.0 inverter, an inverter loading ratio of 1.4, 1-axis tracking with no tilt, 5% soiling losses, 1% AC wiring loss, 0.5% transformer load loss, no transmission losses (which will be modeled as part of spur line modeling), 1% AC system availability losses, and 0.5% DC degradation rate.

For onshore wind, we used the annual average capacity factors calculated by NREL using the full WIND Toolkit dataset, which has national continuous coverage (11). CFs were estimated based on three representative turbines, selected at each site based on annual average wind speeds at 80 m hub height. The 16.7% losses applied to the CFs (15% losses and 98% availability) includes array wake losses, electrical collection and transmission losses, and blade soiling losses.

Creating Candidate Project Areas For identifying suitable sites and creating candidate project areas (CPAs), we closely follow the approach described in Wu et al. 2020 (1). We restate those methods here to facilitate reader understanding, noting where

modifications have been made. In order to identify suitable areas, we used Stage 1 of the MapRE (Multi-criteria Analysis for Planning Renewable Energy) Zoning Tool (12), which uses Python raster-based algebraic geoprocessing functions and siting assumptions specified for each dataset and technology (Tables S2 - S3). MapRE Zone Tools are the graphical user interface version of the Optimal Renewable energy Build-out (ORB) tools and are part of the ORB suite of siting tools. Using the MapRE Script Tool B-1, we created a single 250 meter resolution raster of areas that satisfy techno-economic siting criteria for each technology (i.e., suitability map). Techno-economic exclusions (Tables S2 - S3) used were informed by prior potential studies (1, 3, 6, 7, 13). For each technology, we removed the Environmental Exclusion Categories from the techno-economic suitability map using raster geoprocessing in Python to create three Siting Levels (SL) of suitable areas that meet both techno-economic and environmental siting criteria.

In order to simulate potential project locations within suitable areas identified, we used MapRE Script Tool B-2, or the "project creation stage", to create Candidate Project Areas (CPAs) by subdividing suitable areas into smaller, utility-scale project-sized areas. Solar potential project areas ranged from 2 km^2 to 9 km^2 (or about 30–270 MW), with the vast majority of solar CPAs designed to be 3 km^2 or to accommodate approximately 270 MW of solar capacity. Wind CPAs ranged from 2 km^2 to 25 km^2 (or about 6–70 MW), with the vast majority of wind CPAs designed to be 25 km^2 or to accommodate approximately 70 MW of wind capacity. We eliminated CPAs less than 2 km^2, as these parcels would typically be considered too small for commercial utility-scale renewable energy development. To arrive at these capacity estimates per CPA we used the following land use efficiency factors: 2.7 MW/km^2 for onshore wind (14, 15), 30 MW/km^2 for solar PV, and 5.2 MW/km^2 for offshore wind (after reviewing the literature on offshore wind power density values, which are primarily based on wind farms in Europe (16).

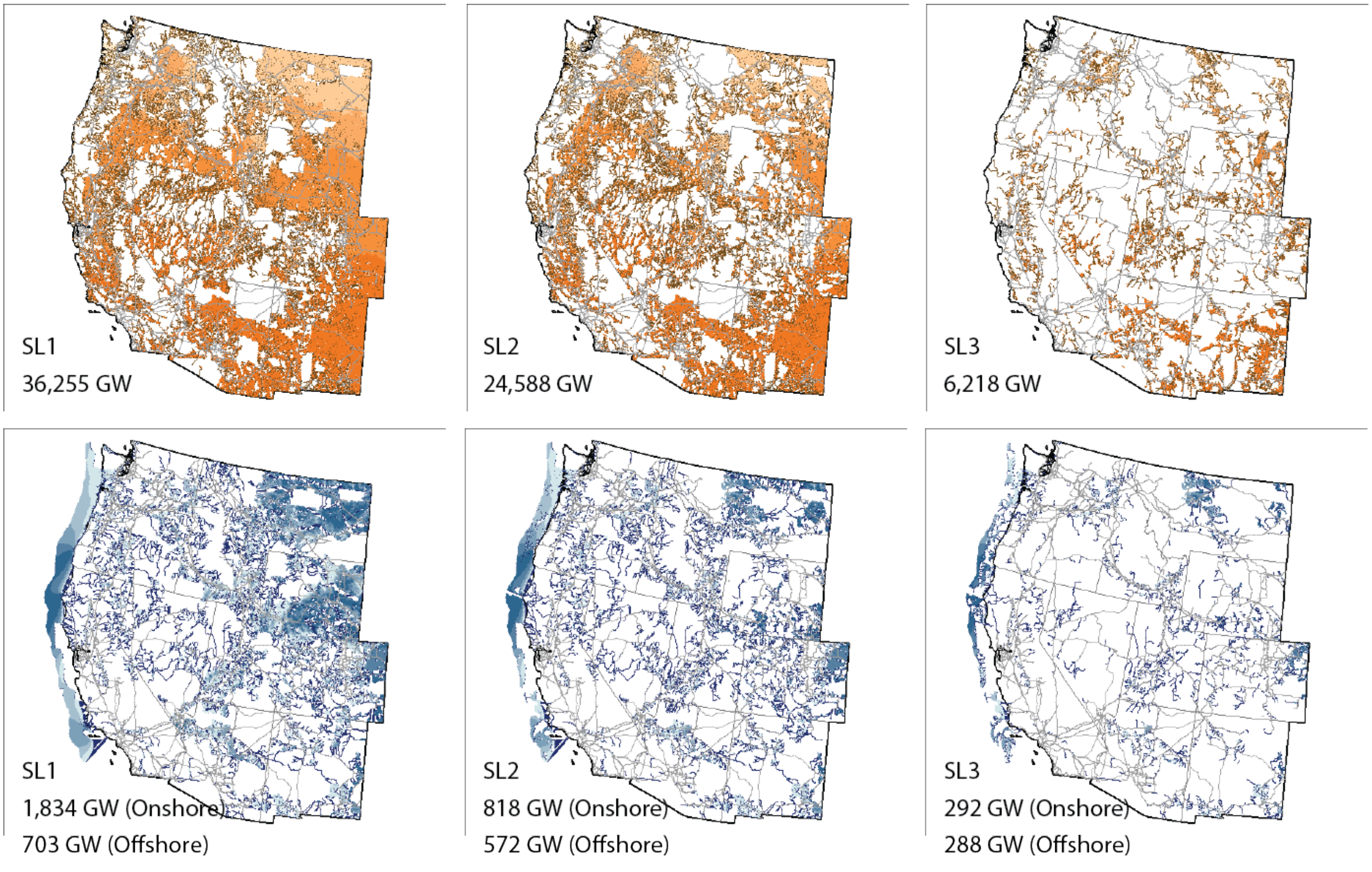

Fig. S3. Utility-scale Wind and solar Candidate Project Areas and spur lines

B.2. Urban infill solar and rooftop PV.

Rooftop PV assumptions The high rooftop PV forecast for 2050 from the 2016 California Energy Commission's Integrated Energy Policy Report (IEPR) (17) represents about 25.7% of technical potential, using NREL's estimate for technical potential of rooftop PV in California of 128.9 GW (18). The IEPR high adoption forecast, which assumes both faster adoption rates and cost declines, as well as higher adherence to Title 24 (or that 90% of new houses built after 2020 install rooftop solar), is considered to be optimistic yet realistic. The independent 2018 Distribution Working Group Forecast Report by Itron corroborates the assumptions driving the IEPR forecast. We selected this value—25% of technical rooftop potential—as the

exogenous RIO input assumption for rooftop solar adoption in SL2, and decreased it by 10 percentage points to 15% for SL1, which has lower renewable capacity requirements, and increased it by 10 percentage points to 35% for SL3, which has more renewable capacity requirements and considered the environmentally preferred Siting Level.

Urban infill assumptions For the characterization of distributed solar resource potential within urban areas and densely populated areas, methods were consistent with Lopez et al (**?**). Urban infill solar includes resource potential located in urban areas, defined by the U.S. Census Bureau as densely developed territory, encompassing residential, commercial, and other nonresidential urban land uses. Two types of urban areas were included: urbanized areas (UAs) that contain 50,000 or more people and urban clusters (UCs) that contain at least 2,500 people. The goal was to identify candidate locations for ground-mount solar arrays within the boundaries of more densely populated areas. Within urban areas, locations with imperviousness >=1 % (USGS NLCD) were assumed to represent buildings, roads, parking lots and other structures and these were therefore treated as an exclusion in site suitability analysis. Parks and landmarks (PAD-US), and wetlands, water bodies, and forested areas (USGS NLCD) were excluded as well. CPAs were designed to be 0.018 to 1 km^2 or to accommodate approximately 0.5-30 MW of solar capacity. See Fig. S4 for examples areas where urban solar infill CPAs have been identified.

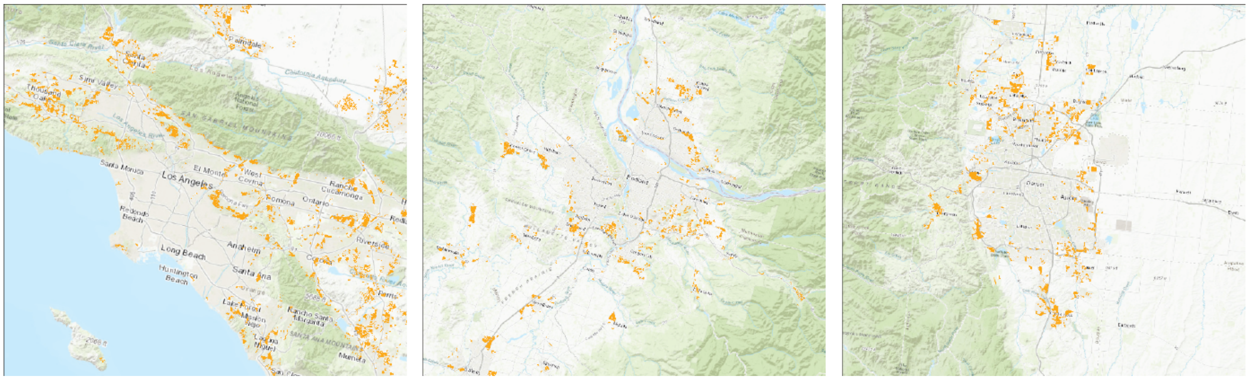

Fig. S4. Example urban infill solar Candidate Project Areas: 703 GW total

B.3. Biomass assumptions. We used the biomass supply curve from the 2016 Billion Ton Study (19) and modified it based on differing levels of sustainability of various feedstocks and where purpose-grown feedstocks are sourced (see Table S1). In Siting Level 1 (SL1), we included the entire, unmodified supply curve from BTS2016. In Siting Level 2 and 3 (SL2, SL3), we excluded all purpose-grown biomass (e.g., biomass sorghum, poplar, other planted whole trees) except miscanthus and switchgrass that can be grown on agricultural lands currently cultivating corn for corn ethanol. This follows the methods and assumptions originally developed by the Net Zero America study (20) and used in several of their scenarios. However, unlike NZA, we excluded the supply within conservation reserve program lands due to its conservation value. We additionally created a Limited Biomass scenario that limited biomass supply to just wastes and residues, thus excluding all miscanthus and switchgrass (Table S1). We note that because land sink accounting and biomass assumptions are not coupled within the same model (see Methods section in the main paper for a description of land sink calculations), the possible conversion of land for purpose grown biomass is not accounted for in land sink assumptions in SL1 (which may lead to an overestimate of the land sink) and the possible additional sequestration of a net contraction in land for purpose grown biomass is not accounted for in the Limited Biomass scenario (which may lead to an underestimate of the land sink).

Table S1. Biomass assumptions for siting levels and sensitivities

			Siting Levels and sensitivities		
Resource Form	Resource	Dry Tons	SL1	SL2 and SL3	Limited biomass
Herbaceous	Miscanthus	253	1	Corn ethanol	0
Herbaceous	Corn stover	170	1	0.62	0.62
Herbaceous	Switchgrass	143	1	Corn ethanol	0
Herbaceous	Biomass sorghum	96	1	0	0
Woody	Poplar	54	1	0	0
Woody	Willow	26	1	0	0
Herbaceous	Wheat straw	22	1	1	1
Waste	Plastics	20	1	0	0
Woody	Hardwood, upland whole trees	18	1	0	0
Waste	Paper and paperboard	16	1	1	1
Woody	Hardwood, lowland whole trees	13	1	0	0
Woody	Other forest residue	13	1	1	1
Waste	Textiles	8	1	1	1
Woody	Softwood, planted whole trees	8	1	0	0
Woody	Softwood, natural whole trees	8	1	0	0
Waste	Food waste	8	1	1	1
Herbaceous	Rice straw	6	1	1	1
Woody	Other forest thinnings	5	1	1	1
Herbaceous	Cotton residue	5	1	1	1
Woody	Softwood, natural logging residues	5	1	1	1
Waste	Rubber and leather	4	1	1	1
Herbaceous	Energy cane	4	1	0	0
Woody	Secondary mill residue	4	1	1	1
Woody	Softwood, planted logging residues	4	1	1	1
Waste	Yard trimmings	4	1	1	1
Herbaceous	Sugarcane bagasse	4	1	1	1
Woody	Hardwood, lowland logging residues	4	1	1	1
Woody	Hardwood, upland logging residues	4	1	1	1
Waste	Noncitrus residues	3	1	1	1
Woody	Mixedwood logging residues	2	1	1	1
Woody	Mixedwood whole trees	2	1	0	0
Herbaceous	Cotton gin trash	2	1	1	1
Waste	Tree nut residues	2	1	1	1
Waste	Citrus residues	2	1	1	1
Herbaceous	Rice hulls	2	1	1	1
Herbaceous	Sorghum stubble	1	1	1	1
Woody	Eucalyptus	1	1	0	0
Herbaceous	Sugarcane trash	1	1	1	1
Herbaceous	Barley straw	1	1	1	1
Woody	Primary mill residue	0	1	1	1
Woody	Pine	0	1	1	1
Herbaceous	Oats straw	0	1	1	1
Waste	Existing Uses	30	1	1	1
Woody	Existing Uses	171	1	1	1

Table S2. Techno-economic datasets for site suitability modeling of onshore wind and utility-scale solar PV

Broad category	Dataset name	Source	Website	Description	Data type/ resolution	Threshold or buffer
Renewable resource	NREL ReV model	NREL	https://www.nrel.gov/docs/fy19osti/73067.pdf	Point locations of simulated wind speeds and estimated annual average capacity factors of quality wind resource areas in the U.S.	CSV with geographic coordinates/ 2 km	Include all areas
Renewable resource	Solar PV capacity factors	NREL	https://sam.nrel.gov/	Point locations of estimated annual average capacity factors for fixed tilt solar PV calculated using SAM *	CSV with geographic coordinates/ 10 km	Include all areas
Technical constraint	Slope	CGIAR	http://www.cgiar-csi.org/data/srtm-90m-digital-elevation-database-v4-1	Calculated slope in percentage from STRM digital elevation model - Resampled 250 m SRTM 90m Digital Elevation Database v4.1	Raster/ 250m	Solar: exclude $>$10%, Wind: exclude $>$19%
Physical constraint	Water bodies and rivers	Argonne National Lab Energy Zones Mapping Tool	https://ezmt.anl.gov/	Permanent water bodies in the U.S. (lakes and rivers)	Shapefile	Wind and solar: include areas $>$250m outside of water bodies
Socio-economic constraint	Census urban zones	2017 TIGER/ Line®	https://www.census.gov/geo/maps-data/data/tiger-line.html	Urban areas as defined by the U.S. Census	Shapefile	Solar: include areas $>$500m, Wind: include areas $>$1000m
Socio-economic constraint	Population density	ORNL Landscan	https://landscan.ornl.gov/	Persons per km^2	Raster/ 1km	Wind and solar: include areas $<$100 persons/km^2
Socio-economic constraint	Military areas	FAA, Argonne National Lab Energy Zones Mapping Tool (EZMT) and West-wide wind mapping project (WWWMP)	https://ais-faa.opendata.arcgis.com/datasets/0c6899de28af447c801231ed7ba7baa6_0?geometry=59.120%2C-23.069%2C-126.857%2C69.964 https://ezmt.anl.gov/ http://wwmp.anl.gov/maps-data/	Includes the following areas: Military Training Routes, Military Installations, Ranges, and Training Routes, DOD High Risk of Adverse Impact Zones, DOD Restricted Airspace and Military Training Routes, Utah Test and Training Range	Shapefile	Solar: include areas $>$1000m, Wind: include areas $>$5000m
Socio-economic constraint	Military areas	Protected Areas Database–U.S.	https://gapanalysis.usgs.gov/padus/	Filtered PAD-US feature class using: Des_Tp = 'MIL'	Geodatabase feature class	Solar: include areas $>$1000m, Wind: include areas $>$5000m
Hazardous constraint	Active mines	USGS Active mines and mineral plans in the U.S.	https://mrdata.usgs.gov/mineplant/	Mine plants and operations for commodities monitored by the National Minerals Information Center of the USGS. Operations included are those considered active in 2003 and surveyed by the USGS.	CSV of geographic coordinates	Wind and solar: include areas $>$1000m
Hazardous constraint	Airports and runways	National Transportation Atlas Database (NTAD) from the U.S. Department of Transportation (USDOT) and Bureau of Transportation Statistics	https://ezmt.anl.gov/	The airports dataset including other aviation facilities is as of July 13, 2018, and is part of the U.S. Department of Transportation (USDOT)/Bureau of Transportation Statistics' (BTS') National Transportation Atlas Database (NTAD).	Shapefile	Solar: include areas $>$1000m, Wind: include areas $>$5000m
Hazardous constraint	Special Use Airspace	Federal Aviation Administration (FAA)	https://hub.arcgis.com/datasets/dd0d1b726e504137ab3c41b21835d05b_0	Effective Date: 2020. Route Airspace data is published every eight weeks by the U.S. Department of Transportation, Federal Aviation Administration-Aeronautical Information Services.	Shapefile	Solar: include areas $>$0m, Wind: include areas $>$0m
Hazardous constraint	Railways	National Transportation Atlas Database (NTAD) from the U.S.DOT and Bureau of Transporation Statistics	https://ezmt.anl.gov/	The Rail Network is a comprehensive database of North America's railway system at 1:24,000 to 1:100,000 scale as of May 25, 2018.	Shapefile	Wind and Solar: include areas $>$250m
Hazardous constraint	Flood zones	National Flood Hazard (FEMA)	https://www.fema.gov/flood-maps/products-tools/national-flood-hazard-layer		Geodatabase feature class	Wind and Solar: include areas $>$0m

* Solar PV capacity factor calculation assumptions for SAM: Ground Mount Single-Axis Tracking Configuration, DC/AC Ratio = 1.4, Average Annual Soiling Losses = 5%, Module Mismatch Losses = 2%, Diode and Connection Losses = 0.5%, DC Wiring Losses = 1%, AC Wiring Losses = 1%, Availability Losses = 1%, Degradation = 0.5% per year

Table S3. Techno-economic datasets for site suitability modeling of offshore wind

Broad category	Dataset name	Source	Website	Description	Data type/ resolution	Threshold or buffer
Renewable resource	WIND Toolkit dataset for offshore wind	NREL	https://cscdata.nrel.gov/#/datasets /3cb55fd7-57ec-418c-a8ba-181 e4a2779b2	Point locations of simulated weibull parameters in the U.S./ 1-2 km	feature class	Include all areas
Socio-economic constraint	Submarine cables	Argonne National Labs	https://ezmt.anl.gov/	Polyline locations of submarine cables	shapefile	Include areas > 250m
Socio-economic constraint	Military Marine Danger Zones	Argonne National Labs	https://ezmt.anl.gov/		shapefile	Include areas > 5000m
Socio-economic constraint	Shipping lanes	NOAA	http://ftp.coast.noaa.gov/pub/MSP /ORT	Select "THEMELAYER" In('Particularly Sensitive Sea Area', 'Shipping Fairways Lanes and Zones', 'Recommended Routes', 'Traffic Separation Schemes/Traffic Lanes', 'Traffic Separation Schemes')	shapefile	Include areas > 250m
Socio-economic constraint	Oil and gas wells	NOAA	http://ftp.coast.noaa.gov/pub/MSP /ORT		shapefile	Include areas > 250m
Socio-economic constraint	Pipeline areas	NOAA	http://ftp.coast.noaa.gov/pub/MSP /ORT		shapefile	Include areas > 250m
Technology criteria	Bathymetry	NOAA	https://maps.ngdc.noaa.gov/viewers/grid-extract/index.html	Used to determine wind technology (fixed turbine in shallow water < 50m depth, floating turbine in deep water > 50m depth)	shapefile	N/A

B.4. Accounting for existing power plant footprints. The results of the above site suitability modeling steps include maps of possible locations for wind and solar development. For many of these possible locations, however, there are wind and solar power plants that have already been constructed. Existing power plants must be removed from the CPAs and supply curve in order to ensure that the supply curve only contains undeveloped future candidate projects. By removing existing projects, we enable RIO to optimize future capacity expansion investment decisions and avoid overestimating the resource potential.

For existing wind facilities, we used a combination of the U.S.Wind Turbine Database (USGS USWTDB) and U.S. EIA 860 data (Table S15). The USGS wind turbines were grouped by facility name, and then a polygon was identified for each facility, using the "convex hull" technique. The resulting polygons outline the areas where turbines (points) share a facility name. A different approach was used to create polygons from the EIA data, which had facilities identified by a single point per facility. These points were buffered by a radius appropriate to ensure the resulting wind project polygon would have 2.7 MW/km2 power density. Finally, we merged the buffered EIA and additional USWTD polygons to have a gap-filled existing wind turbine footprint dataset. These areas with existing wind turbines were removed from the candidate wind project areas.

For solar resource potential, we used the USGS national solar array footprint dataset (21) combined with the U.S. EIA dataset (Table S15), using the same methods as for wind. These existing solar facilities were removed from the candidate solar project areas.

C. Step 3. Power line modeling.

C.1. Overview. Stage 3 was modeling of transmission lines and spur lines (i.e., gen-ties). The first step in both cases was developing cost and line routing surfaces using the environmental exclusion categories plus techno-economic data representing siting criteria such as slope, terrain, and wildfire risk. Multipliers based on this data were used to represent the relative difficulty and cost of power line siting over diverse terrains (Table S7). For inter-state transmission lines, substation start- and end-points in each state were selected (Fig. S6). This involved updating the initial corridor transmission capacities, identifying congestion levels, and determining the feasibility of upgrades, reconductoring, and new HVDC and HVAC lines (Table S4). Only one start- and end-point was assigned in each state, except for California, which due to its geographic size and demand, was divided into northern and southern zones. For spur lines, CPA locations and existing high voltage substations were selected as start- and end-points for individual project-related spur line builds. The interstate start- and end-points and routing surfaces were then entered into a GIS-based least-cost path tool to generate routes (Figs. S8 and S11). We made realistic estimates of the cost of each spur and inter-state transmission route using the cost surfaces and substation requirements based on transmission length, reflecting the actual developer costs and trade offs of long-distance transmission from remote resources to high-density load centers (Tables S5, S6, S8, S9, Fig. S7). Spur line costs, including the cost of interconnection, were then incorporated into the utility-scale wind and solar supply curves. Inter-state transmission lines were compiled into a lowest-cost ordered supply curve for each state-to-state corridor and passed to the energy models employed in the next stage. The following subsection describe each of these steps in detail.

C.2. Inter-state transmission modeling. We used a combination of transmission costing tools, environmental conservation and risk data, GIS tools and expert opinions to generate transmission corridors for use in the analysis. Figure S5 details the steps

followed to generate transmission corridors.

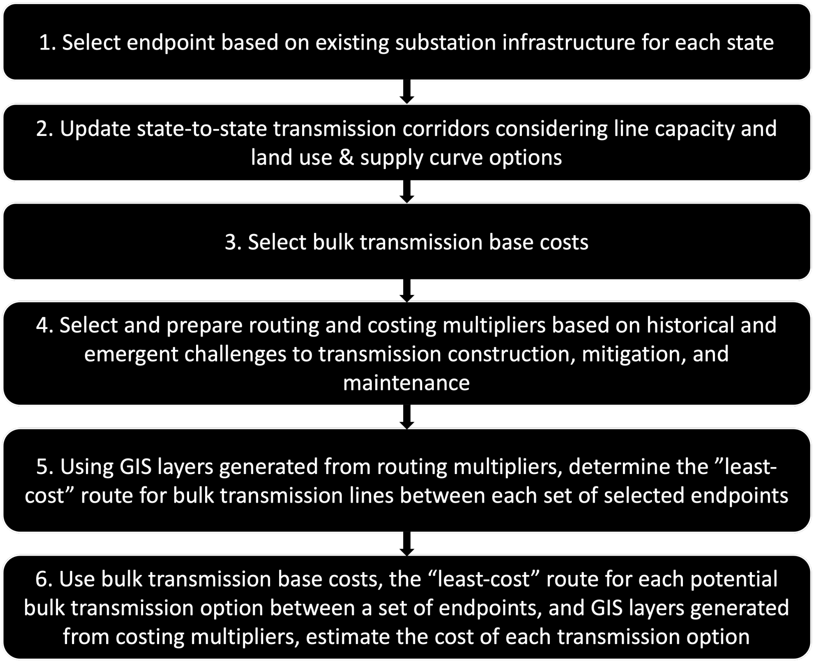

Fig. S5. Transmission modelling flow

Substation selection State representative substations were selected from the HIFLD (22) substation dataset by examining current intersection points of 230kV, 345kV and 500kV transmission lines and pinpointing a substation location in each state. We analyzed and compared the chosen substations to their knowledge of congested areas to validate the interconnection locations. Substation selection included consideration for capacities of existing grid infrastructure, (23), congestion analyses of that infrastructure (24), and expert opinion regarding typical modeling conventions for the western U.S. electric system. Large and widely separated load pockets in California led to the selection of two representative substations for California, while only one representative substation was selected for each of the remaining states. Figure S6 presents a map of those substations.

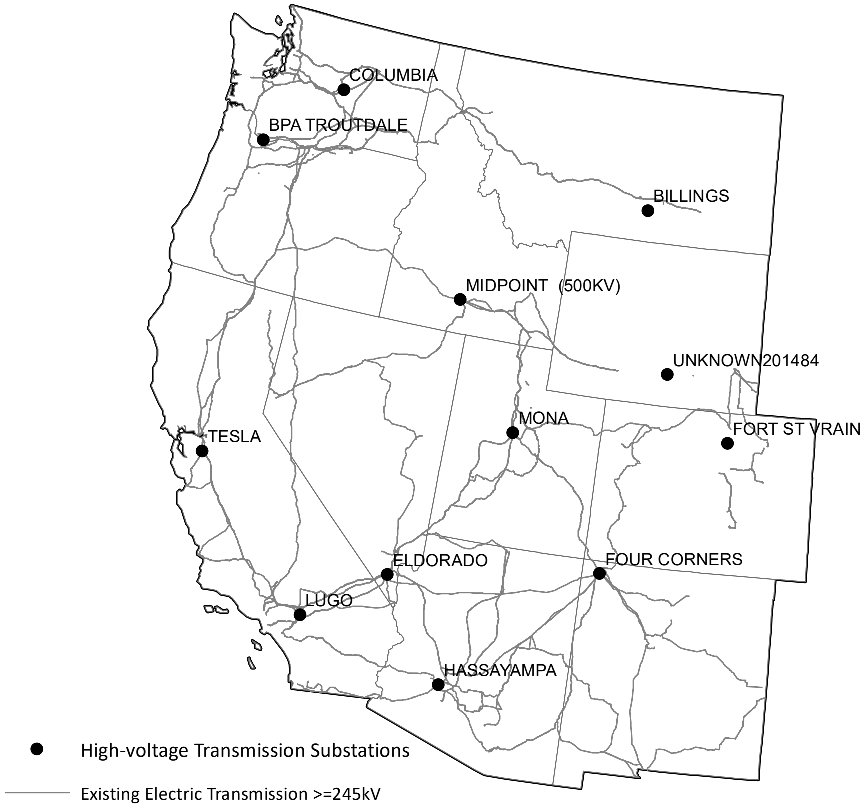

Fig. S6. Selected substations

Update corridor capacities and Identify supply curve options The transmission capacities for each state-to-state corridor in the RIO model were updated using WECC path rating studies (24) (25), OASIS, other reports (26) (27), and HIFLD (22) (28) data. Table S4 lists the 2020 corridor capacities along with the transmission supply options and maximum capacities allowed in each corridor.

Table S4. List of inter-state TX corridors, along with 2020 capacities and the supply options and availability (in MW) allowed

Corridor	Flow / Counterflow capacity (MW)	Re-conductor 230kV (MW)[1]	Re-conductor 345kV (MW)[2]	Re-conductor 500kV (MW)[4]	Co-locate 500kV (MW)[5]	New 500kVd HVAC (MW)[5]	New 500kV HVDC (MW)[5]
arizona to new mexico	2400/2400	0	1500	1500	3000	3000	3000
californiaS to arizona	3767/9600	0	0	1500	3000	3000	3000
californiaS to californiaN	4800/5400	400	750	1500	3000	3000	3000
californiaN to nevada	150/160	0	0	0	3000	3000	3000
californiaS to nevada	4100/7360	800	750	6000	3000[3]	3000	3000
californiaS to utah	1400/2400	0	0	0	3000	3000	3000
idaho to montana	256/337	400	0	0	3000	3000	3000
idaho to utah	1600/1250	400	1500	0	3000	3000	3000
idaho to wyoming	4100/2400	400	750	0	3000	3000	3000
montana to wyoming	400/2598	800	750	1500	3000	3000	3000
nevada to arizona	4785/9600	800	750	1500	3000	3000	3000
nevada to idaho	360/500	0	0	0	3000	3000	3000
nevada to utah	485/500	400	750	1500	3000	3000	3000
new mexico to colorado	664/1970	400	750	0	3000	3000	3000
new mexico to californiaS	2325/0	0	0	0	0	3000	6000
oregon to californiaS	8010/4675	0	0	0	3000	3000	3000
oregon to californiaN	8010/4675	800	0	3000	3000	3000	3000
oregon to idaho	600/1500	800	0	1500	3000	3000	3000
utah to arizona	265/300	0	0	0	3000	3000	3000
utah to colorado	650/650	0	750	0	3000	3000	3000
utah to new mexico	647/700	400	750	0	3000	3000	3000
washington to idaho	1200/2400	400	750	0	3000	3000	3000

Table S4. List of inter-state TX corridors, along with 2020 capacities and the supply options and availability (in MW) allowed

Corridor	Flow / Counterflow capacity (MW)	Re-conductor 230kV (MW)[1]	Re-conductor 345kV (MW)[2]	Re-conductor 500kV (MW)[4]	Co-locate 500kV (MW)[5]	New 500kVd HVAC (MW)[5]	New 500kV HVDC (MW)[5]
washington to montana	1350/2200	400	0	1500	3000	3000	3000
washington to oregon	12000/12000	1200	750	4500	3000	3000	3000
wyoming to californiaS	0/0	0	0	0	0	6000	3000
wyoming to colorado	1605/1400	400	750	1500	3000	3000	3000
wyoming to utah	1800/1600	400	0	0	3000	3000	3000

[1] base capacity addition per line = 400 MW
[2] base capacity addition per line = 750 MW
[3] only line under-grounded due to known lack of space above-ground for a co-location
[4] base capacity addition per line = 1500 MW
[5] base capacity addition per line = 3000 MW
[6] table entries containing a zero were not modelled

Select transmission base costs Base cost for each supply curve option were drawn from the Black & Veatch (B&V) "Transmission Line Capital Cost Calculator" (29) (23) initially created in the Western Electricity Coordinating Council transmission planning forums (TEPPC). Base transmission costs and the calculator settings used to generate base costs are listed in Table S5.

Table S5. B&V Transmission cost calculator configuration and base costs

Configuration	Re-conductor 230 kilovolt (kV)	Re-conductor 345kV	Re-conductor 500kV	Co-locate 500kV	New 500kVd HVAC	New 500kV HVDC
Calculator configuration	230 kV double circuit, ACSR, Lattice, >10 miles, reconductor	345 kV double circuit, ACSR, Lattice, >10 miles, reconductor	500 kV double circuit, ACSR, Lattice, >10 miles, reconductor	500 kV double circuit, ACSR, Lattice, >10 miles, new	500 kV double circuit, ACSR, Lattice, >10 miles, new	500 kV HVDC, ACSR, Lattice, >10 miles, new
Base cost in USD2018/mile	664,127	1,262,297	2,131,048	3,278,535	3,278,535	1,639,820
ROW width in feet	150	200	250	NA	250	200

In order to finalize base transmission costs (without substations), right-of-way land lease costs are drawn from a BLM land cost layer added to all supply curve options in Table S5 except the co-locate option [†]. This process follows the implementation order and method detailed in the B&V Transmission cost calculator. Base substation costs and the calculator settings used to generate the base costs using the Black & Veatch (B&V) "Substation Capital Cost Calculator" (29) (23) are listed in Table S6.

Table S6. B&V Substation calculator configuration and base costs

Parameter	500kV HVAC	500kV HVDC
Sub/converter station handling	minimum 2 substations (start and end), plus one every 100 miles (after first 200 miles)	minimum 2 substations(start and end), plus one every 400 miles (after first 400 miles) B&V calculator configuration for subconverter station costs
500 kV Substation, New, Breaker and a Half, 6, no, 345/500 kV XFMR , 600, 4, 3, 1, 3, 0.175	500 kV Substation, New, Breaker and a Half, 4, 500 kV HVDC Converter , 345/500 kV XFMR , 750, 4, 3, 1, 3, 0.175	
Sub/converter station cost in USD 2018	87,942,834	654,486,168

Select and prepare routing and costing multipliers This section describes the routing and costing multipliers that were used in step 4 in inter-state transmission modeling (Figure S5). Essentially the study area is broken into 250 m grid cells, and each cell has an associated cost (in dollars per mile) to cross the terrain in that cell. The least cost path algorithm is used to identify the route connecting the two end points at minimal cost. Two different versions of the cost surface are used for routing vs. costing, and within each of these two surface types, there are multiple surfaces corresponding to different voltages. The routing and

[†] The GIS layer from BLM to which we have access, only has 10 (of 15) cost tiers implemented. If this study was iterated in the future it would ideally use a layer matching the 15 layers in the B&V transmission cost calculator.

costing multipliers that were selected for use in this study are listed in Table S7. At a high level, the cost multipliers are based on values identified in the WECC TEPPC transmission cost calculator (BV Calculator) and the CAISO PTO per-unit-cost guides (2020). As an example, if the base cost for a 230 kV transmission line is $1 million per mile, and if a certain 250m grid cell contains forested terrain, with a multiplier of 2.25, then the cost of the line crossing that cell would be $2.25 million per mile. Several modifications were made to account for terrain type variables not modeled in existing publicly available tools such as the B&V calculator. Modifications include adding new multipliers to account for areas with high wildfire risk, water bodies, and areas of high environmental conservation value.

Table S7. Transmission routing multipliers

Multiplier	GIS layer	Use	Criteria	Value[1]
Terrain	MRLCD (30)	routing	Forested	2.25
Terrain	MRLCD (30)	routing	Urban	1.59
Terrain	MRLCD (30)	routing	Wetlands (and water)[5]	1.20
Terrain	MRLCD (30)	routing	Desert/barren	1.05
Terrain	MRLCD (30)	routing	Scrubbed/Farmland/(& other)[5]	1.00
Slope	USGS (31)	routing	mountain (greater than 4 degrees)	1.75
Slope	USGS (31)	routing	rolling hills (between 1 and 4 degrees)	1.40
Slope	USGS (31)	routing	flat (less than 1 degree)	1.00
Environmental Risk	The Nature Conservancy	routing	Category 1	100 (TNC)[3]
Environmental Risk	The Nature Conservancy	routing	Category 2	20 (TNC)
Environmental Risk	The Nature Conservancy	routing	Category 3	15 (TNC)
Environmental Risk	The Nature Conservancy	routing	No Category	1 (TNC)
Airports and Runways	EZMT [ref] [ref]	routing	$<$ 5km from either	100 (32)
Existing ROW	HILFD (28)	routing	New builds + in existing ROW	9 (TNC)[7]
Existing ROW	HILFD (28)	routing	Co-locate + outside existing ROW $>=$ 230 kV	9 (TNC)[7]
Existing ROW	HILFD (28)	routing	230 kV reconductor + outside existing ROW $=$ 230 kV	2.22[9]
Existing ROW	HILFD (28)	routing	345 kV reconductor + outside existing ROW $=$ 345 kV	1.82[9]
Existing ROW	HILFD (28)	routing	500 kV reconductor + outside existing ROW $=$ 500 kV	1.54[9]
Tower structure	Population Density, USDOT (33)	both	230 kV + population density $>$ 100 people/square mile	1.1
Tower structure	Population Density, USDOT (33)	both	345 kV + population density $>$ 100 people/square mile	1.3
Tower structure	Population Density, USDOT (33)	both	500 kV + population density $>$ 100 people/square mile	1.5
Wildfire risk	Risk to Potential Structures in USDA Forest Service (34)	both	risk scaled[6]	1 to 5 (TNC)[2]
AFUDC and overhead	continental US	costing	All	1.175
B&V Terrain/Slope	USGS (31) MRLCD (30)	costing	Forested	2.25
B&V Terrain/Slope	USGS (31) MRLCD (30)	costing	Mountain	1.75
B&V Terrain/Slope	USGS (31) MRLCD (30)	costing	Urban	1.59
B&V Terrain/Slope	USGS (31) MRLCD (30)	costing	Rolling hills	1.40
B&V Terrain/Slope	USGS (31) MRLCD (30)	costing	Wetland (& water)[5]	1.20
B&V Terrain/Slope	USGS (31) MRLCD (30)	costing	Desert/barren land	1.05
B&V Terrain/Slope	USGS (31) MRLCD (30)	costing	Scrubbed/Farmland/(& other)[5]	1.00
Environmental Risk	The Nature Conservancy	costing	Category 1	1.2 (TNC)[4]
Environmental Risk	The Nature Conservancy	costing	Category 2	1.1 (TNC)[8](35)
Environmental Risk	The Nature Conservancy	costing	Category 3	1.05 (TNC)[8](35)
Environmental Risk	The Nature Conservancy	costing	No Category	1 (TNC) (35)

Multiplier	GIS layer	Use	Criteria	Value[1]

[1] All values are drawn from B&V tool (23) (29) unless otherwise marked
[2] Our multiplier value for fire risk areas is based on the idea that in order to limit fire risk during operation of the TX lines, the lines would be fire hardened or undergrounded in areas with the highest fire risk. We have capped this multiplier at 5x, which is above the 3.07 – 4.17 x undergrounding multiplier implied by the costs of undergrounding a 66 – 115 kV line according to a California ISO participating transmission owner in 2020 (36) ‡, but is on the lower end of the 3x to 10x multipliers implied by a 2013 undergrounding study from the Edison Electric Institute (37). §
[3] An exception has been made in the routing and costing layers at points where existing transmission right-of-ways cross long and continuous layers – specifically the scenic and historic trails layer within the CAT1 surface. We have removed all CAT1 environmental penalties at such crossings, so that transmission additions/upgrades will use existing crossings.
[4] For possible edge cases where the only route is through a Category 1 exclusion, we have used a 1.2x multiplier.
[5] This was done in part to allow mapping of terrain multipliers onto the selected terrain dataset which contains land categories not included in the B&V calculator, as well as classifies a "Water" category separate from the "Wetlands".
[6] See discussion below table
[7] A multiplier of 9x was chosen arbitrarily, to make existing ROW routing "cost" higher than most multiplier 'stacks' in a surface. For "new" lines, this drives the least-cost-path algorithm away from existing lines, to seek a new path connecting two endpoints. For "co-located" lines, this drives the least cost path algorithm to stay within existing right-of-way. Only when faced with larger multiplier stacks on nearby land parcels will a new transmission path enter an existing ROW with a 9x multiplier, or will an upgrade in an existing transmission corridor deviate from that corridor due to the 9x multiplier on surrounding land parcels.
[8] We increased the WECC mitigation study costs by one order of magnitude due to this text in the report, "In terms of mitigation costs, the study concentrates on a handful of line-item mitigation costs. These capture only a portion of the overall environmental costs of a project. [...] The study, therefore, does not factor in project costs associated with routing alternatives, specialized construction techniques, or the permitting phase—all of which may result in costs that are an order of magnitude greater than those represented in the study." (35, p 43)
[9] The purpose of these multipliers is to allow the least cost path algorithm to jump "gaps" in existing lines of a certain voltage for reconductoring. For example, if a reconductor path can follow an existing 230 kV line for 150 out of 200 mi between two endpoints, but the remainder of the existing line between these two endpoints does not have voltage information or is of a different voltage, then this multiplier would be applied to this segment to encourage the least cost path to follow this existing line anyway. These multiplier values are derived from the difference between new builds and reconductor costs for each voltage level. Due to limited representation of the full extent of higher voltage networks in the dataset used to map existing transmission (22), these multipliers were decreased to allow other multipliers more importance when crossing 'missing' portions of existing transmission networks. When a more accurate layer showing existing transmission becomes available, these multipliers can be increased to the same values used for new builds and co-located lines.

Routing modifications Modification of the Black & Veatch (B&V) tool (29) (23) for routing purposes involves altering and adding to the multipliers listed in the tool. Alterations included the following:

1. Adding a tower structure adjustment multiplier as a GIS layer in order to apply the B&V calculator "Tubular Steel" multiplier based geospatial information. The "Tubular Steel" tower multipliers vary for each voltage class:
   (a) 230 kilovolt (kV): 1.1x [B&V default]
   (b) 345 kV: 1.3x [B&V default]
   (c) 500 kV: 1.5x [B&V default]
2. Moving the slope related multipliers from the terrain section into their own slope type multiplier. This was done for:
   (a) Flat (<2% Slope, < 1 degree): 1.00 x [B&V default/assumed]
   (b) Rolling Hills (2-8% Slope , 1 to 4 degree): 1.40 x [B&V default]
   (c) Mountain (>8% Slope, > 4 degree): 1.75 x [B&V default]
3. Keeping terrain multipliers at their default B&V values, and adding a "water" and "all other type" multipliers.3
   (a) Forested 2.25 x [B&V default]
   (b) Scrubbed 1.00 x [B&V default]
   (c) Wetland 1.20 x [B&V default]
   (d) Farmland 1.00 x [B&V default]
   (e) Desert/barren land 1.05 x [B&V default]
   (f) Urban 1.59 x [B&V default]
   (g) Water (if not designated as wetlands) 1.20 x [assumed] ¶
   (h) All other types 1.00 x [assumed]
4. Adding environmental risk routing multipliers for exclusion categories. Environmental routing multipliers are intended to reflect preference not to allow energy development projects in environmentally sensitive areas (Category 1, Category 2, Category 3) under different scenarios. For transmission routing we have decided to use high multipliers for all three categories in order to eliminate or minimize the distances over which lines are routed in environmentally sensitive areas.
   (a) Category 1- routing 100 x [off limits]

¶ As some rivers appear to be part of the "Water", rather than "Wetlands" category in the selected terrain dataset (30), this category was given the same multiplier as the wetlands category. If we find transmission lines cross open bodies of water like Lake Tahoe, then this handling would need to be revised.

(b) Category 2- routing 20 x [assumed]

(c) Category 3- routing 15 x [assumed]

(d) No category - routing 1 x [assumed]

5. Although techno-economic exclusion zones have been used to limit renewable resource project siting within a buffered distance of railroad corridors, active mine sites, airports, airport runways, areas with high population densities or steep inclines, flood zones, military areas of consideration; many of these areas also often contain high voltage transmission lines. In this analysis, we have only treated airports and airport runways, each buffered with a 5km radius, as techno-economic exclusion zones for transmission. These techno-economic exclusion zones have been given a routing multiplier of 100x.

6. Various multipliers are applied to either encourage or discourage following of right-of-ways (ROW) in the following circumstances:

   (a) For reconductored lines with portions missing at the relevant voltage, environmental multipliers are only applied outside of ROWs having a voltage of 230kV or greater.

   (b) For co-located lines, we have allowed co-location in all ROWs having a voltage of 230 kV or greater. For routing purposes, we minimize the deviation of co-location builds from ROWs by increasing costs outside of ROWs. [‖] For co-located lines with portions missing in the selected ROW dataset, environmental multipliers are only applied during transmission routing, and then only outside of ROWs having a voltage of 230kV or greater

   (c) For all new lines, we use environmental routing and costing multipliers over the entire surface, including in ROWs. [**] In order to bias new builds away from existing ROWs during the routing step of the process, we use new line base costs for all areas outside of existing ROWs, and 9x new line base costs at the same voltage inside of ROWs.

7. Wildfire and environmental risk layers are additional multipliers, and the B&V transmission cost calculator base terrain/slope multipliers are allowed to 'stack' on top of Wildfire and environmental risk multipliers. We based the addition of a fire risk multiplier on the potential for a transmission line to start a fire during its operation and cause damage to the habitats or neighborhoods they cross. Our multiplier value for fire risk areas reflects the idea that in order to limit fire risk during operation of the TX lines, the lines would be fire hardened or undergrounded in areas with the highest fire risk. We have capped this multiplier at 5x, which is above the 3.07 – 4.17 x undergrounding multiplier implied by the costs of undergrounding a 66 – 115 kV line according to a California ISO participating transmission owner reported costs in 2020 (26) [††], but is on the lower end of the 3x to 10x multipliers implied by a 2013 undergrounding study from the Edison Electric Institute (37). [‡‡] The general method for scaling an underlying wildfire risk raster (RR) to our capped fire risk multiplier is shown below:

   $$1 + (maximumofundergrounding/fireHardeningmultiplier) - 1) * RR/(maximum(RR)$$

   This scaling method translates to areas with low fire risk having a multiplier of 1x. As fire risk increases, the fire risk multiplier increases from 1x to a maximum of 5x. This does not make high wildfire hazard areas off-limits for transmission siting, but suggests that transmission routes seeking to pass through a high wildfire hazard potential area would likely need to pursue fire-hardening measures (e.g. ROW widening, increased monitoring and maintenance facilities, or undergrounding) in proportion to the risk of starting a wildfire during operation.

Costing modifications Modification of the Black & Veatch (B&V) tool (29) (23) for costing purposes involves altering and adding to the multipliers listed in the tool. Alterations included the following:

1. Keeping all terrain multipliers at their default B&V values and adding "water" and "all other types" multipliers. [§§]

   (a) Forested 2.25 x [B&V default]

   (b) Mountain 1.75 x [B&V default]

   (c) Urban 1.59 x [B&V default]

   (d) Rolling Hills 1.40 x [B&V default]

   (e) Wetland 1.20 x [B&V default]

[‖] We use new line base costs inside ROWs and use 9x new line base costs at the same voltage outside of ROWs. A multiplier of 9x is chosen arbitrarily but is aimed at being higher than most multiplier stacks in cost surface, while allowing some deviation when warranted by a large cost multiplier stack. For example, a devotion from an existing ROW might occur when the least-cost path algorithm is comparing a 9x multiplier outside of a ROW with a 11.25x multiplier in a ROW running through an area that is forested (2.25x) and is at high risk for wildfires (5x).

[**] An exception has been made in the routing and costing layers at points where existing transmission right-of-ways cross long and continuous layers – specifically the scenic and historic trails layer within the CAT1 surface. We have removed all CAT1 environmental penalties at such crossings.

[††] Only one of six participants – SCE – provided costs which could be used to develop a multiplier. Undergrounding costs were provided for rural/desert and metropolitan areas at 66 kV and 110 kV lines. This is a lower voltage than the 230 kV lines being mapped/costed here for spur-lines, which is one argument for making an underground cost multiplier higher than is suggested by SCE costs.

[‡‡] The study does not provide costs for different high voltage transmission levels but notes the wide variations in costs (and implied multipliers) arises from the large variation in costs of transmission at different voltage levels.

[§§] This was done in part to allow mapping of terrain multipliers onto the selected terrain dataset (30), which contains land categories not included in the B&V calculator, as well as classifies a "Water" category separate from the "Wetlands".

(f) Water (if not designated as wetlands) 1.20 x [assumed] ¶¶

(g) Desert/barren land 1.05 x [B&V default]

(h) Scrubbed & farmland 1.00 x [B&V default]

(i) All other types 1.00 x [assumed]

2. Adding environmental risk cost multipliers for TNC selected exclusion categories. Environmental cost multipliers have been implemented for instances where Category 1, Category 2, and Category 3 areas were encroached on despite disincentives created by high routing multipliers. Cost multipliers are intended to provide reasonable environmental mitigation costs over short distances in such cases. Cost were drawn from a WECC study (5), and inflated one order of magnitude from the 0.5% and 1% multipliers recommended in the WECC study. ***

   (a) Category 1 - costing 1.20 x [assumed] †††

   (b) Category 2 - costing 1.10 x [derived from (5)]

   (c) Category 3 - costing 1.05 x [derived from (5)]

   (d) No category - costing 1.00 x [derived from (5)

3. Using a 1.20x multiplier for techno-economic exclusions (see the footnote on the Category 1 – costing multiplier).

4. Using same fire risk and tower structure adjustment multipliers as in routing process

5. Remove ROW capital costs for co-located projects

6. Add a 5x multiplier for complete/partial undergrounding of long-distance transmission lines.

Determine least-cost route, and use least-cost route to estimate capital cost This section describes steps 5 and 6 in Figure S5, (determine least-cost route, and use least-cost route to estimate the capital cost of each line).

Transmission routing was implemented by combining transmission base costs with routing multipliers in order to generate a routing surface (Base cost * all routing multipliers but AFUDC/overhead + ROW land cost) * AFUDC/overhead multiplier), and then using the ArcGIS least cost path as polyline tool (38) to determine the route of each interstate transmission connection with a supply availability of greater than zero in Table S4.

The cost of each transmission option was then determined by repeating the prior processing using costing multipliers instead of routing multipliers (Base cost * all costing multipliers but AFUDC/overhead + ROW land cost) * AFUDC/overhead multiplier) and constraining transmission paths to the pre-routed options determined in the last step. In a final step, substation costs were added to the cost of each new transmission line using the parameters shown in Table S6. We applied a 5x multiplier to the co-located 500 kV line on the southern California to Nevada corridor to imply its undergrounding - it will go from being the first choice in the supply curve for that corridor, to the second to last choice. The 500 kV TX line crosses a zone with a >500x multiplier (is CAT1 - 100x, fire risk - 5x) for 250 meters. This is because the selected southern California substation is located in a CAT1 zone and quite congested.

Although ideally mapping and costing would be integrated in the process, there are a number of barriers to integration. For the current analysis, mapping will precede costing, and will generally reflect a combination of designed and available policy and economic inputs into line siting. Also note that neither WECC's "capital-cost" modeling tool (29) (23) nor this study factors in the project costs associated with routing alternatives, specialized construction techniques, or the permitting phase - all of which may result in costs that are an order of magnitude greater than those represented in the study.

¶¶ As some rivers appear to be part of the "Water", rather than "Wetlands" category in the selected terrain dataset (30), this category was given the same multiplier as the wetlands category. If we find transmission lines cross open bodies of water like Lake Tahoe, then this handling would need to be revised.

*** One order of magnitude was applied due to this text in the report, "In terms of mitigation costs, the study concentrates on a handful of line-item mitigation costs. These capture only a portion of the overall environmental costs of a project. The list of measures included in Attachment A were not intended to include costs associated with avoidance and minimization, which WECC's "capital-cost" modeling tool is assumed to capture. The study, therefore, does not factor in project costs associated with routing alternatives, specialized construction techniques, or the permitting phase—all of which may result in costs that are an order of magnitude greater than those represented in the study." (5, p. 43)

††† For possible edge cases, we have used a 1.2x multiplier instead making these fully off-limits in costing. This arises from the expedient choice to process at 250m rather than higher resolution, which means the path costing/mapping may occasionally incorrectly choose a Cat 1 cell rather than the adjacent intended non-Cat 1 cell. In order to allow this line to complete during the costing phase, the Cat 1 cell needs a multiplier assigned to it.

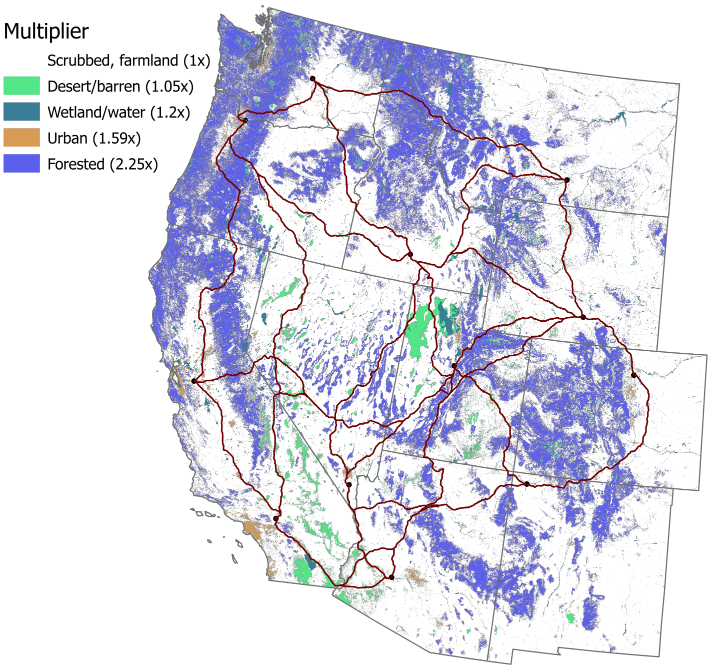

Fig. S7. Least cost path model results showing selected cost surface mutlipliers and new 500 kV transmission lines.

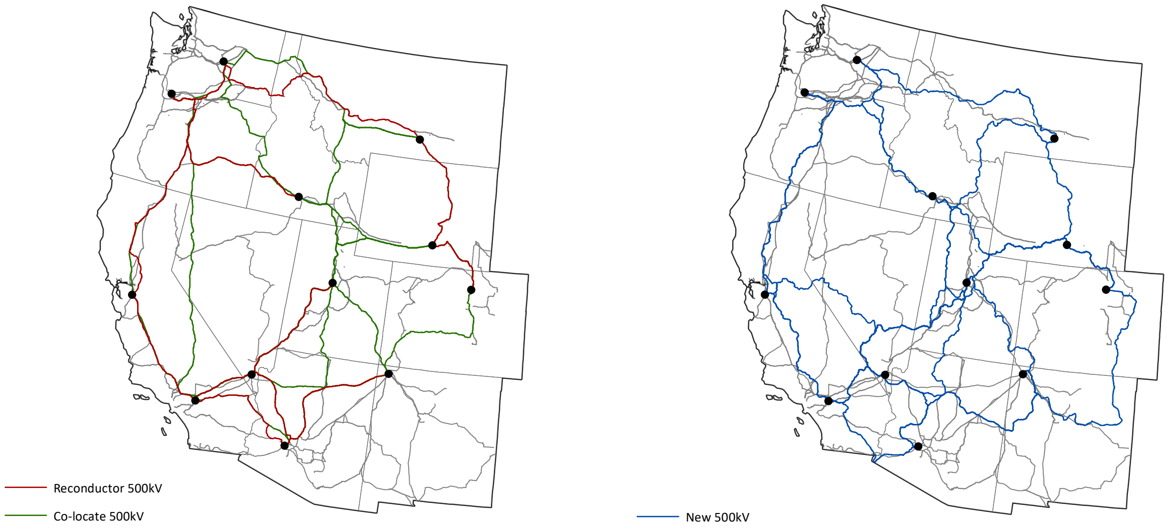

Fig. S8. Least cost path model results showing 500 kV transmission lines. Left: reconductored and co-located 500 kV lines only. Right: new 500 kV lines only.)

C.3. Spur line modeling. We used a combination of transmission costing tools, environmental conservation and risk data, GIS tools and expert input to generate spur lines for each candidate project area (CPA) in the analysis. Figure S9 details the steps to generating transmission corridors.

Choose spur line characteristics Spur lines connect CPAs to existing transmission features. We chose to connect spur lines to the existing transmission network at the geographic location of the closest existing substations. We did not determine whether there was unused capacity at each selected substation, but rather chose to build a new substation for each CPA connection to ensure integration of new renewables meeting a threshold of 500MW. An additional assumption was made that land could be found to site the substation in the vicinity of an existing substation (recent experience in the western USA suggests that land restrictions, congestion and space limitations at existing substations lead to grid-tie connection point at alternate locations where new substations are constructed).

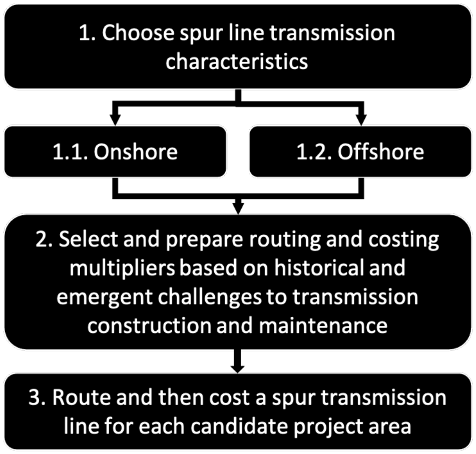

Fig. S9. spur line modelling flow

Onshore We selected the spur line characteristics shown in Table S8 to parameterize the Black & Veatch (B&V) transmission and substation cost calculators for onshore spur lines (29) (23).

Table S8. Spur line parameters for line and substation cost estimates

Parameter	Setting
Voltage class	230 kV
Number of circuits	single
Conductor type	ACSR
Tower structure	lattice (adjusted later to pole using GIS population density layer (33))
Line length	$>$ 10 miles (expedient simplification)
Build type	new
Right-of-way (ROW) width	125 feet
Include land costs for ROW	yes
AFUDC/Overhead costs	17.5% (implemented as a GIS multiplier layer)
Substation handling	one substation for all lines, plus one additional substation for every 161 km after first 161 km
Circuit breaker type	breaker and a half
Number of Line/XFMR positions	2
HVDC Converter	no
Transformer type	115/230 kV
MVA rating per transformer	200
Number of transformers	1
SVC MVAR rating	1
Shunt reactor MVAR rating	1
Series capacitor MVAR rating	1

Parameterizion of the Black & Veatch (B&V) transmission and substation cost calculators with the settings in Table S8, leads to base spur line costs of 572,843 USD2018 per km, and base substation costs of 7,609,776 for a spur line serving a CPA with a nameplate capacity of less than 200 MW, and having a spur length of less than 161 km. For CPA's with capacities greater than 200 MW, one additional transformer was added for each additional 200 MW of capacity. For CPA's with capacities greater than 400 MW, but less than 800 MW, the base spur line cost was multiplied by 1.6 to reflect the use of a double rather than a single circuit 230 kV transmission line, and two additional line/XFMR positions were added in the substation calculator. Table S9 summarizes the multipliers applied to base spur line, substation and right-of-way (ROW) costs for CPAs with capacities greater than 200 MW.
ROW land lease costs are added to each CPA's spur line following the same process described in the transmission methods section.

Table S9. Multipliers applied to base spur line and substation costs for CPAs with capacities greater than 200 MW

Parameter	200 to 400 MW	400 to 600 MW	600 to 800 MW
Spur line cost multiplier	1	1.6	1.6
Substation cost multiplier	1.24	2.22	2.46
ROW width/cost multiplier	1	1.2	1.2

Offshore After exploring recent studies on offshore wind in California (39) and New York (40), we selected the National Renewable Energy Lab's Annual technology Baseline from 2020 (ATB2020) (41) as the main source for offshore wind spur line costs. In light of known gaps in the documentation on transmission for offshore wind projects that accompanies the ATB2020, we chose a simple approach to the costing of spur lines serving offshore wind CPAs. Under this approach, we ran a linear regression on a plot of the ATB2020 offshore wind transmission costs per kW against the ATB2020's corresponding average transmission distance (see Fig S10). After determining the distance of each CPA's offshore wind transmission run in a later step, the equation arising from the linear regression will be used to determine its cost.

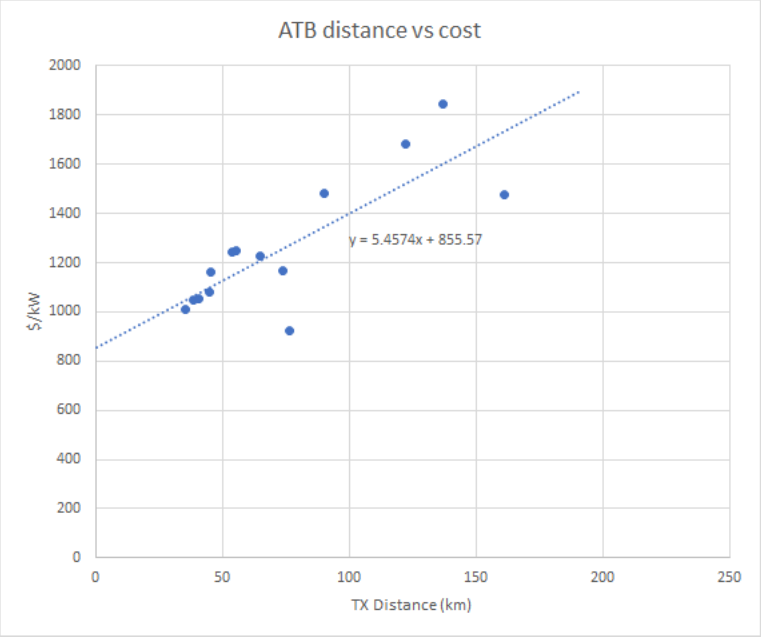

Fig. S10. Linear regression on plot of the ATB2020's offshore wind transmission costs per kW against the ATB2020's corresponding average transmission distance

The key assumption underlying this method based solely on the ATB2020 and final routed transmission distance is that the offshore wind transmission costs reported in the ATB2020 include all onshore transmission costs plus the costs of all relevant AC substations and DC converters.

Select and prepare routing and costing multipliers (spur lines) The routing and costing multipliers that were selected for use in this study are listed in Table S7 in the transmission methods section.

Route and cost spur lines

Following the same method as with the high voltage transmission lines, spur line routing was implemented by combining parameterized spur line costs with routing multipliers in order to generate a routing surface (Base cost * all routing multipliers but AFUDC/overhead + ROW land cost) * AFUDC/overhead multiplier), and then using the ArcGIS least cost path as polyline tool (38) to determine the route of each spur line to the nearest substation having a voltage rating equal to 230 kV (115 or 230 kV for offshore).

The cost of each onshore spur line option was then determined by repeating the prior processing using costing multipliers instead of routing multipliers (Base cost * all costing multipliers but AFUDC/overhead + ROW land cost) * AFUDC/overhead multiplier) and constraining spur lines to the pre-routed options determined in the last step. Sub/converter station costs were added to the cost of each new onshore spur line using the parameters shown in Tables S8 and S9.

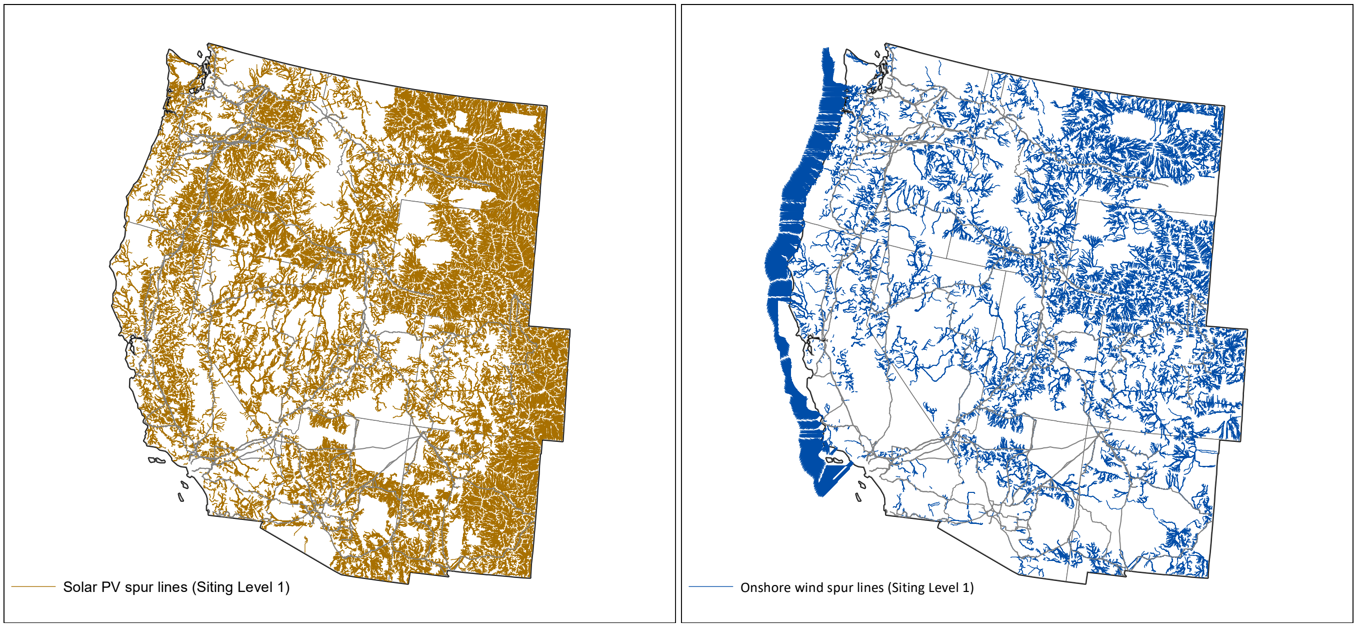

Fig. S11. Least cost path model results showing wind and solar interconnection spur lines.)

D. Step 4. Energy modeling. Two models, EnergyPATHWAYS (EP) and RIO, were used to develop the energy portfolios for the different scenarios, and to analyze the effects on portfolios and costs of the different Siting Levels. The energy modeling methodology is consistent with that used in Williams et al. (42). EP is a detailed stock-rollover accounting model that tracks infrastructure stocks, energy demand by type, and cost in every year for all energy-consuming technologies, as new stocks replace old stocks over time (for example, battery electric vehicles replacing internal combustion engine vehicles). EP divides the demand for energy services into 64 different subsectors with service demand forecasts taken primarily from the Energy Information Agency's (IEA) 2019 Annual Energy Outlook (AEO). Near-term adjustment factors based on 2020 IEA data were used to approximate the impacts of the COVID-19 pandemic. Time-varying electricity and fuel demand outputs from EP were then input into RIO, a linear programming model that combines capacity expansion with sequential hourly operations over a sampling of representative days to find the lowest-cost solution for decarbonized energy supply. Both EP and RIO were run using 12 geographic zones representing each of the western states plus a zone representing the rest of the U.S., which served as a boundary condition in the analysis.

The energy system modeling included multiple scenarios that reached net-zero greenhouse gas emissions economy-wide in the 11 western states, plus a reference scenario based on the AEO, and two scenarios that reached net-zero in the electricity sector only. The economy-wide net-zero scenarios were designed to represent different net-zero policy choices or technology uncertainties, and to study how each of these interacted with different levels of ecosystem protections (Table 1). We developed net-zero targets for the Western states energy system using boundary conditions for ecosystem carbon estimates and EPA non-CO2 supply curves. Non-energy carbon emissions and energy carbon emissions are modeled separately. We ran the Land Use and Carbon Scenario Simulator (LUCAS) model (43) to estimate the net emissions from the land sector (including all land cover change but excluding activities within the agricultural sector) with and without expanded reforestation efforts. Reforestation resulted in 33 Mt of carbon sequestration by 2050. The EPA supply curves for non-CO2 emissions mitigation are used to assume a certain level of non-CO_2 emissions reduction at <\$1000/tonne (Table S19). With this emissions accounting (Table S19), a total of 50 Mt in offsetting emissions were left unspecified to reach net-zero within the western US. However, the modeling approach achieves net zero emissions nationally by 2050, which is most consistent with political targets. If the Western region was to reach net-zero emissions within its geographic boundaries, some combination of additional negative emissions (DAC, BECCS), greater mitigation of non-CO2 emissions, or further enhancement of the land sink would be necessary. In this case, because most of the land-sink is in the Eastern U.S. while the western U.S. has a disproportionate share of non-CO2 emissions, sharing of carbon targets across the entire U.S. would allow achievement of net-zero emissions. Thus, while the western states are 50 Mt shy of net-zero in the energy modeling, the level of mitigation is still consistent with reaching net-zero at a national level assuming proportional allocation of sinks and sources. This outcome is more efficient than assuming an additional 50 Mt of direct air capture in the Western states, which was the modeling alternative.

On the demand-side, the key scenario dimension tested was the rate of electrification. The High Electrification case assumed that a 100% sales share of key electrification technologies in transportation, buildings, and industry was achieved by 2040 while the Slow Electrification case delayed the achievement of this saturation point by 20 years, to 2060. Previous studies have emphasized the importance of rapid electrification to reaching net-zero (42), so the high electrification assumption was common across the remaining scenarios. These scenarios differed on supply-side assumptions, which tested the effect of changing

renewable generation shares (cost-optimized in High Electrification case, constrained in Limited Wind and Solar, and used exclusively in Renewables Only). Other supply-side variations included greater or lesser resource sharing between states, and greater or lesser use of purpose-grown bioenergy crops (Table 1). Key RIO outputs include differences in energy portfolios and net cost of alternative scenarios compared to the High Electrification case.

D.1. Creating supply curves from candidate project areas. In the Stage 2 methodology above, the process of creating candidate project areas (CPAs) was outlined. To reduce the number of decision variables in the capacity expansion problem, we cluster the CPAs for each resource type based on capacity factor and transmission interconnection cost. Together these dimensions impact a project's levelized cost of energy (LCOE) and likelihood of inclusion in the least-cost energy system. Clustering by both capacity factor and interconnection cost gives better performance than clustering on starting LCOE alone because different rates of technological learning along the cost and performance dimensions (e.g., wind capacity factor) will otherwise cause LCOE to drift apart in future years.

A k-means squared clustering algorithm was selected because it makes it possible to select the number of bins and because it keeps variance within each cluster fairly constant. The number of bins selected within each technology type was based on expert judgement as well as some iteration within the capacity expansion modeling. A visualization of the binning for onshore wind and solar PV can be seen in Fig. S12. Each dot represents a different CPA (the size of each CPA varies and is not visualized) and the colors represent the assigned bin. Wind and solar cost and performance inputs into the capacity expansion modeling are the weighted average of all the CPAs in a bin and the total potential the sum of all the CPAs. Bin cost and performance definitions were kept consistent between siting levels but with a different total potential. This is most easily visualized in bin 4 for onshore wind where the number of CPAs is clearly reduced moving from Siting Level 1 to Siting Level 3.

From Fig. S12 the impact of pre-filtering CPAs based on an initial LCOE screen can also be seen. The purpose of these screens was to remove projects that would not get chosen in a least-cost solution while allowing the remaining bins to be grouped more tightly, reducing error from the binning process as a whole. More stringent LCOE criteria were applied to solar than wind because the shear number of available solar CPAs meant it was not necessary to entertain projects with higher cost and still have plenty of projects available. For example, Siting Level 3 still has 4.5 TW of solar potential available while excluding any project with an interconnection cost exceeding $375/kW. The capacity expansion modeling in this scenario chose a maximum of 563 GW from this supply curve. As a result of the plentitude of solar, and as is typical in this type of exercise [See NREL Annual Technology Baseline], the number of bins selected for wind technologies (8 for onshore, 6 for offshore) exceed that for solar (5 total).

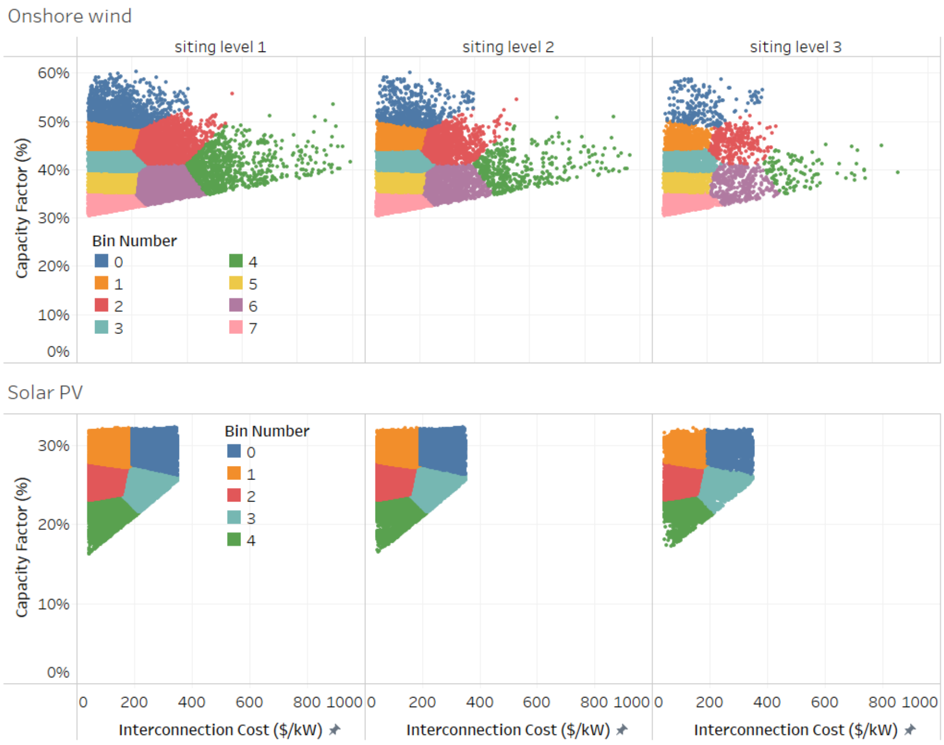

Fig. S12. k-means clustering results showing the bins for each technology and Siting Level.

D.2. Scenarios. The analysis combines six net zero energy cases with three levels of environmental siting constraints to create a total of 12 net zero economy-wide scenarios. For comparison, we included two electricity-only cases that only meets a net

zero target within the electricity sector coupled with SL1 and one reference case coupled with SL1 that does not have any emissions targets. These siting constraints were coupled with different biomass availability constraints, based on the amount of land allotted for purpose-grown biomass (Table S10), and applied to onshore wind, offshore wind, large-scale solar, interstate transmission, and spur lines that interconnect wind and solar projects to transmission.

E. Stage 5. Downscaling and land use estimates. In this stage, we downscaled RIO portfolios for onshore wind, offshore wind, utility-scale solar, urban infill solar, transmission lines, and spur lines. As a key output of the RIO model, each portfolio's annual energy generation by technology is reported at the state level (and by resource class bin for certain technologies). The goal of downscaling is to model the physical build-out of these portfolios by identifying or selecting specific locations where infrastructure projects could or are likely to be sited. The RIO model selected an amount of generation from each state. This spatial downscaling step is necessary because land and ocean use impacts vary by location, and historically, developers choose sites based on multiple criteria, making some sites more likely to be developed than others. Modeling the possible spatially-specific build-out enables us to then assess some of the environmental impacts and social implications of each portfolio (Stage 6), thus enabling us to compare portfolios by impacts in addition to their costs.

E.1. Onshore wind and utility-scale solar. We downscaled onshore wind and utility-scale solar using the following three approaches: total levelized cost of electricity (generation and transmission), random forest regression, and logistic regression. The random forest and logistic regression approaches integrate more factors into the downscaling process than levelized cost alone by extrapolating historic siting trends based on multiple factors, including locations of existing wind and solar farms and siting criteria such as distance to the nearest substation, capacity factor, and land value.

Random forest and logistic regression Random forest and logistic regression are both classification approaches. Random forest is considered a machine learning approach based on decision trees and is primarily used for prediction. Logistic regression is a standard statistical approach for binary classification and is often used for understanding explanatory variables. We used these two approaches for creating a prediction or probability map for future onshore wind and solar farms based on existing wind and solar farm locations in the 11 western states and various explanatory variables (Table S11). Both methods require a response variable that captures where solar or wind power plants do and do not exist—otherwise known generally as presence and absence locations. We generated these pseudo-absence "background" locations by randomly sampling points from within the CPAs for Siting Level 1 (after removing the footprints of existing wind or solar farms), which represent locations where wind or solar farms could be sited but are not currently sited.

Because some siting criteria (explanatory variables)—substation, transmission lines, roads, and population—vary over time, we limited our analysis to existing power plants built in or after 2018 within the 11 western states and used explanatory variable datasets representing the year 2017 (Table S11). Because solar farms are represented as point locations, we first buffered each point using a radius that would result in a land use factor of 30 MW/km^2. We also limited solar farms to those with installed capacities greater than or equal to 20 MW as a rough indicator of utility-scale, ground mounted, and grid-connected. For wind, point locations represent individual turbines and an attribute indicates turbines that form a particular power plant. We buffered each turbine using a 1.2 km radius and dissolved polygons by power plant name or EIA plant code. For turbines missing plant names or EIA codes, we grouped turbines into power plants based on spacing between turbines greater than 2 km.

We ran multiple regressions and selected the covariates in Table S12 based on the best logistic regression model with minimal degree of collinearity (using the lowest AIC score). We then generated prediction scores for each grid cell in the WECC study area. We spatially averaged the prediction scores within each CPA for each technology. To select CPAs that meet RIO portfolio results for each scenario and year, we sorted CPAs by prediction score (highest to lowest) within each resource bin for each state, calculated the cumulative generation, and selected the CPAs with cumulative generation sufficient to meet the generation target for each RIO portfolio in a given year.

Table S11. Explanatory variables in random forest and logistic regression

Variable	Source	Notes
Environmental score	See Tables S17 - S19	Environmental exclusion categories 1-3 were given scores of 3-1, respectively and combined into a single raster
BLM land value	Black and Veatch transmission land value	
Roads	Census TigerLine 2017	Used only secondary roads
Slope	See Table S2	
Population density	LandScan 2017; See Table S2	
Substations	Ventyx - 2017	selected substations with maximum voltage >= 120 kV
Transmission lines	Ventyx - 2017	selected lines with voltage >= 120 kV
Capacity factor	See section B.1	
Renewable Portfolio Standard or target	https://www.ncsl.org/research/energy/renewable-portfolio-standards.aspx	Selected the earliest target
Existing wind farm locations	US Wind Turbine Database	Selected only turbines built in or after 2018 and with total wind farm installed capacities >= 5 MW (or unknown capacities)
Existing solar farm locations	EIA	Selected only turbines built in or after 2018 and with rated capacities >= 20 MW

Table S10. Descriptions of cases, environmental Siting Levels, and scenarios

Descriptions of Cases and Environmental Siting Levels			Scenarios: combinations of Cases and Siting Levels		
	Name	Description	SL1	SL2	SL3
**Economy-wide Cases**	High Electrification	Demand: high energy efficiency, 100% sales of electric building technologies by 2040, 100% ZEV sales by 2040, fuel switching for some process heat and other fuel use, Direct Reduced Iron-making (DRI), which uses hydrogen and electricity instead of coal, carbon capture on cement<br>Supply: all generation technologies allowed	HE-1	HE-2	HE-3
	Renewables Only	Demand: Same as high electrification<br>Supply: no fossil fuel or nuclear usage by 2050	RO-1	RO-2	RO-3
	Slow Electrification	Demand: high energy efficiency, 100% sales of electric building technologies by 2060, 100% ZEV sales by 2060, 20-year delay in fuel switching for process heat, other fuel use, and DRI in iron and steel. Carbon capture on cement<br>Supply: all generation technologies allowed	SE-1	SE-2	SE-3
	Limited Biomass	Demand: Same as high electrification<br>Supply: no purpose grown biomass by 2050			LB-3
	Limited Wind and Solar	Demand: Same as high electrification<br>Supply: Annual onshore wind and large-scale solar build limited to peak historic rates in the western states (1.5 GW/year for wind; 3 GW/year for solar)			LR-3
	In-State	Demand: Same as high electrification<br>Supply: Lower reliability value placed on imports, no new long-distance transmission lines, higher transmission wheeling charges			IS-3
**Comparison cases**	Electricity Only (HE)	Demand: high electrification case electricity demand (demand for fuels is outside of system boundaries)<br>Supply: Electricity system only, net-zero emissions constraint in 2050			EOHE-3
	Electricity Only	Demand: reference electricity demand (demand for fuels is outside of system boundaries)<br>Supply: Electricity system only, net-zero emissions constraint in 2050			EO-3
	Reference	Demand: existing energy efficiency, low electrification of buildings, 10% EV adoption, no industry electrification<br>Supply: all generation technologies allowed	REF-1		
**Environ-mental Siting Levels**	Siting Level 1 (SL1)	Wind and solar: Exclude legally protected areas (Category 1; eg., national parks, wilderness areas, wildlife refuges, conservation easements)<br>Biomass: all feedstocks			
	Siting Level 2 (SL2)	Wind and solar: Exclude administratively protected areas (Category 2; e.g., critical habitat for threatened and endangered species, wetlands, areas of critical environmental concern) and Category 1.<br>Biomass: No net expansion of land for purpose-grown herbaceous biomass crops. Land for purpose-grown biomass is restricted to land that is currently used to grow bioenergy feedstocks. Specifically, land available for herbaceous biomass crops (miscanthus and switchgrass) is limited to the share of land currently cultivated for corn that is eventually consumed as corn ethanol, which is phased out in all net zero scenarios by 2050.			
	Siting Level 3 (SL3)	Wind and solar: Exclude areas with High Conservation Value (Category 3; e.g., priority and crucial habitat, intact grasslands, prime farmlands), Category 2 and Category 3.<br>Biomass: Same as Siting Level 2.			

Table S12. Logistic regression results

	Dependent variable:	
	Solar	Onshore wind
Environmental score	-0.586*	-1.502***
	(0.349)	(0.535)
BLM land rental rates	0.0004	0.001
	(0.0004)	(0.001)
Distance to nearest road	-0.0001**	-0.0001
	(0.0001)	(0.00005)
Slope	-0.454***	-0.041
	(0.135)	(0.074)
Population density	0.007	0.121
	(0.022)	(0.173)
Capacity factor solar	0.318***	
	(0.109)	
Capacity factor wind		33.192***
		(6.161)
Distance to nearest transmission line	-0.00001	-0.0001***
	(0.00002)	(0.00003)
Distance to nearest substation	-0.0001***	0.00001
	(0.00002)	(0.00002)
RPS target	0.452	3.485
	(1.934)	(2.251)
Constant	-5.787**	-11.806***
	(2.700)	(2.506)
Observations	223	147
Akaike Inf. Crit.	128.153	115.672
Note:	*p<0.1; **p<0.05; ***p<0.01	

Estimation of LCOE This section describes the Levelized Cost of Energy (LCOE) calculation. LCOE is calculated for each CPA (Masters 2004). The calculation is done on a matrix basis, to incorporate location-specific annual energy production and capital cost for each CPA).

$$LCOE = annualPayments/annualGeneration$$

Where:

$AnnualPayments = ProjectCapitalCost \times CRF(0.04, 20)$

CRF, or annual capital recovery factor, is a function of interest rate (assumed here to be 4%) and loan term (assumed here to be 20 years, resulting in CRF = 0.0736)

$LCOE = (ProjectCapitalCost \times CRF)/AnnualGeneration$

Attributes for each CPA are calculated as follows:

$CPACapitalCost = GenerationCapitalCost + TransmissionCapitalCost$

$GenerationCapitalCost(\$) = NameplateCapacity$ (MW) $\times GenericTechnologyCapitalCost$ (\$/MW, varies by region)

Transmission capital cost for each CPA is determined based on least-cost-path identification for interconnection spur lines to the nearest substation with voltage 230 kV or above. Spur line cost includes both line and substation cost.

$AnnualGeneration$ (MWh) $= NameplateCapacity \times 8760$ (hrs/yr) $\times CapacityFactor$ (%)

$$NameplateCapacity \text{ (MW ac)} = ShapeArea \text{ (km}^2\text{)} \times PowerDensity \text{ (MW ac/km}^2\text{, varies by technology)}$$

$CapacityFactor$ was estimated previously, as described in Stage 2: Renewable Resource Assessment.

In the LCOE-based approach, the selected portfolio is assigned to specific locations in each zone, incrementally up to the needed amount (annual MWh) to satisfy the portfolio, in order of increasing LCOE. However, we determined that results were similar enough between logistic regression and random forest, and that these two approaches were improvements upon the LCOE-based approach to warrant only reporting results using the random forest method.

E.2. Offshore wind. For offshore wind, sites were selected based on total levelized cost, since there are no existing offshore wind farms along the western US coastline. The LCOE-based approach for downscaling offshore wind is similar to that for onshore wind and utility-scale solar; however for offshore wind, siting is prioritized in BOEM-designated offshore wind leasing and planning areas.

E.3. Power lines. Spur lines are selected corresponding to the portfolio's selected CPAs (utility-scale solar, onshore and offshore wind). High-voltage transmission line features are selected corresponding to the portfolio's transmission upgrade specifications. High-voltage interzonal transmission lines are displayed as discrete line segments, and routes are identified based on the least-cost path analysis described in previous sections. Lines were selected based on applying minimum build thresholds to the capacities selected by RIO in each scenario (Table SS13). Routes shown in map figures are not intended to be prescriptive, but indicative of the potential magnitude of transmission network upgrades that could be needed to accommodate the level of west-wide renewable resource-sharing included in the portfolio.

Table S13. Minimum capacity (MW) thresholds for downscaling transmission

	Reconductor	Co-locate	New line
230 kV	50	NA	NA
345 kV	100	NA	NA
500 kV AC	150	250	250
500 kV DC	150	250	250

E.4. Land use estimates for other technologies. We did not downscale other infrastructure projects (energy generation or carbon sources)—geothermal, nuclear, natural gas, hydropower, direct air capture (DAC)—either due to the limited land area requirements (e..g, nuclear, DAC, geothermal) or because there is little to no expansion of the technology across the majority of the core scenarios (e.g., natural gas, hydropower). Biomass land area requirements for certain feedstocks can be significant, but since nearly all purpose grown biomass resources are sourced from the Midwest and south—outside of the states in the Western interconnection, downscaling biomass resources would largely be outside of the geographic scope and thus scope of this study. Instead, for other infrastructure projects, we report just the land area requirements using an average land use factor (e.g., km^2/MWh or km^2/MW) sourced from the literature. For biomass, RIO results are reported as dry tons of woody, waste, or herbaceous biomass resource form categories by price bin and by state. In order to estimate biomass land use requirements, we disaggregated biomass demand by specific resource with each resource form category using proportional allocation based on the supply curve in The Billion Ton study (19). For example, within the herbaceous resource form category and price point of \$100 for the state of Colorado in 2040, the resource supply is roughly 42% wheat straw, 16% switchgrass, 41% corn stover, and <1% barley straw. We allocated estimated demand for each of these herbaceous resources by multiplying Colorado's total herbaceous demand in 2040 by each of these fractions. We considered no dedicated land use requirements for residue and waste resources. For woody resources, we estimated land use using yields for eucalyptus, hardwood upland and lowland whole trees, mixed wood whole trees, pine, popular, willow, and softwood natural and planted whole trees. For herbaceous resources, we estimated land use needs for switchgrass and miscanthus, biomass sorghum, and energy cane.

Table S14. Land use factor assumptions

Technology or resource	Source	land use efficiency	Units
Onshore wind	Miller and Keith (2018, 2019) (14, 15)	2.7	MW/km^2
Offshore wind	Borrmann et al. (2018) (16) median of the literature values	5.2	MW/km^2
Utility-scale solar	Ong et al. (2013) (44), Hernandez et al. (2015) (45)	30	MW/km^2
Transmission	WECC 2019 Transmission Capital Cost Tool (46)	230 kV: 40 m; 345 kV: 60 m; 500 kV: 76 m	meters (corridor width)
Direct Air Capture	Baker et al. 2020 (47)	2	km^2/MT (million tonnes)
Switchgrass	http://switchgrass.okstate.edu/realistic-expectations-for-switchgrass 6 tons/acre	15	dry tons/ha (US tons)
Miscanthus	https://www.nrcs.usda.gov/Internet/FSE_DOCUMENTS/stelprdb104 4768.pdf 10 tons/acre	25	dry tons/ha (US tons)
Biomass sorghum	Rooney et al. 2007 (48) (18-32 or 25 Mg/ha)	25	dry tonnes/ha

Energy cane	Salassi et al. (2014) (49)	16.4	dry tons/ha (US tons)
Eucalyptus	Hall et al. (2020) (50)	50	dry tons/ha (US tons)
Hardwood	Use the same assumptions as willow	11.7	dry tons/ha (US tons)
Mixed wood	Average between hardwood and softwood	15.5	dry tons/ha (US tons)
Pine	https://www.warnell.uga.edu/sites/default/files/publications/SRWB_Growth_and_Yield_Paper_6_July_2011_0.pdf 10 green tons/acre/year, 22% water volume (https://shodor.org/succeedhi/succeedhi/weightree/percentWater-content.html) (assuming 9 year rotation)	19.2	dry tons/ha (US tons)
Poplar	Langholtz et al. 2016 (19) average of range from 3.9 to 5.6 dry tons per acre	11.7	dry tons/ha (US tons)
Willow	Langholtz et al. 2016 (19) average of range from 2.8 to 6.2 dry tons per acre	11.7	dry tons/ha (US tons)
Softwood	Use the same assumptions as pine	19.2	dry tons/ha (US tons)

F. Stage 6. Strategic environmental assessment. We conducted an area-based strategic environmental assessment using the modeled generation, gen-tie, and transmission spatial build-out of portfolios created in Stage 5 (Section E). Using spatial overlay functions in Python, we estimated the area of each land cover type or environmental metric impacted by wind, solar, or power line development.

The purpose of the strategic environmental assessment is to anticipate the impact of energy development on lands with conservation value, and to examine whether siting protections can be effective in reducing development in areas with high conservation value. For transmission lines with polyline spatial data, we approximated polygon corridor footprints using the average corridor width for each line based on voltage-specific Right of Way factors in the WECC TEPPC transmission cost calculator (see SM Table S14 for widths). For each infrastructure type (generation, gen-tie, transmission) and each scenario, we calculated the amount of land area that overlaps with the three Environmental Exclusion Categories, 20 other environmental metrics, and social metrics such as population density and demographics. Ecological and landscape metrics included critical habitat for sensitive and listed species, sage grouse habitat, Important Bird Areas, wetlands, big game corridors, eagle habitat, wildlife linkages (51), wildfire risk, and habitat areas of particular concern (for offshore wind). Working lands metrics include all agricultural land (crop and pasture land), prime farmland, and rangelands (52). For rangelands, we used the only known publicly available rangelands extent maps for the U.S. created by Reeves and Mitchell (52) and chose the map created using the National Resources Inventory (NRI) definition of rangelands mapped using the 2001 LANDFIRE landcover dataset. We use the rangelands definition adopted by the Natural Resources Conservation Service's NRI program, which states that rangelands are, "land on which the climax or potential plant cover is composed principally of native grasses, grass-like plants, forbs or shrubs suitable for grazing and browsing, and introduced forage species that are managed like rangeland" (52). Several environmental metrics are comprised of datasets that are also used in Environmental Exclusion Categories 2-3. See SM Table S16 for the underlying datasets, sources for each metric, and whether a metric was also included in an Environmental Exclusion Category.

The metrics for the strategic environmental assessment were chosen to represent two types of impacts—specific and generalized. The specific metrics (e.g., sage grouse habitat and wildlife linkages) were intended to explore areas of focus in current public discourse in energy planning forums. Thus, several specific metrics were chosen to explore trends and implications to key species. In contrast, the generalized metrics (e.g., impacts to Environmental Exclusion Category 3 lands) are meant to explore overall impacts to natural and working lands for a given resource portfolio.

For social metrics, we calculated the area-weighted average median income, percent living below poverty, percent unemployed, and population density for each infrastructure type using the social vulnerability metrics collected by the Census Bureau and made available by the CDC and ATSDR (53). Finally, we estimated the human population residing within several buffered distances of each downscaled infrastructure type as an indicator of the number of people within a given proximity to this infrastructure using the LandScan population density dataset (54). Studies of wind farm visibility suggest that turbines have visual impact within a distance of 16 km from a wind farm (55), but no equivalent distances have been determined for solar PV farms (56, 57). For high voltage transmission lines, studies have suggested 2-4 km as the distance within which a 500 kV transmission tower has high visual impact (58). The distance is slightly shorter (1.6 km) for spur transmission lines rated at 230 kV (58)

Table S15. Existing and planned energy infrastructure datasets

Broad category	Dataset name	Source	Website	Description	Data type/ resolution	Usage in study
Existing power plant locations	United States Wind Turbine Database (USWTDB)	USGS, Berkeley Lab, AWEA	https://eerscmap.usgs.gov/uswtdb/data/	Point locations of on-shore and off-shore turbines in the U.S. It is updated quarterly. Accessed on 9/13/18	Shapefile or Geojson	Exclude from potential project areas
Existing power plant locations	Surface area of solar arrays in the conterminous United States as of 2015	USGS (21)	https://www.sciencebase.gov/catalog/item/57a25271e4b006cb45553efa	Footprint area of solar arrays in the conterminous U.S. based on EIA utility-scale facilities data from 2015	Shapefile	Exclude from potential project areas
Existing power plant locations	Surface area of utility-scale solar arrays in California as of 2018	The Nature Conservancy (59)	Unpublished	Footprint area of solar arrays in California created using satellite imagery	Shapefile	Exclude from potential project areas
Existing power plant locations	California's commercial wind and solar project locations	DataBasin, Black & Veatch, Public Utilities Commission	https://databasin.org/maps/365216c4ead144718ec68294035a2646	Existing and commercial wind and solar project locations (those with power purchase agreements from RPS Calculator and the California Public Utilities Commission)	Shapefile (point locations)	Used in conjunction with footprint areas to exclude from potential project areas
Existing power plant locations	Renewable Portfolio Standard Executed Projects (California)	Public Utilities Commission	http://cpuc.ca.gov/RPS_Reports_Data/	Public information of investor owned utility renewable contracts under the RPS program include: contract summaries, contract counterparties, resource type, location, delivery point, expected deliveries, capacity, length of contract, and online date.	Spreadsheet with geographic coordinates of project locations	Used in conjunction with footprint areas to exclude from potential project areas
Transmission infrastructure	Electric transmission line	Homeland Infrastructure Foundation Level Data (HIFLD)	https://hifld-geoplatform.opendata.arcgis.com/datasets/electric-power-transmission-lines	Transmission line locations as polylines with attribute data on voltages. This data are usually updated quarterly. Accessed on 6/23/2020.	Geodatabase feature class	Selecting potential project areas and modeling transmission corridor needs.
Transmission infrastructure	BLM recently approved Transmission lines	Environmental Planning Group LLC, Bureau of Land Management, Argonne National Labs	View lines: https://bogi.evs.anl.gov/section368/portal/	We included the following six planned transmission corridors in "advanced development" and "recently approved": Gateway South, Gateway West, Southline, SunZia, TransWest Express, SWIP North, and Boardman to Hemingway. Spatial data can be requested from Argonne National Labs. These lines are listed as being in Phase 2 or 3 of the WECC Path Rating Process in the California Energy Commission's RETI 2.0 report "RETI 2.0 Western States Outreach Project Report" (https://www.energy.ca.gov/reti/reti2/documents/)	Geodatabase feature class	Selecting potential project areas and modeling transmission corridor needs. Buffered lines using project reports' planned corridor width

Table S16. Datasets for Strategic Environmental Assessment

Metric	Dataset name	Source	Environmental Exclusion Category	Unique ID	Data type/ resolution
Critical habitat	Critical habitat		2	0051	Shapefile
Critical habitat	Desert tortoise critical habitat	WWWMP (high level)	2	0075	Shapefile
Critical habitat	Coastal critical habitat		2	0101	Shapefile
Critical habitat	Critical habitat	WWWMP (high level)	2	0262	Shapefile
Sage Grouse habitat	Priority habitat management area - exclusion	WWWMP - BLM	2	0257	Shapefile
Sage Grouse habitat	Priority habitat management area, high level siting considerations	WWWMP - BLM	2	0258	Shapefile
Sage Grouse habitat	General habitat management area, high level siting considerations	WWWMP - BLM	3	0259	Shapefile
Sage Grouse habitat	General habitat management area, moderate level siting considerations	WWWMP - BLM	3	0260	Shapefile
Sage Grouse habitat	Greater sage grouse priority areas for conservation	FWS	2	0266	Shapefile
Important Bird Areas	Important Bird Areas - state and globally important (Apr 2018)	Audubon Society	3	0110	Shapefile
Big game corridors	Wyoming Big Game Crucial Habitat (Elk, Mule Deer, Bighorn Sheep, Pronghorn, White-tailed Deer)	Wyoming Game and Fish	2	0100	Shapefile
Big game corridors	WECC Big Game (ALLTYPES3 LIKE '%Big Game Winter Range%')	WECC	3	0105	Shapefile
Big game corridors	Washington Deer areas	Washington Department of Fish and Wildlife	3	0123	Shapefile
Big game corridors	Washington Elk areas	Washington Department of Fish and Wildlife	3	0124	Shapefile
Big game corridors	Oregon Elk and Deer Winter Range	Oregon Department of Fish and Wildlife	3	0149	Shapefile
Big game corridors	Columbian White-tailed deer range	USFWS	3	0155	Shapefile
Wildlife linkages	Wildlife linkages with corridor values $>$ 34.3428	The Wilderness Society (51)	4	0172	Shapefile
Eagle habitat	Bald Eagle habitat	WWWMP - BLM	2 (wind only)	0076	Shapefile
Eagle habitat	West-wide eagle risk data using the 2 of quantile bins (top 30% of eagle habitat)	USFWS (Bedrosian et al. 2018)	2 (wind only)	0102	Shapefile
Eagle habitat	Golden Eagle habitat	WWWMP	2 (wind only)	0228	Shapefile
RCN	Resilient Connected Network	The Nature Conservancy https://maps.tnc.org/resilientland/	3	na	Shapefile
HAPC	Marine Habitat Areas of Particular Concern	NOAA	2	na	Shapefile
Prime farmland	Prime farmland based on high quality soils	Natural Resources Conservation Service	3	0267	Shapefile
Agricultural land, grassland, shrubland, conifer	LANDFIRE 2016 Existing Vegetation Type (EVT) "EVT_PHYS" field	LANDFIRE https://landfire.gov/version_download.php#	NA	NA	raster/ 30m
Rangelands	U.S.rangelands extent using NRI-LANDFIRE model	Reeves et al. 2011 (52)	NA	NA	raster/ 30m
Population density and count	LandScan Global Population Density and Count	LANDSCAN ORNL https://landscan.ornl.gov/	NA	NA	raster
Wildfire risk	Wildfire Hazard Potential	US Forest Service and US Department of Agriculture https://www.firelab.org/project/wildfire-hazard-potential	NA	NA	raster/270m
Social Vulnerability Index	CDC Social Vulnerability Index (EP_POV, EP_UNEMP, EP_PCI)	Center for Disease Control and Prevention SVI 2018 https://www.atsdr.cdc.gov/placeandhealth/svi/documentation/SVI_documentation_2018.html	NA	NA	shapefile

Table S17. Datasets for Environmental Exclusion Category 1 (site suitability). Definitions: Exclude development (EX), Avoid development (AV), WECC Environmental Risk Class (RC 1, 2, 3, 4), BLM High Level Siting Considerations (HLSC), BLM Moderate Level Siting Considerations (MLSC), Information not available (NA). Study abbreviation and names: ORB 2015 (Optimal Renewable Energy Build-out), BLM WSEP (BLM Western Solar Energy Program), BLM WWW-MP (BLM West-wide Wind Mapping Program), WECC (Western Electricity Coordinating council Environmental Data Viewer), WREZ (Western Renewable Energy Zones), RETI CPUC (Renewable Energy Transmission Initiative of the California Public Utilities Commission)

Unique Data ID	Environmental Category	Technology	Data Publisher Organization	Dataset Name	ORB 2015 (7)	BLM WSEP (60)	BLM WWW-MP (?)	BLM DRECP (8)	WECC (61)	WREZ (4)	RETI CPUC (3)
1	1	All	National Park Service	NPS boundaries - National Historic Trails	EX	EX	EX	EX	RC 2	EX	EX
2	1	All	BLM - WWWMP	National Scenic Trails	NA	EX	EX	EX	NA	EX	NA
3	1	All	BLM - WWWMP	National Historic Landmarks	NA	NA	EX	NA	NA	NA	NA
4	1	All	BLM - WWWMP	National Natural Landmarks	NA	NA	EX	NA	NA	NA	NA
5	1	All	United States Geological Survey	Wild and Scenic Rivers	NA	EX	EX	NA	RC 3	EX	EX
6	1	All	Natural Resources Conservation Service	Easements	EX	NA	NA	NA	RC 3	EX	EX
8	1	All	National Conservation Easement Database	Conservation Easements	NA	NA	NA	NA	NA	NA	NA
9	1	All	Bureau of Land Management	BLM Solar Energy Program SEZ non-dev	NA	EX	NA	NA	NA	NA	NA
10	1	All	BLM - WWWMP	Visual Resource Management	NA	EX	EX	NA	NA	AV	NA
11	1	Solar	Bureau of Land Management	BLM Solar Energy Program exclusions	NA	NA	NA	NA	NA	NA	NA
12	1	All	USGS PAD-US	National Primitive Area	EX	NA	NA	NA	RC 4	EX	NA
13	1	All	USGS PAD-US	National Wildlife Refuge	EX	NA	NA	NA	RC 4	EX	EX
14	1	All	USGS PAD-US	Units of the National Parks System (excluding National Recreation Areas and National Trails)	EX	EX	NA	NA	RC 4	EX	EX
15	1	All	USGS PAD-US	Wilderness Area	EX	EX	EX	NA	RC 4	EX	EX
16	1	All	USGS PAD-US	Wilderness Area (Recommended)	NA	NA	NA	NA	RC 4	EX	NA
17	1	All	USGS PAD-US	Wilderness Study Area	EX	EX	EX	NA	RC 4	EX	EX
20	1	All	BLM - WWWMP	National Conservation Area	EX	EX	EX	NA	RC 3	EX	EX
21	1	All	USGS PAD-US	National Monument	EX	EX	EX	NA	RC 3	EX	EX
22	1	All	USGS PAD-US	National Recreation Area	EX	NA	NA	NA	RC 3	EX	EX
23	1	All	USGS PAD-US	Research Natural Area – Proposed	EX	NA	NA	NA	RC 3	NA	NA
24	1	All	BLM - WWWMP	Desert Renewable Energy Conservation Plan Special Recreation Management Area	EX	EX	EX in CA; MLSC elsewhere.	EX	NA	AV	EX in DRECP area
25	1	All	USGS PAD-US	State Park	EX	NA	NA	NA	RC 3	EX	EX
26	1	All	USGS PAD-US	State Wildlife Management Areas	EX	NA	NA	NA	RC 3	AV	NA
28	1	All	BLM - WWWMP	National Register Historic Places		NA	EX	NA	NA	NA	NA
29	1	All	USGS PAD-US	State Wilderness Areas	EX	NA	NA	NA	NA	EX	EX
30	1	All	USGS PAD-US	DFW Wildlife Areas and Ecological Reserves	EX	NA	NA	NA	NA	NA	NA
31	1	All	USGS PAD-US	Existing Conservation and Mitigation Bank	EX	NA	NA	NA	NA	EX	EX
32	1	All	USGS PAD-US	Watershed Protection Area	EX	NA	NA	NA	NA	EX	NA
33	1	All	USGS PAD-US	Marine Protected Area	EX	NA	NA	NA	NA	EX	NA
34	1	All	USGS PAD-US	Historic or Cultural Area	EX	EX	NA	NA	NA	AV	EX
35	1	All	California State Agencies	Habitat Conservation Plan	AV	NA	NA	NA	Non-preferred dataset	AV	EX
36	1	All	California State Agencies	Natural Community Conservation Plan	AV	NA	NA	NA	Non-preferred dataset	AV	EX
38	1	All	BLM - WWWMP	DRECP NCL	NA	NA	NA	EX	NA	NA	NA
39	1	All	BLM - WWWMP	Park boundaries	NA	NA	NA	NA	NA	NA	NA
190	1	Solar	USGS PAD-US	Right of Way exclusion	NA	NA	NA	NA	RC 3	NA	NA
240	1	All	Colorado Natural Heritage Program	Colorado protected lands	NA	NA	NA	NA	NA	NA	NA
252	1	All	BLM - WWWMP	conservation	NA	NA	EX	NA	NA	NA	NA
256	1	All	BLM - WWWMP	Right of Way exclusion	NA	NA	EX	NA	NA	NA	NA
301	1	All	The Nature Conservancy	GAP_StsPNW_V2_20200326	NA	NA	NA	NA	NA	NA	NA
306	1	All	State of Montana	Montana Public Lands	NA	NA	NA	NA	NA	NA	NA
311	1	All	The Nature Conservancy Idaho	Idaho TNC Fee	NA	NA	NA	NA	NA	NA	NA
8b	1	All	The Nature Conservancy Wyoming	Conservation Easements	NA	NA	NA	NA	NA	NA	NA
8c	1	All	The Nature Conservancy Arizona	Conservation Easements	NA	NA	NA	NA	NA	NA	NA
8d	1	All	State of Montana	Montana Conservation Easements	NA	NA	NA	NA	NA	NA	NA
8e	1	All	The Nature Conservancy Idaho	Idaho Conservation Easements	NA	NA	NA	NA	NA	NA	NA
8f	1	All	The Nature Conservancy Idaho	Idaho Other Conservation Easements	NA	NA	NA	NA	NA	NA	NA

Table S18. Datasets for Environmental Exclusion Category 2. Definitions: Exclude development (EX), Avoid development (AV), WECC Environmental Risk Class (RC 1, 2, 3, 4), BLM High Level Siting Considerations (HLSC), BLM Moderate Level Siting Considerations (MLSC), Information not available (NA). Study abbreviation and names: ORB 2015 (Optimal Renewable Energy Build-out), BLM WSEP (BLM Western Solar Energy Program), BLM WWW-MP (BLM West-wide Wind Mapping Program), WECC (Western Electricity Coordinating council Environmental Data Viewer), WREZ (Western Renewable Energy Zones), RETI CPUC (Renewable Energy Transmission Initiative of the California Public Utilities Commission)

Unique Data ID	Environmental Category	Technology	Data Publisher Organization	Dataset Name	ORB 2015 (7)	BLM WSEP (60)	BLM WWW-MP (?)	BLM DRECP (8)	WECC (61)	WREZ (4)	RETI CPUC (3)
18	2	All	BLM - WWWMP	Areas of Critical Environmental Concern	EX	EX	EX	EX	RC 3	EX	EX
27	2	Wind	New Mexico County governments	New Mexico County wind ordinances	NA	NA	NA	NA	NA	NA	NA
43	2	All	BLM - WWWMP	Visual Resource Management II	NA	NA	HLSC	NA	NA	NA	NA
44	2	All	USGS PAD-US	State Forest	EX	NA	NA	NA	RC 3	EX	EX
45	2	All	BLM - WWWMP	National Park Service Areas of High Potential Resource Conflict	NA	NA	MLSC	NA	NA	NA	AV
46	2	All	California Department of Fish and Game	Central Valley Wetland and Riparian Areas	EX	NA	NA	NA	RC 3	EX	EX
47	2	All	BLM - WWWMP	No Surface Occupancy	EX	EX	HLSC	NA	NA	NA	NA
51	2	All	United States Fish and Wildlife Service	Critical Habitat for Threatened and Endangered Species Composite Layer	AV	EX	HLSC	NA	RC 3	NA	AV
51b	2	All	Montana Fish, Wildlife and Parks	Bull Trout and Pallid Sturgeon Streams and Lakes	NA	NA	NA	NA	NA	NA	NA
52	2	All	United States Fish and Wildlife Service	Wetlands - prc	EX	NA	NA	NA	RC 2	EX	NA
52b	2	All	State of Montana	Wetlands and Riparians	EX	NA	NA	NA	RC 2	EX	NA
52c	2	All	State of New Mexico	NMFAP2020$Theme_Biodiversity$RiparianCorridors	EX	NA	NA	NA	RC 2	EX	NA
53	2	All	United States Forest Service	National Inventoried Roadless Areas	EX	NA	NA	NA	RC 3	EX	EX
54	2	All	Nevada Natural Heritage Program	Priority Wetlands Inventory	NA	NA	NA	NA	RC 2	NA	NA
57	2	All	USGS PAD-US	Special Interest Area	AV	NA	NA	NA	RC 3	AV	NA
58	2	All	BLM - WWWMP	Desert Renewable Energy Conservation Plan Extensive Recreation Management Area	NA	NA	HLSC	AV	NA	NA	NA
59	2	All	BLM - WWWMP	Desert Renewable Energy Conservation Plan Wildlife Allocation	NA	NA	EX	AV	NA	NA	EX in DRECP
60	2	All	BLM - WWWMP	Desert Renewable Energy Conservation Plan Off Highway Vehicles	NA	NA	EX	EX	NA	AV	NA
61	2	All	BLM - WWWMP	Off Highway Vehicle	NA	NA	MLSC	NA	NA	AV	NA
62	2	Wind	BLM - WWWMP	Development Focus Area - solar and geothermal only (excluding wind)	NA	NA	EX	Prioritize (varies by technology)	NA	NA	NA, except in SJV/ DRECP screen
63	2	All	USGS PAD-US	U.S. Army Corps of Engineers Land	NA	NA	NA	NA	RC 2	NA	NA
65	2	All	USGS PAD-US	Other private non-profit land	EX	NA	NA	NA	RC 2	NA	NA
66	2	All	TNC WAFO	TNC_Lands_Features	EX	NA	NA	NA	NA	NA	EX
67	2	All	WA DNR	Spotted Owl Management Units	NA	NA	NA	NA	NA	EX	EX
68	2	All	WA DNR	Habitat Conservation Plan Lands	NA	NA	NA	NA	NA	EX	EX
71	2	All	USGS PAD-US	State Reserves	AV	NA	NA	NA	NA	NA	NA
72	2	All	USGS PAD-US	Other wildlife areas and ecological reserves	AV	NA	NA	NA	NA	NA	NA
73	2	All	Los Angeles County	Significant ecological areas	AV	NA	NA	NA	NA	NA	AV
75	2	All	BLM - WWWMP	Desert Tortoise Critical Habitat	AV	NA	HLSC	NA	NA	NA	NA
76	2	Wind	from Courtney Larson, TNC	Bald Eagle Summer and Winter High Probability of occurrence	NA	NA	MLSC	NA	NA	NA	NA
77	2	All	USFWS	Vernal pools	NA	NA	NA	NA	NA	NA	AV
78	2	All	USFWS	2012RemapVernalPoolsFINAL.zip	NA	NA	NA	NA	NA	NA	AV
79	2	All	CDFW	SANGIS_ECO_VERNAL_POOLS.shp	NA	NA	NA	NA	NA	NA	AV
80	2	All	CDFW	ds948.shp	NA	NA	NA	NA	NA	NA	AV
81	2	All	USDA Forest Service, Modoc National Forest.	Vernal pools, Modoc. ds949.zip	NA	NA	NA	NA	NA	NA	AV
82	2	All	BLM	BLM Lands with Wilderness Characteristics (DRECP)	NA	EX	EX	See CMAs	NA	AV	NA
83	2	All	BLM - WWWMP	BLM Lands with Wilderness Characteristics (WWWMP)	NA	EX	EX	See CMAs	NA	AV	NA
85	2	All	Bureau of Land Management DRECP	National Landscape Conservation Survey Preferred Subareas	NA	EX	EX	EX	NA	EX	NA
91	2	Wind	TNC "Site Wind Right" study	Cooperative Whooping Crane Tracking Project database Pearse et al. (2015) National Wetlands Inventory	NA	NA	NA	NA	NA	NA	NA
92	2	Wind	University of Kansas, Kansas Biological Survey	SGPCHAT	NA	NA	NA	NA	NA	NA	NA
96	2	All	Colorado Parks and Wildlife	Preble S Jumping Mouse	NA	NA	NA	NA	NA	NA	NA
97	2	All	Colorado Parks and Wildlife	Mule deer	NA	NA	NA	NA	NA	NA	NA

Table S18. Datasets for Environmental Exclusion Category 2. Definitions: Exclude development (EX), Avoid development (AV), WECC Environmental Risk Class (RC 1, 2, 3, 4), BLM High Level Siting Considerations (HLSC), BLM Moderate Level Siting Considerations (MLSC), Information not available (NA). Study abbreviation and names: ORB 2015 (Optimal Renewable Energy Build-out), BLM WSEP (BLM Western Solar Energy Program), BLM WWW-MP (BLM West-wide Wind Mapping Program), WECC (Western Electricity Coordinating council Environmental Data Viewer), WREZ (Western Renewable Energy Zones), RETI CPUC (Renewable Energy Transmission Initiative of the California Public Utilities Commission)

Unique Data ID	Environmental Category	Technology	Data Publisher Organization	Dataset Name	ORB 2015 (7)	BLM WSEP (60)	BLM WWW-MP (?)	BLM DRECP (8)	WECC (61)	WREZ (4)	RETI CPUC (3)
100	2	All	Wyoming Game and Fish	Big Game Crucial Habitat	NA	NA	NA	NA	RC 3	NA	NA
101	2	All	NOAA/USFWS	Critical Habitat Designations (map service layer)	AV	NA	NA	NA	NA	NA	NA
102	2	Wind	FWS	West-Wide Golden Eagle Nest Density	NA	NA	NA	NA	NA	NA	NA
185	2	All	USGS PAD-US	Research Natural Area	EX/AV	NA	NA	NA	RC 3	AV	EX
234	2	Wind	Colorado Parks and Wildlife	Bald Eagle nest sites, roosting sites, concentration areas	NA	NA	NA	NA	NA	NA	NA
239	2	Wind	Colorado Parks and Wildlife	Colorado Least Tern nesting and foraging sites	NA	NA	NA	NA	NA	NA	NA
248	2	All	Contact TNC MT chapter for more information.	Montana Wetland Areas	NA	NA	NA	NA	NA	NA	NA
249	2	All	WHSRN	Globally important wetlands	NA	NA	NA	NA	NA	NA	NA
257	2	All	BLM - WWWMP	Sage Grouse Priority Habitat Management Area exclusion	NA	NA	EX	NA	NA	NA	NA
258	2	All	BLM - WWWMP	Sage Grouse Priority Habitat Management Area, High Level Siting Requirements	NA	NA	HLSC	NA	NA	NA	NA
262	2	All	BLM - WWWMP	critical habitat	NA	NA	HLSC	NA	NA	NA	NA
263	2	All	BLM - WWWMP	Special Recreation Management Area	NA	NA	HLSC	EX	NA	NA	NA
266	2	Wind	TNC	GreaterSageGrousePACs.gdb	NA	NA	NA	NA	NA	NA	NA
271	2	Wind and solar only, geothermal is an exception	County government	Imperial County: areas outside Renewable Energy Overlay	NA	NA	NA	NA	NA	NA	NA
272	2	All	County government	Inyo County: areas outside Solar Energy Development Areas (SEDAs)	NA	NA	NA	NA	NA	NA	NA
302	2	All	The Nature Conservancy	GAP_StsPNW_V2_20200326	NA	NA	NA	NA	NA	NA	NA
307	2	All	Montana Natural Heritage Program	Montana_GrSageGrouseLeks_4mileBuffers.shp	NA	NA	NA	NA	NA	NA	NA
310	2	All	Wyoming Game and Fish	WYSageGrouseCoreAreasVer4_WGFD2015	NA	NA	NA	NA	NA	NA	NA
314	2	All	Montana Natural Heritage	Montana Natural Heritage Program Threatened and Endangered Species	NA	NA	NA	NA	NA	NA	NA
18b	2	All	Bureau of Land Management	Areas of Critical Environmental Concern	NA	NA	NA	NA	NA	NA	NA
92b	2	Wind	Colorado Parks and Wildlife	CPW All Species Activity Mapping Data	NA	NA	NA	NA	NA	NA	NA

Table S19. Datasets for Environmental Exclusion Category 3. Definitions: Exclude development (EX), Avoid development (AV), WECC Environmental Risk Class (RC 1, 2, 3, 4), BLM High Level Siting Considerations (HLSC), BLM Moderate Level Siting Considerations (MLSC), Information not available (NA). Study abbreviation and names: ORB 2015 (Optimal Renewable Energy Build-out), BLM WSEP (BLM Western Solar Energy Program), BLM WWW-MP (BLM West-wide Wind Mapping Program), WECC (Western Electricity Coordinating council Environmental Data Viewer), WREZ (Western Renewable Energy Zones), RETI CPUC (Renewable Energy Transmission Initiative of the California Public Utilities Commission)

Unique Data ID	Environmental Category	Technology	Data Publisher Organization	Dataset Name	ORB 2015 (7)	BLM WSEP (60)	BLM WWW-MP (?)	BLM DRECP (8)	WECC (61)	WREZ (4)	RETI CPUC (3)
49	3	All	Montana Dept of Fish Wildlife and Parks: Crucial Areas Planning System (CAPS)	Bighorn Sheep & Mountain Goat Habitat	AV	NA	NA	NA	RC 3	NA	NA
103	3	All	BLM - WWWMP	Visual Resource Management lands level III	NA	NA	MLSC	NA	NA	NA	NA
104	3	All	Colorado Division of Wildlife	Species Activitiy Data: Severe Winter Range, Winter Concentration, Winter Range, Migration Patters, and Migration Corridor	NA	NA	NA	NA	RC 3	NA	NA
105	3	All	Montana Dept of Fish Wildlife and Parks: Crucial Areas Planning System (CAPS)	Big Game Winter Range Habitat	NA	NA	NA	NA	RC 3	NA	NA
111	3	All	Federal Highway Administration	America's Byways	NA	NA	NA	NA	RC 2	NA	NA
112	3	All	Caltrans	California Scenic Highways	NA	NA	NA	NA	RC 2	NA	NA
113	3	All	Idaho Department o Transportation	Scenic Byways of Idaho	NA	NA	NA	NA	RC 2	NA	NA
114	3	All	Colorado Department of Transportation	Colorado Scenic and Historic Byways	NA	NA	NA	NA	RC 2	NA	NA
115	3	All	Washington State Department of Transportation	Washington Scenic Highways	NA	NA	NA	NA	RC 2	NA	NA
118	3	All	Wyoming Game and Fish	Shapefile: WYPrairieDogComplexes_WGFDWAFWA.	NA	NA	NA	NA	NA	NA	NA
121	3	Wind	Wyoming Natural Heritage Program	WYGrasslandBirds	NA	NA	NA	NA	NA	NA	NA
123	3	All	WDFW	Deer Areas (Polygons)	NA	NA	NA	NA	NA	NA	NA
124	3	All	WDFW	Elk Areas (Polygons)	NA	NA	NA	NA	NA	NA	NA
125	3	All	U.S. Fish and Wildlife Service	USFWS Upland Species Recovery Units	AV	NA	NA	NA	NA	NA	NA
126	3	All	California Department of Conservation	Williamson act –Farmland Mapping and Monitoring Program (FMMP) in CA	Cat3	NA	NA	NA	EX	EX	EX
127	3	All	The Nature Conservancy	Mojave Desert Ecoregional Assessment	Cat3	NA	NA	NA	NA	NA	NA
129	3	All	Herpetological Conservation and Biology	Mojave Desert Tortoise Linkages	Cat3	NA	MLSC	NA	NA	NA	NA
133	3	All	BLM - WWWMP	Desert Tortoise Connectivity	NA	NA	MLSC	NA	NA	NA	NA
136	3	All	TNC	High integrity grasslands	NA	NA	NA	NA	NA	NA	NA
137	3	Wind	Playa Lakes Joint Venture	Playa clusters	NA	NA	NA	NA	NA	NA	NA
138	3	Wind	Colorado Parks and Wildlife	Greater prairie-chicken optimal habitat	NA	NA	NA	NA	NA	NA	NA
139	3	All	Colorado Natural Heritage Program	Potential Conservation Areas	NA	NA	NA	NA	NA	NA	NA
140	3	Wind	Colorado Parks and Wildlife	Columbian sharptail grouse production areas and winter range	NA	NA	NA	NA	NA	NA	NA
141	3	Wind	Colorado Parks and Wildlife	Plains sharptail grouse concentration areas, winter concentration areas, migratino corridors, severe winter range	NA	NA	NA	NA	NA	NA	NA
142	3	All	Colorado Parks and Wildlife	Pronghorn	NA	NA	NA	NA	NA	NA	NA
143	3	Wind	Colorado Parks and Wildlife	Least tern production areas and foraging areas	NA	NA	NA	NA	NA	NA	NA
144	3	Wind	Colorado Parks and Wildlife	Piipng plover production areas and foraging areas	NA	NA	NA	NA	NA	NA	NA
145	3	Wind	Colorado Parks and Wildlife	CPW Nest area and potential nesting area	NA	NA	NA	NA	NA	NA	NA
146	3	Wind	Wyoming Natural Heritage Program	Tree roosting bats (Silver-haired bat, Hoary, Eastern Red)	NA	NA	NA	NA	NA	NA	NA
148	3	All	New Mexico Department of Game and Fish	Big Game Priority Habitat	NA	NA	NA	NA	RC 3	NA	NA
149	3	All	Oregon Department of Fish and Wildlife	Elk and Deer Winter Range	NA	NA	NA	NA	RC 3	NA	NA
150	3	All	New Mexico Department of Transportation	New Mexico State and National Scenic Byways	NA	NA	NA	NA	RC 2	NA	NA
151	3	All	Oregon Department of Transportation	Oregon Scenic Byways	NA	NA	NA	NA	RC 2	NA	NA
152	3	All	Wyoming Department of Transportation	Wyoming Scenic Highways and Byways	NA	NA	NA	NA	RC 2	NA	NA
155	3	All	USFWS	Columbian white-tailed deer	NA	NA	NA	NA	NA	NA	NA
156	3	All	BLM	BLM Nominated ACECs	NA	NA	NA	NA	NA	NA	NA
157	3	All	TNC	TNC Nominated ACECs. Areas with high conservation value as determined through TNC ecoregional analysis (if/when they become ACEC they would move up to Cat 2).	NA	NA	NA	NA	NA	NA	NA
159	3	All	TNC OR	The Nature Conservancy Portfolio Areas	Cat3	NA	NA	NA	NA	NA	NA
160	3	All	ODFW	Oregon Conservation Strategy	NA	NA	NA	NA	NA	NA	NA
161	3	All	TNC	TNC Nevada priority landscapes layer	NA	NA	NA	NA	NA	NA	NA
162	3	All	NDOW	Critical habitat rank 1 or 2	NA	NA	NA	NA	NA	NA	NA
164	3	All	Arizona Department of Roads	Arizona Scenic Roads	NA	NA	NA	NA	RC 2	NA	NA

Table S19. Datasets for Environmental Exclusion Category 3. Definitions: Exclude development (EX), Avoid development (AV), WECC Environmental Risk Class (RC 1, 2, 3, 4), BLM High Level Siting Considerations (HLSC), BLM Moderate Level Siting Considerations (MLSC), Information not available (NA). Study abbreviation and names: ORB 2015 (Optimal Renewable Energy Build-out), BLM WSEP (BLM Western Solar Energy Program), BLM WWW-MP (BLM West-wide Wind Mapping Program), WECC (Western Electricity Coordinating council Environmental Data Viewer), WREZ (Western Renewable Energy Zones), RETI CPUC (Renewable Energy Transmission Initiative of the California Public Utilities Commission)

Unique Data ID	Environmental Category	Technology	Data Publisher Organization	Dataset Name	ORB 2015 (7)	BLM WSEP (60)	BLM WWW-MP (?)	BLM DRECP (8)	WECC (61)	WREZ (4)	RETI CPUC (3)
170	3	All	CEC and USGS, Las Vegas Field Station	Mohave Ground Squirrel (candidate species) Maxent site suitability model at 0.438 cutoff	Cat4	NA	NA	NA	NA	NA	NA
187	3	All	TNC	The Nature Conservancy Portfolio Areas	NA	NA	NA	NA	NA	NA	NA
241	3	All	Colorado natural Heritage Program	Potential Conservation Areas (CO)	NA	NA	NA	NA	NA	NA	NA
259	3	All	BLM - WWWMP	Sage Grouse General Habitat Management Area, High Level Siting Requirements	NA	NA	HLSC	NA	NA	NA	NA
260	3	All	BLM - WWWMP	Sage Grouse General Habitat Management Area, Moderate Level Siting Requirements	NA	NA	MLSC	NA	NA	NA	NA
261	3	All	BLM - WWWMP	Sagebrush Focal Area	NA	NA	EX	NA	NA	NA	NA
267	3	All	NRCS	Westwide Prime farmland classification	NA	NA	NA	NA	NA	NA	EX
268	3	All	TNC	Priority Conservation Areas	NA	NA	NA	NA	NA	NA	NA
269	3	All	NatureServe	Mojave Desert Tortoise Species Distribution Model - Threshold	NA	NA	NA	NA	NA	NA	NA
300	3	All	Bureau of Land Management	Desert Tortoise (gopherus agassizii suitable habitat in Arizona)	NA	NA	NA	NA	NA	NA	NA
303	3	All	The Nature Conservancy	GAP_StsPNW_V2_20200326	NA	NA	NA	NA	NA	NA	NA
304	3	All	NDOW	Occupied_x_Distribution	NA	NA	NA	NA	NA	NA	NA
305	3	All	NDOW	Movement Corridors	NA	NA	NA	NA	NA	NA	NA
308	3	All	USFWS	Grassland Birds Predicted Core Areas	NA	NA	NA	NA	NA	NA	NA
309	3	All	TNC MT	The Nature Conservancy Portfolio Areas	NA	NA	NA	NA	NA	NA	NA
312	3	All	Idaho Department of Fish and Game	Idaho Crucial Habitat	NA	NA	NA	NA	NA	NA	NA
313	3	All	New Mexico Department of Fish and Game and Natural Heritage	New Mexico Crucial Habitat	NA	NA	NA	NA	NA	NA	NA
315	3	All	The Nature Conservancy	Resilient Connected Network	NA	NA	NA	NA	NA	NA	NA
157a	3	All	The Nature Conservancy NV	Areas of Critical Environmental Concern	NA	NA	NA	NA	NA	NA	NA
157b	3	All	TNC OR	The Nature Conservancy Portfolio Areas	NA	NA	NA	NA	NA	NA	NA
173	3	All	AGFD	AZ multi-species corridors	NA	NA	NA	NA	NA	NA	NA

Table S20. Offshore Wind Datasets for Environmental Exclusion Categories.

Unique Data ID	Environmental Category	Technology	Data Publisher Organization	Dataset Name
1	1	Offshore	Protected Seas	Marine Managed Areas (Most Restrictive)
2	1	Offshore	NOAA	Marine Protected Areas (No Take)
3	1	Offshore	Oregon Coastal Management Program Department of Land Conservation and Development	Oregon Territorial Sea Plan Part Five, 2019 (Renewable Energy Exclusion Area)
4	2	Offshore	Protected Seas	Marine Managed Areas (Heavily Restrictive and Moderately Restrictive)
5	2	Offshore	NOAA	Marine Protected Areas (No Access)
6	2	Offshore	Oregon Coastal Management Program Department of Land Conservation and Development	Oregon Territorial Sea Plan Part Five, 2019 (Resources and Uses Management Area)
7	2	Offshore	NOAA	Habitat Areas of Particular Concern within Essential Fish Habitat
8	3	Offshore	Washington State Ocean Caucus	Ecologically Important Areas
9	3	Offshore	The Nature Conservancy	Marine Portfolio Areas California
10	3	Offshore	The Nature Conservancy	Marine Portfolio Areas Pacific Northwest
11	3	Offshore	Oregon Coastal Management Program Department of Land Conservation and Development	Oregon Territorial Sea Plan Part Five, 2019 (Resources and Uses Conservation Area)
12	3	Offshore	The Nature Conservancy	Oregon Rocky Substrate
13	3	Offshore	Oregon Department of Fish and Wildlife Marine Resources Program	Oregon Nearshore Ecological Data Atlas
14	3	Offshore	The Nature Conservancy	California Bathymetry Complexity

3. Additional results

A. Energy modeling results. In most net-zero economy-wide cases, electricity is needed not only to electrify many current fuel uses, but also to meet energy conversion loads that don't currently exist, including electrolysis, dual-fuel boilers, and direct air capture (DAC). Compared to Electricity Only cases, these new uses and loads resulted in a further 25-85% increase in electricity demand (Fig. S15A). Hydrogen from electrolysis was used directly in industry and heavy transportation, and also in synthesizing hydrocarbon fuels for applications such as jet fuel and chemical feedstocks. The amount of hydrogen needed for fuel synthesis depends on the extent of electrification and the availability of biomass or fossil fuels with emissions offsets.

In all net-zero cases, even Renewables Only, thermal generating capacity was needed (primarily gas combined cycle without carbon capture), along with energy storage and flexible loads, to balance electricity supply and demand in real time (Fig. S15C). Carbon constraints for thermal power plants were met by either burning carbon-neutral fuels (in the Renewables Only case), or by burning fossil natural gas and offsetting it with negative emissions.

During the 2020s, solar and wind build rates across all scenarios needed only modest increases relative to 2020. However, in 2030-2050, in all cases except Limited Wind and Solar, the rate of new generating capacity construction reached 3-9 times the maximum historic rate of 7 GW/year, with the lowest rates in the High Electrification case and the highest in the Renewables Only case (Fig. S16, Table S20). When the growth rate was constrained to the historic rate in the Limited Wind and Solar case, a large amount of nuclear and natural gas with CCS capacity was required to meet the net zero goal (Fig. S16). Put differently, avoiding a large build-out of nuclear and gas with CCS in order to meet climate goals requires an average build rate for wind and solar that is a minimum of 2-3 times the historic rate on average over the next three decades.

The rate of growth in biofuel production is also the greatest in the 2040s (Fig. S16, Fig. S18), when the growth in transportation electrification and clean fuels demand also reach their peak.

During the 2020s, modeled renewable build rates across all scenarios needed only modest increases relative to 2020. However, over the subsequent 20 years, the build rate requirements dramatically increased. Between 2030 and 2050, the High Electrification SL3 scenario had an average requirement of 17 GW (4 GW of wind, 13 GW of solar) per year, more than double the historical maximum rate (Fig. S16). These build rates increased further in the Slow Electrification and Renewables Only cases, reaching a maximum of 54 GW/year from 2046-2050 for solar in the Renewables Only scenario. The Limited Wind and Solar case, by contrast, had wind and solar build rates capped at the maximum historical level, resulting in the need for 4 GW of new clean firm resources annually from 2030 to 2050. The specific clean firm resources selected by the model changed over time from gas with CCS to geothermal to nuclear as a matter of economics, resulting in total installed capacities of 18.4 GW, 17.8 GW, and 50.7 GW respectively in 2050 (Table S20). For nuclear, existing capacity in the West is 5.2 GW, or 10% of the total capacity needed in this scenario by 2050. The model results show that if wind and solar build rates do not reach the required level, and if clean firm resources also cannot be built at the required rate, then an economy-wide net-zero target cannot be achieved by 2050 using the technologies commercially available today.

B. Sensitivity results. The In-State, Limited Biomass, and Limited Wind and Solar cases had the smallest new area requirements among net-zero cases, ranging from 55,000 to 78,000 km^2 (Fig. S13). The In-State case assumed restrictions on electricity transmission across regions. This resulted in using higher-cost solar located within southwestern states rather than importing lower-cost wind from states like Wyoming or New Mexico, raising costs (Fig. 5 in main text) but reducing the land needed for transmission and onshore wind. The Limited Biomass assumptions avoided the land needed for purpose-grown biomass but increased the land needed for wind and solar capacity for synthetic fuel production to compensate; the net effect was a reduction in land use (Figs. S13, S20). The Limited Wind and Solar case had lower capacity of both wind and solar, significantly reducing land area requirements; additional capacity needs were met by nuclear and natural gas with CCS that require less land area.

Table S21. Summary of energy scenario results for 2050 for all decarbonization scenarios

Indicator	Units	Reference		High Electrification			Limited Biomass	Limited Wind and Solar	In-state	Slow Electrification			Renewables Only		
		2020	2050	SL1	SL2	SL3	SL3	SL3	SL3	SL1	SL2	SL3	SL1	SL2	SL3
**Emissions**															
Gross Energy CO2	Mt-CO2	807	851	61	59	64	92	156	68	95	175	162	0	0	0
Uncombusted & bunkered CO2	Mt-CO2	-54	-55	-46	-45	-45	-45	-45	-45	-46	-45	-45	-45	-45	-45
Geologically Sequestered CO2	Mt-CO2	0	0	-60	-59	-64	-92	-156	-68	-94	-175	-162	0	0	0
Land Sink CO2	Mt-CO2	-88	-71	-104	-104	-104	-104	-104	-104	-104	-104	-104	-104	-104	-104
Non-energy & Non-CO2	Mt-CO2e	27	34	199	199	199	199	199	199	199	199	199	199	199	199
Net Emissions	Mt-CO2e	692	759	50	50	50	50	50	50	50	50	50	50	50	50
**Electricity (total generation and total capacity)**															
Total generation	TWh	712	978	1922	1959	1862	1998	1619	1858	1967	2100	2005	2504	2751	2763
End-use load (w losses)	TWh	712	944	1529	1532	1532	1533	1514	1520	1283	1287	1286	1531	1531	1536
Fuel conversion load	TWh	0	34	393	427	330	465	105	338	684	813	719	973	1220	1227
Renewable curtailment	%	0.7%	3.4%	2.6%	2.6%	2.5%	2.5%	0.4%	2.5%	2.4%	2.3%	2.1%	2.3%	2.2%	1.9%
Wind	GW	27.2	50.1	195.9	192	144.5	154.6	95.8	129.6	216.9	225.3	158.9	254.4	263.1	178.3
Offshore wind	GW	0	3.7	15.3	15	16	18.2	27.3	19.9	7.4	20.6	23.9	16.9	19	27.2
Onshore wind	GW	27.2	46.4	180.6	177	128.5	136.4	68.5	109.7	209.5	204.7	135	237.5	244.1	151.1
Solar	GW	39.4	136.2	331.8	373	413.9	454.7	198.1	440	308.5	370.7	454.5	489.5	587.6	744.2
Rooftop solar	GW	14	34.5	34.5	57.5	80.5	80.5	89.7	80.5	34.5	57.5	80.5	34.5	57.5	80.5
Urban infill solar	GW	0.3	31.5	31.5	52.5	73.5	73.5	73.5	92.3	31.5	52.5	73.5	31.5	52.5	100.3
Large-scale solar	GW	25.1	70.2	265.8	263	259.9	300.7	34.9	267.2	242.5	260.7	300.5	423.5	477.6	563.4
Storage	GW	5.4	11.6	47.4	57.9	80.3	89.8	23.4	95.2	36.3	50.9	80.6	52.6	64.2	101.3
Hydro	GW	50	47.8	48.7	49.2	49.8	49.8	50	49.8	49.2	49.9	49.9	49.1	49.2	49.9
Biomass	GW	1.7	0.4	2.7	1.5	1.9	1.6	2.1	2.6	4	1.9	2.3	1.1	0.5	0.6
Geothermal	GW	2.4	5.7	14.9	15	17.5	17.4	17.8	17.5	14.9	15	15	14.9	14.9	16.2
Nuclear	GW	7.5	5.3	5.2	5.3	5.3	5.3	50.7	5.3	5.3	5.3	5.3	0	0	0
Coal	GW	24.3	10	0	0.1	0.1	0.1	0.1	0.1	0.1	0.1	0.1	0	0	0
Gas	GW	79.5	67.2	81.8	81.3	80.8	78.6	73.8	82.8	54	50.3	51.2	75.2	74.5	75
Gas Capacity Factor	%	33%	38%	4%	3%	4%	2%	2%	4%	2%	2%	2%	2%	1%	1%
Gas w/ CCS	GW	0	0.2	0.2	1.4	2.9	2.6	18.4	5	0.2	0.2	0.3	0.1	0.1	0.1
**Transmission Capacity**															
Gas w/ CCS	GW	24.3	10	0	0.1	0.1	0.1	0.1	0.1	0.1	0.1	0.1	0	0	0
Total Transmission capacity	GW	67	70	104	105	98	98	79	76	103	103	93	114	113	103
New Transmission Capacity	GW		0	34	34	27	27	7	7	32	31	22	44	43	32
New Renewables Spurline Capacity	GW		89	424	422	353	403	70	346	434	443	402	648	708	687
New HV Transmission Miles	Miles		0	6,632	5,737	6,259	6,259	2,341	0	6,325	5,737	5,849	8,421	7,458	6,713
Reconductored or Co-located Tx Miles	Miles		920	7,949	7,570	6,730	5,960	2,286	3,736	10,745	10,178	7,176	10,004	10,027	7,549
New Renewables Spurline Miles	Miles		25,233	119,819	146,729	133,108	143,997	45,260	108,366	126,559	169,331	140,836	156,247	232,745	176,354
**Biomass**															
Consumption (direct + embodied)	EJ	0.76	1.37	3.2	3.04	3.33	2.03	2.99	3.34	5.08	2.55	3.21	2.17	1.26	1.24
Production	EJ	0.58	0.66	0.89	0.68	0.69	0.66	0.7	0.69	1	0.7	0.7	0.96	0.69	0.69
**Carbon Capture Utilization and Storage**															
E&I CO2 captured	Mt	0	3	77	76	73	108	156	77	137	213	194	68	88	88
E&I CO2 utilized	Mt	0	3	17	17	9	16	0	9	43	38	32	68	88	88
E&I CO2 sequestered	Mt	0	0	60	59	64	92	156	68	94	175	162	0	0	0
DAC Capacity	Mt/year	0	0	7	19	9	54	32	10	73	178	155	17	51	53
**Net Energy Supply Cost (Costs Shown Relative to "High Electrification SL1")**															
2050 Levelized Cost	$B	NA	NA	0	4.7	7.8	10.2	25.3	9.7	38.8	44.7	49.2	10.0	16.6	23.4
NPV Cost (2020-2050)	$B	NA	NA	0	45	74	86	154	85	505	586	620	64	111	154

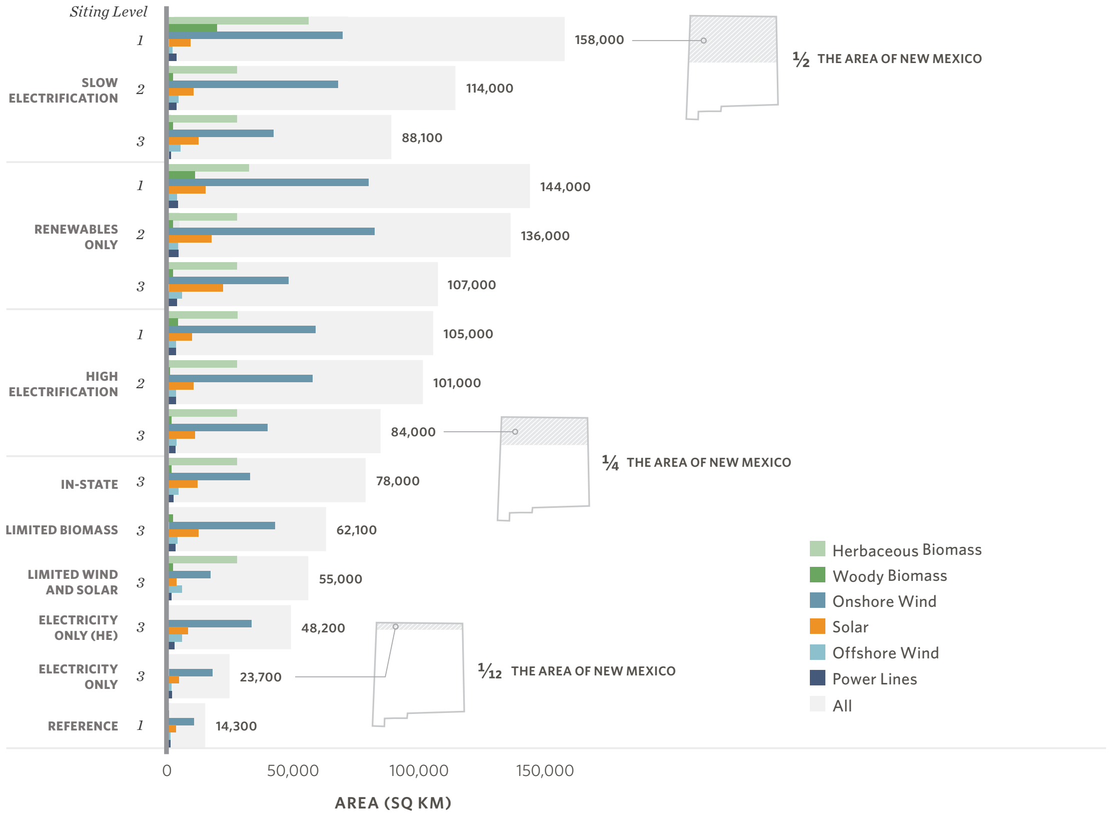

Fig. S13. Land and ocean area for renewable resource and additional biomass resources required in 2050 for all scenarios. This figure is the same as Figure 1 in the main paper, except that it includes the sensitivity energy cases, In-State, Limited Biomass, the Limited Wind and Solar. See Figure 1 for the complete caption.

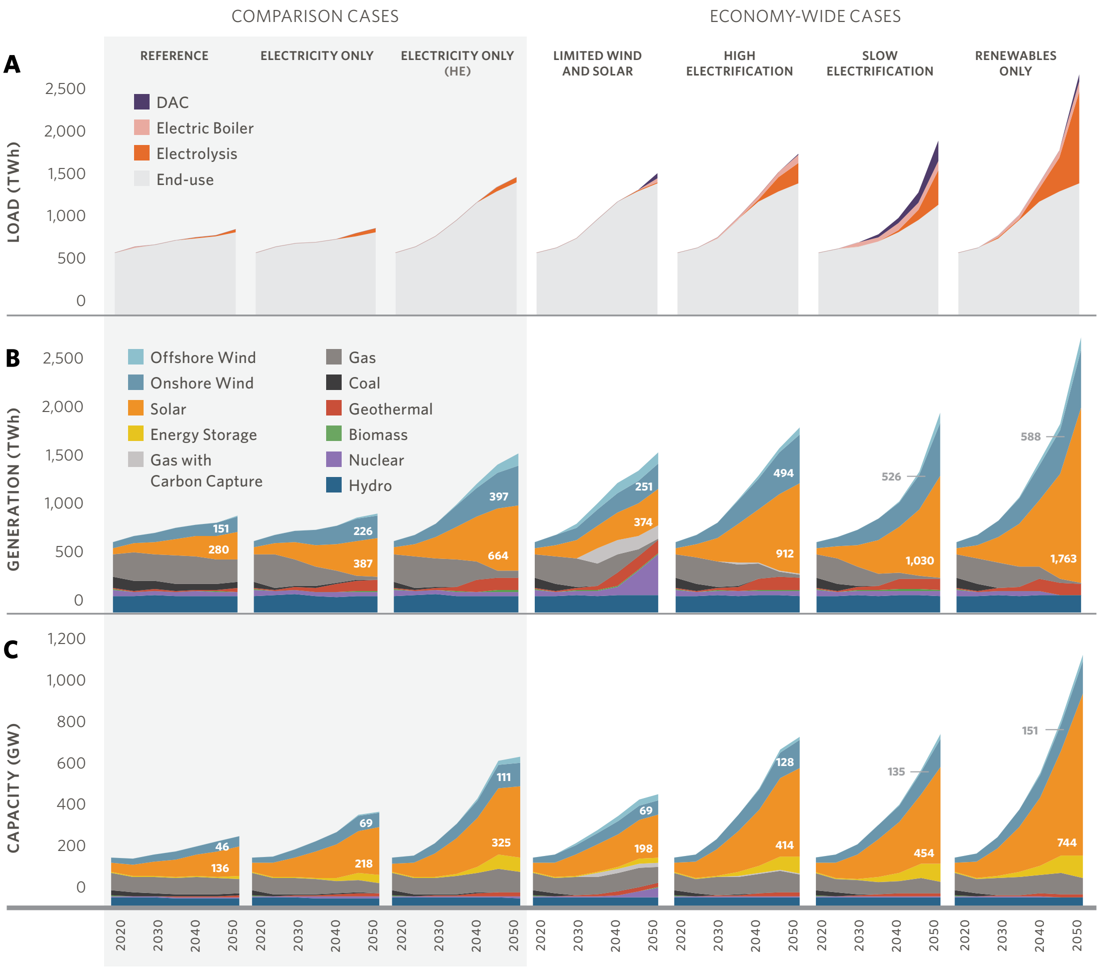

Fig. S14. Electricity demand (Load) (A), generation (B), and installed capacity (C) across the western U.S. for scenarios that achieve different decarbonization targets (reference, electricity only, and economy-wide net zero cases) or with different decarbonization pathways (High Electrification, Slow Electrification, Renewables Only). This figure is the same as Figure 2 in the main paper, except that it includes the sensitivity energy cases, In-State, Limited Biomass, the Limited Wind and Solar. See Figure 2 for the complete caption.

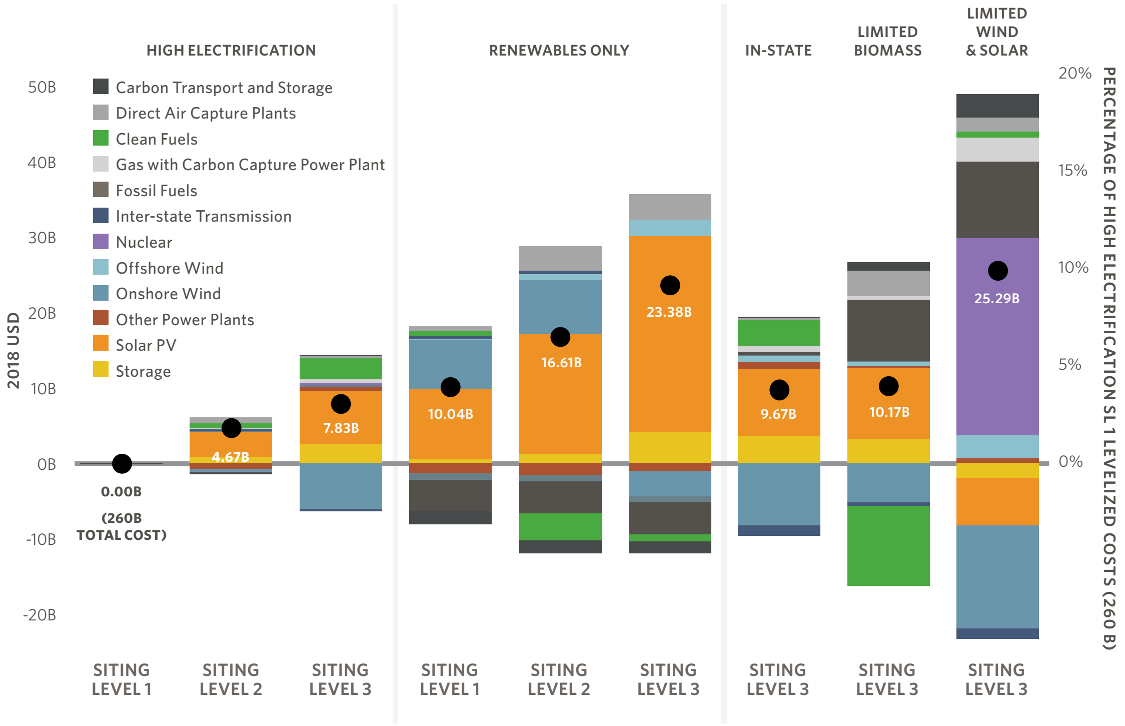

Fig. S15. Net annual levelized supply-side cost in 2050 for scenarios are shown relative to the High Electrification Siting Level 1 scenario. Bars above the x-axis represent and increase in costs while bars below represent a decrease in relative cost. This figure is the same as Figure 4 in the main paper, except that it includes the sensitivity energy cases, In-State, Limited Biomass, the Limited Wind and Solar. See Figure 4 for the complete caption.

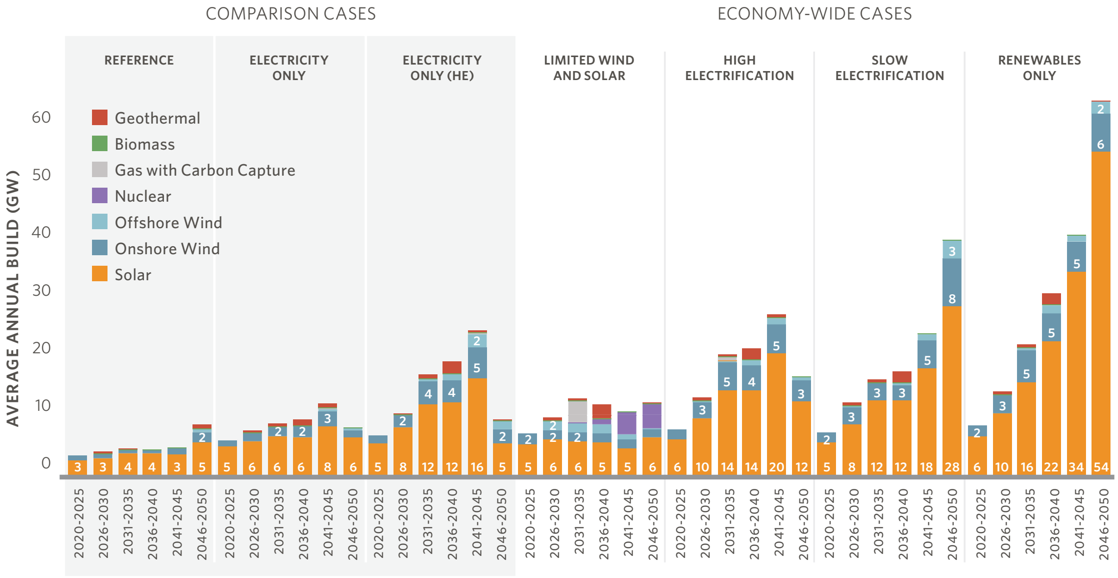

Fig. S16. Average annual generation capacity build rates by 5-year period for the reference case contrasted against different decarbonization cases using Siting Level 3 (SL3) protections

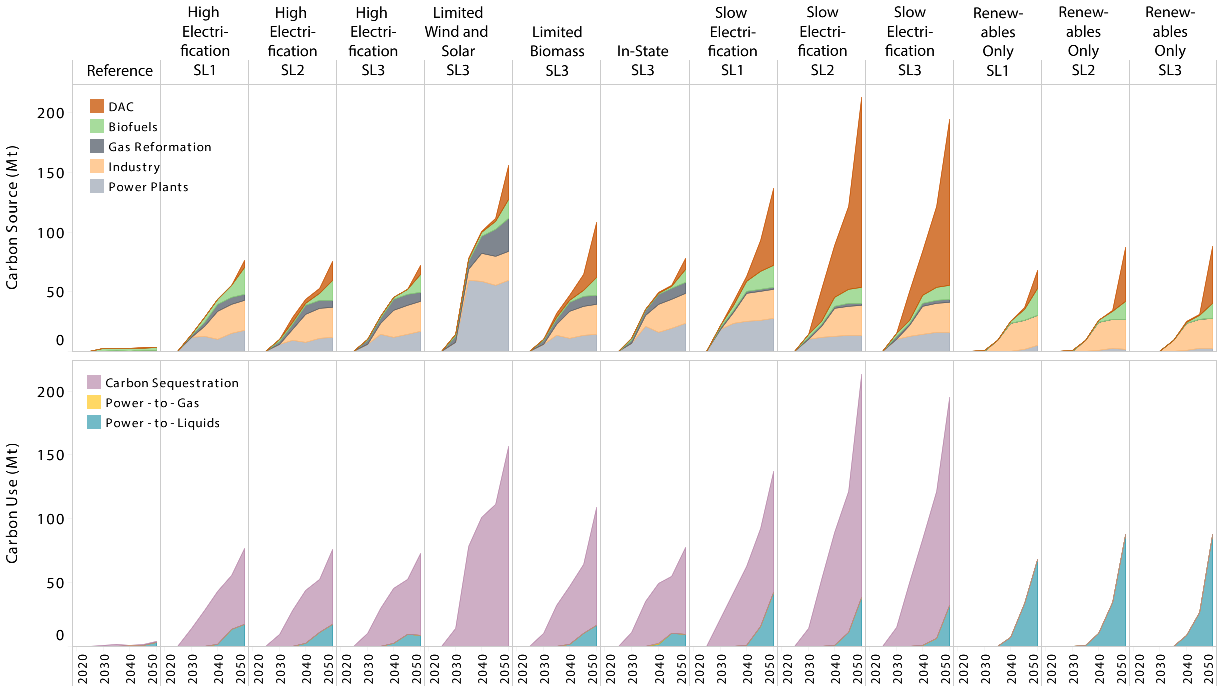

Fig. S17. Carbon emissions, storage, and use

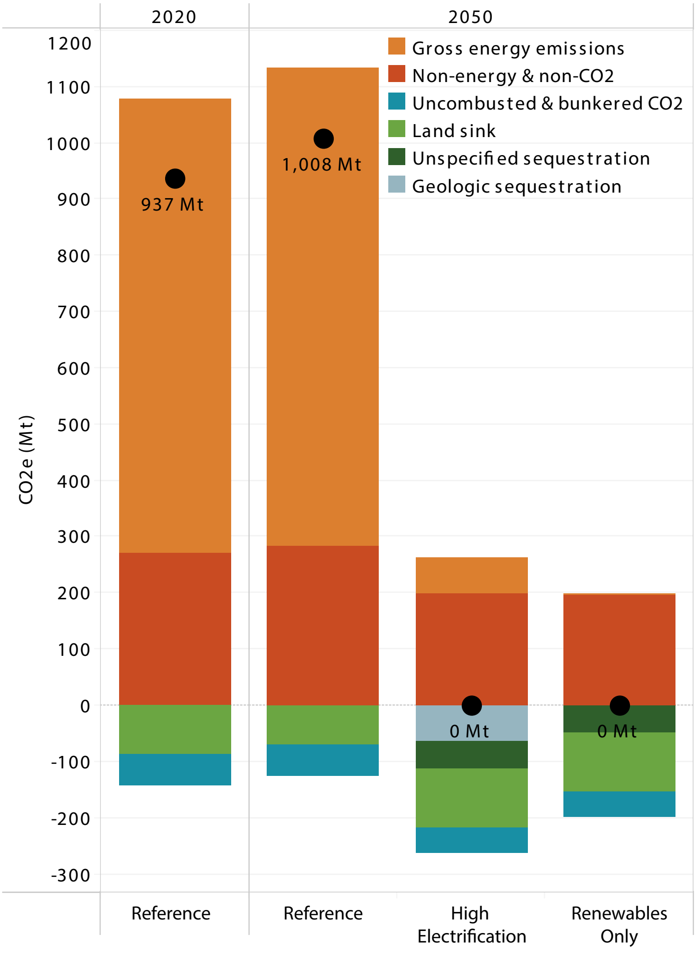

Fig. S18. Carbon emissions accounting

Grace C. Wu*, Ryan A. Jones, Emily Leslie, James H. Williams, Andrew Pascale, Erica Brand, Sophie S. Parker, Brian S. Cohen, Joseph E. Fargione, Julia Prochnik, Maya Batres, Mary G. Gleason, Michael H. Schindel, Benjamin M. Sleeter, Charlotte K. Stanley

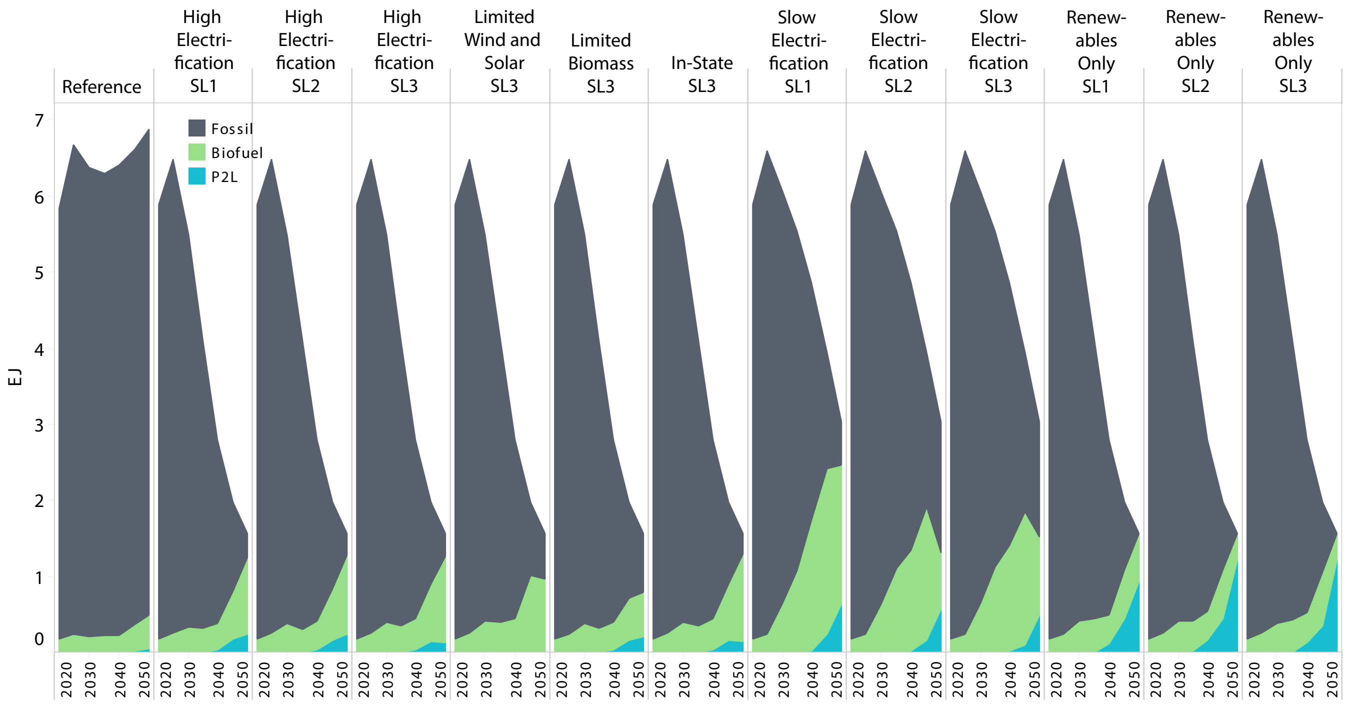

Fig. S19. Fuel sources

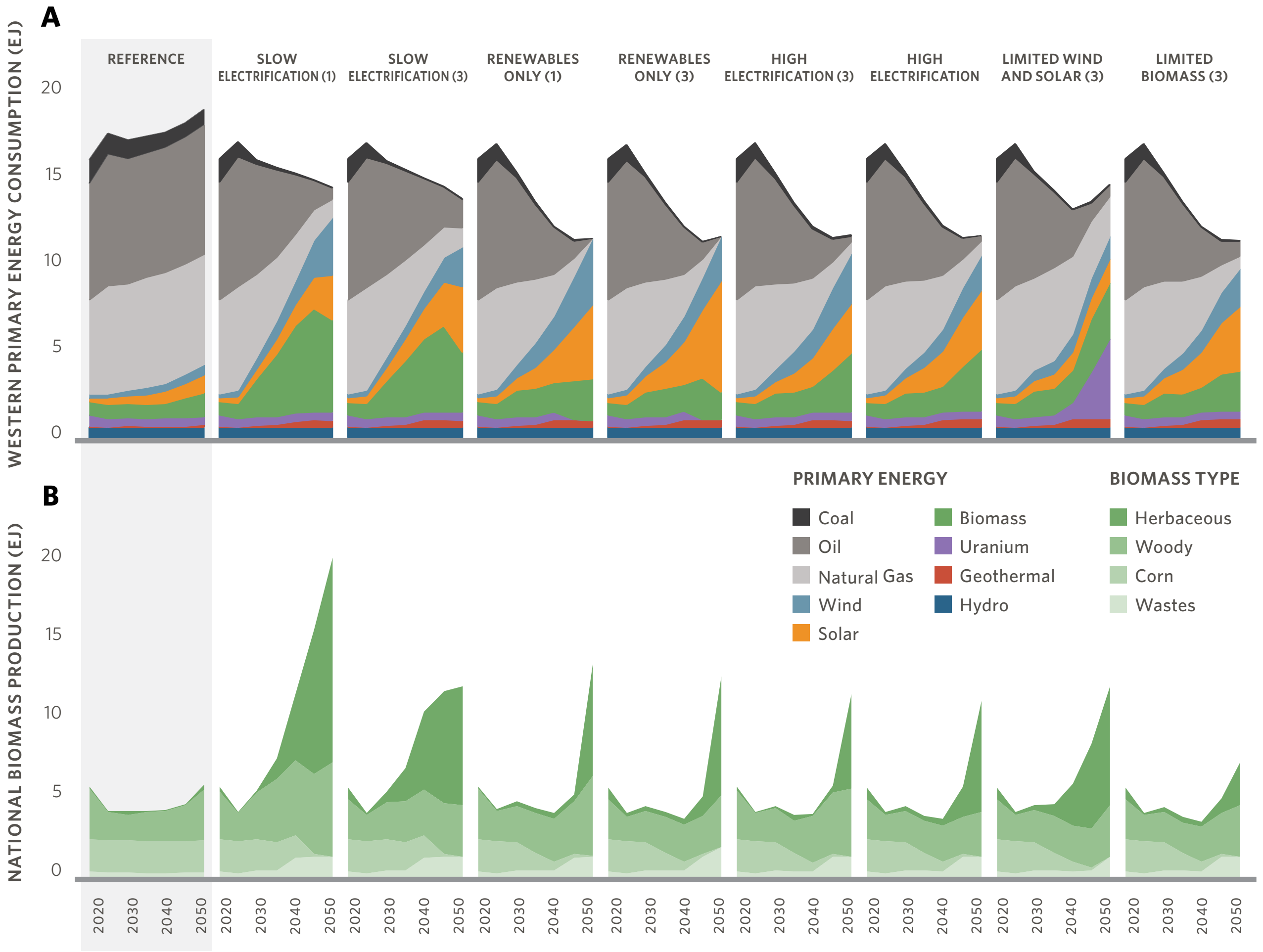

Fig. S20. Primary energy and biomass use

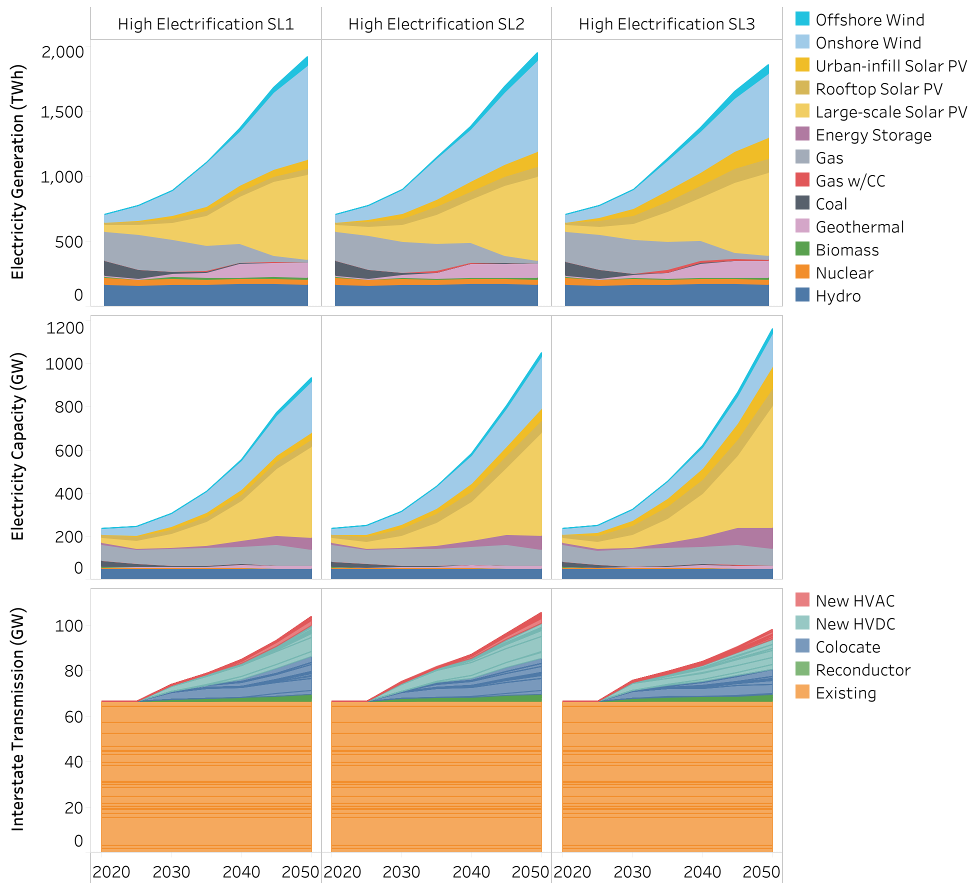

Fig. S21. Generation, capacity, and transmission for High Electrification Siting Levels 1-3.

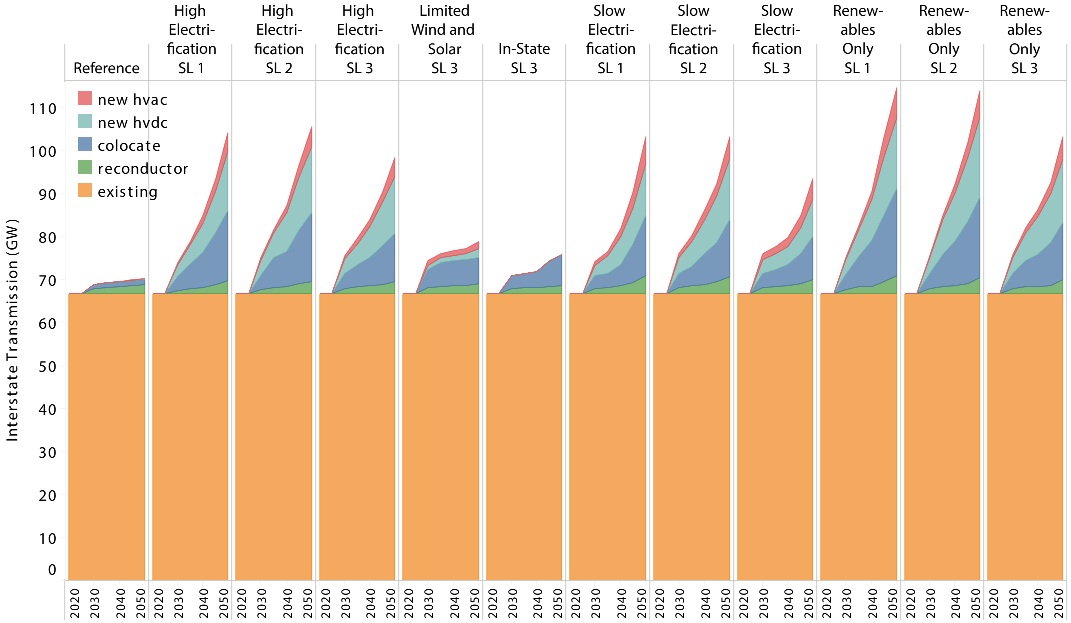

Fig. S22. Existing and new transmission capacity for the reference scenario and all decarbonization scenarios.

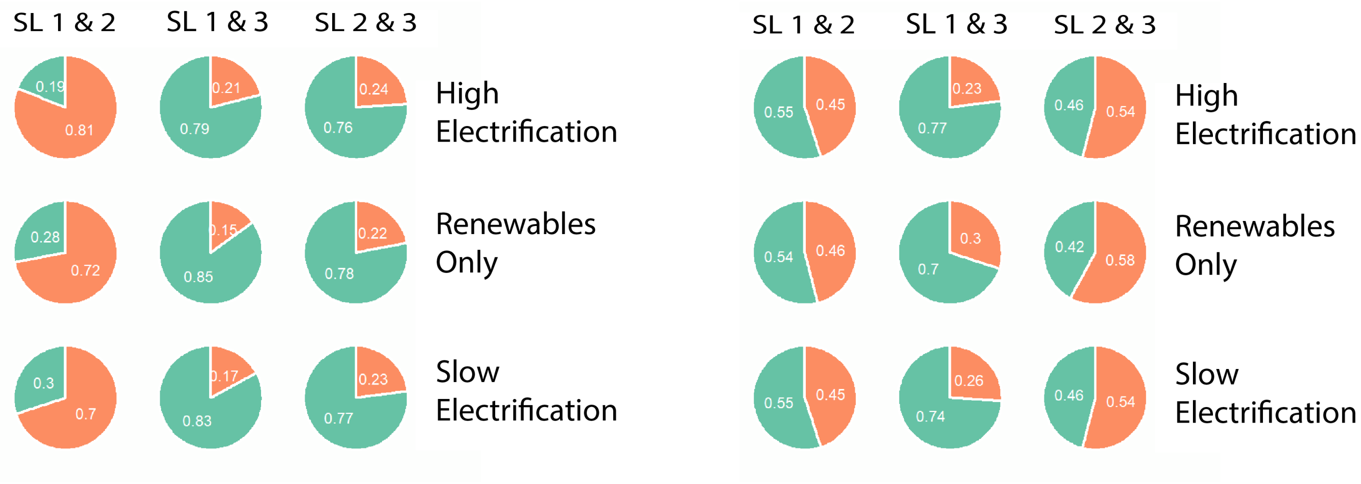

(a) Solar **(b)** Wind

Fig. S23. Percentage overlap between Siting Levels for solar (a) and wind (b) selected project areas. “1 2” indicates overlap between Siting Levels 1 and 2 and “2 3” indicates overlap between Siting Levels 2 and 3.

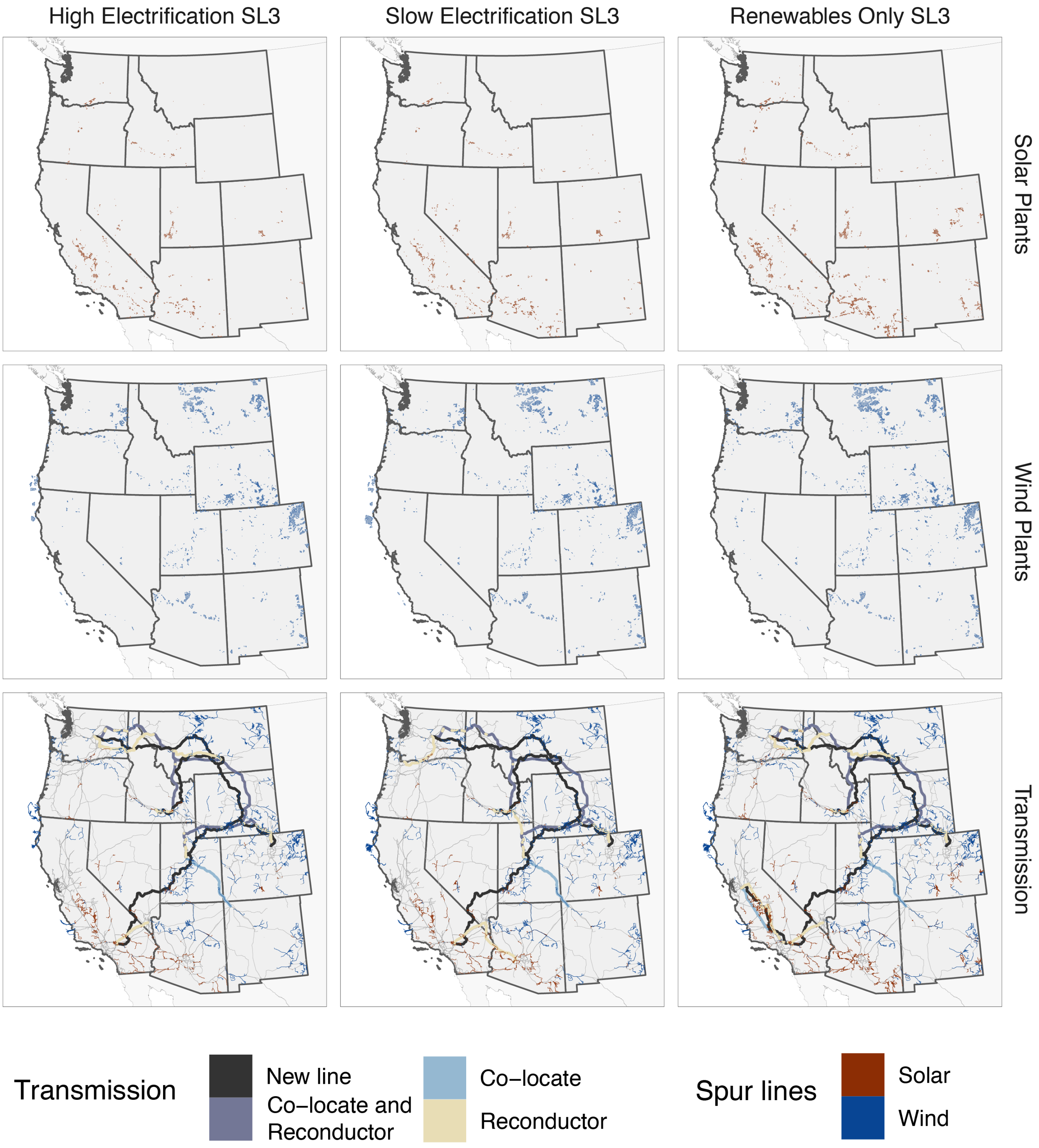

Fig. S24. Wind, solar, and power line build-out maps for Siting Level 3 of three main decarbonization cases (High Electrification, Slow Electrification, Renewables Only)

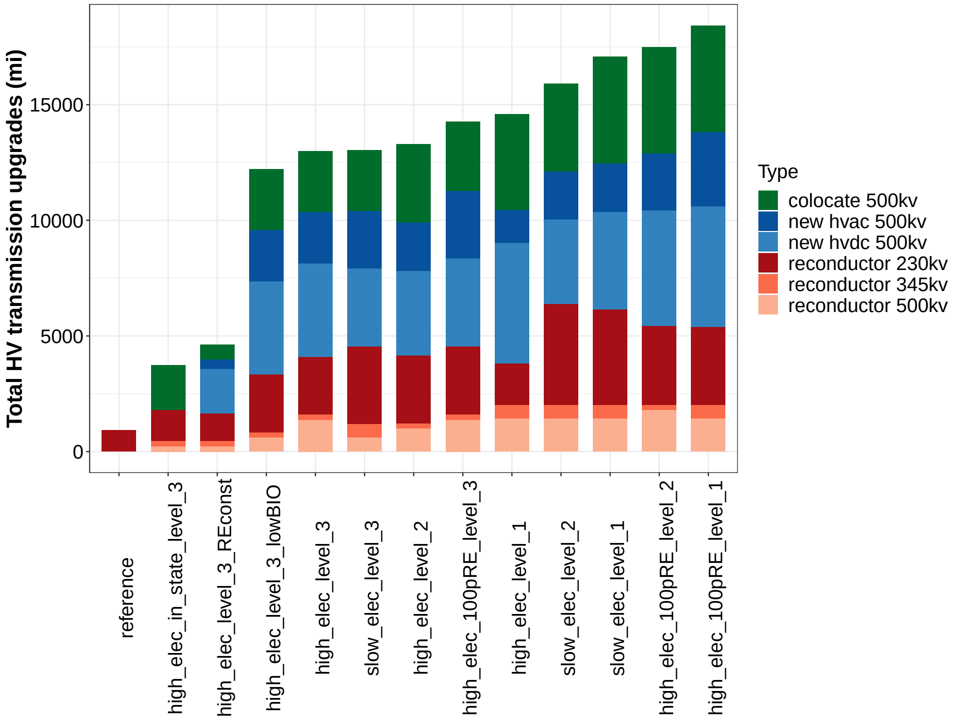

Fig. S25. Spatial downscaling results showing total transmission upgrades in each scenario

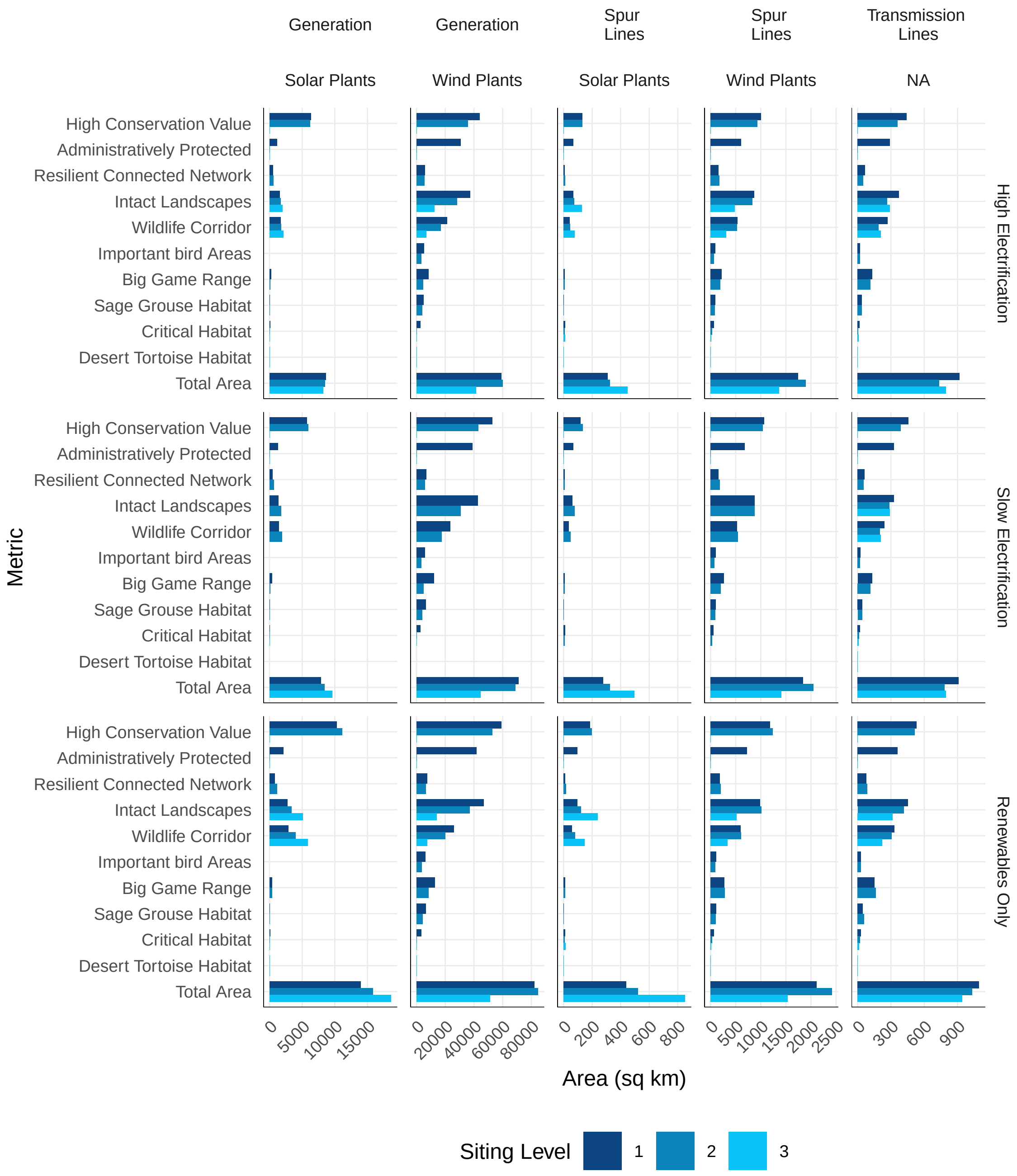

Fig. S26. Environmental metrics for generation, transmission, and spur lines for the three core economy-wide cases at all Siting Levels.

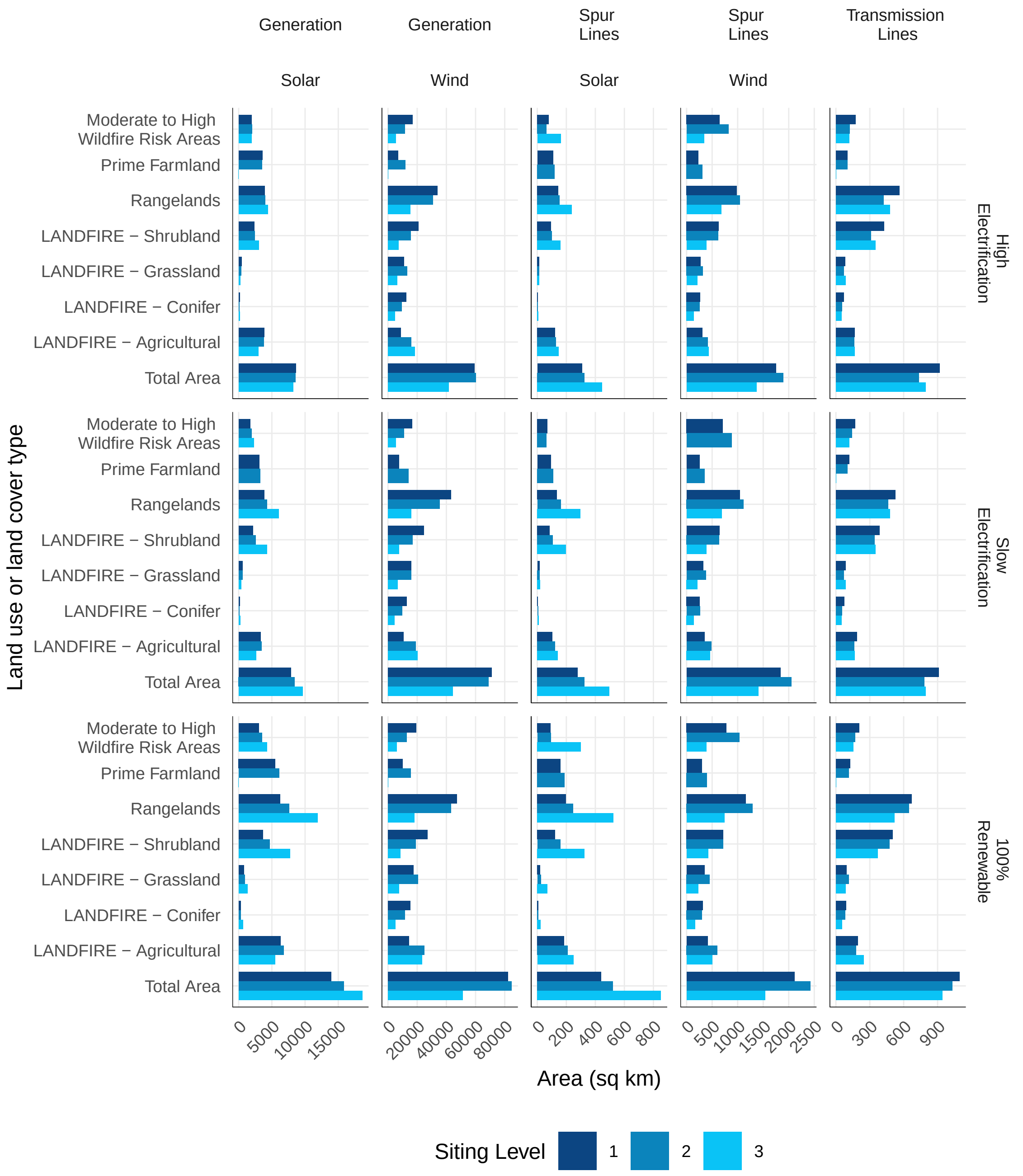

Fig. S27. Land use and land cover impacts for generation, transmission, and spur lines for the three core economy-wide cases at all Siting Levels.

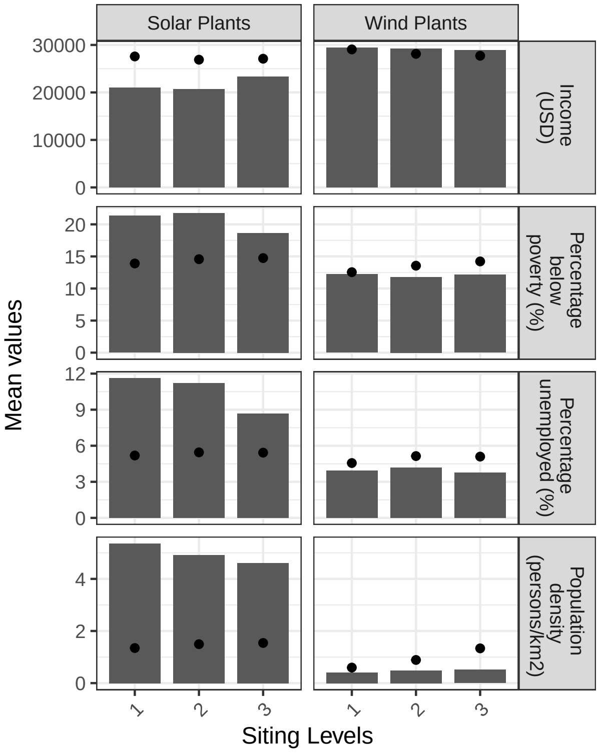

Fig. S28. Social vulnerability index indicators. Points represent the values for candidate project areas whereas the bars show the mean values for selected project areas.)

 Grace C. Wu*, Ryan A. Jones, Emily Leslie, James H. Williams, Andrew Pascale, Erica Brand, Sophie S. Parker, Brian S. Cohen, Joseph E. Fargione, Julia Prochnik, Maya Batres, Mary G. Gleason, Michael H. Schindel, Benjamin M. Sleeter, Charlotte K. Stanley

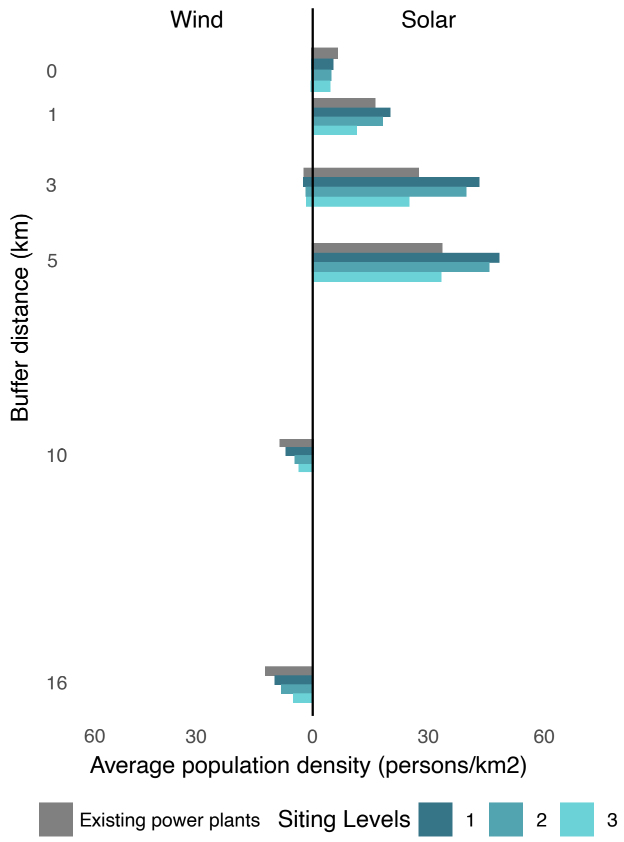

Fig. S29. Average population density of buffered areas around selected wind and solar project areas in the High Electrification scenarios.)

References

1. GC Wu, et al., Low-impact land use pathways to deep decarbonization of electricity. *Environ. Res. Lett.* **15**, 074044 (2020) 00001 Publisher: IOP Publishing.
2. GC Wu, et al., Land Conservation and Clean Energy Pathways for California, (The Nature Conservancy), Technical Report (2019).
3. California Public Utilities Commission [CPUC], Renewable Energy Transmission Initiative (RETI) Phase 1B, Technical report (2009).
4. R Pletka, J Finn, Western Renewable Energy Zones, Phase 1: QRA Identification Technical Report, (Black & Veatch and National Renewable Energy Laboratory), Technical Report NREL/SR-6A2-46877 (2009).
5. WECC EDTF, Environmental Recommendations for Transmission Planning, Technical report (2011).
6. A Lopez, B Roberts, D Heimiller, N Blair, G Porro, U.S. Renewable Energy Technical Potentials: A GIS-Based Analysis, (National Renewable Energy Laboratory, Golden, CO), Technical Report NREL/TP-6A20-51946 (2012).
7. GC Wu, et al., Integrating Land Conservation and Renewable Energy Goals in California: A Study of Costs and Impacts Using the Optimal Renewable Energy Build-Out (ORB) Model, (The Nature Conservancy), Technical Report (2015).
8. Renewable Energy Action Team, Desert Renewable Conservation Plan (2016).
9. The Nature Conservancy, Resilient Land Mapping Tool (2020) 00000.
10. , *System Advisor Model.* (National Renewable Energy Laboratory, Golden, CO), (year?).
11. J King, A Clifton, BM Hodge, Validation of Power Output for the WIND Toolkit, (National Renewable Energy Laboratory), Technical Report NREL/TP-5D00-61714 (2014).
12. GC Wu, et al., Strategic siting and regional grid interconnections key to low-carbon futures in African countries. *Proc. Natl. Acad. Sci.* p. 201611845 (2017) 00017.
13. GC Wu, MS Torn, JH Williams, Incorporating Land-Use Requirements and Environmental Constraints in Low-Carbon Electricity Planning for California. *Environ. Sci. & Technology* **49**, 2013–2021 (2015) 00012.
14. LM Miller, DW Keith, Climatic Impacts of Wind Power. *Joule* (2018) 00000.
15. LM Miller, DW Keith, Corrigendum: Observation-based solar and wind power capacity factors and power densities (2018 Environ. Res. Lett. 13 104008). *Environ. Res. Lett.* **14**, 079501 (2019) 00001.
16. R Borrmann, K Rehfeldt, AK Wallasch, S Luers, Capacity densities of European offshore wind farms, (European Union), Technical report (2018).
17. C Garcia, C Kavalec, California Energy Demand Updated Forecast, 2017 – 2027, (California Energy Commission), Technical Report CEC-200-2016-016-CMF (2017).
18. P Gagnon, R Margolis, J Melius, C Phillips, R Elmore, Estimating rooftop solar technical potential across the US using a combination of GIS-based methods, lidar data, and statistical modeling. *Environ. Res. Lett.* **13**, 024027 (2018) 00004.
19. MH Langholtz, BJ Stokes, LM Eaton, Billion-Ton Report: Advancing Domestic Resources for a Thriving Bioeconomy. *United States* (2016) 00095.
20. E Larson, et al., Net-Zero America: Potential Pathways, Infrastructure, and Impacts, final report summary. *Princet. Univ.* (2021) 00048.
21. N Carr, T Fancher, A Freeman, H Battles Manley, Surface area of solar arrays in the conterminous United States: U.S. Geological Survey data release (2016) 00000.
22. HIFLD, Electric Substations (2020).
23. T Mason, T Curry, D Wilson, Capital costs for transmission and substations, Recommendations for WECC Transmission Expansion Planning, (Western Electricity Coordinating Council), Technical report (2012).
24. U DOE, National Electric Transmission Congestion Study, (U.S. Department of Energy, Washington D.C.), Technical report (2020).
25. E Strategies, Western Flexibility Assessment: Investigating the West's Changing Resource Mix and Implications forSystem Flexibility, (Western Interstate Energy Board, Salt Lake City, UT), Technical report (2019).
26. CAISO, 2020-2021 Transmission Plan, (California ISO), Technical report (2021).
27. APPENDIX H: Pacific Northwest – California Transfer Increase Informational Special Study, Technical report (2019).
28. HIFLD, Electric Power Transmission Lines (2020).
29. E3, Generation and Transmission Resource Cost Update 2019 (2019).
30. Multi-Resolution Land Characteristics (MRLC) Consortium, Land Cover Data (2016).
31. aLGAP USGS Core Science Analytics, Synthesis, USGS Gap Analysis Project - Additional Data – Slope (2011).
32. GC Wu, et al., Power of Place: Land Conservation and Clean Energy Pathways for California (Executive Summary), (The Nature Conservancy, San Francisco, CA), Executive Summaryu (2019).
33. D of Transportation, Core Based Statistical Areas (National) (2020).
34. JH Scott, et al., Wildfire Risk to Communities: Spatial datasets of landscape-wide wildfire risk components for the United States (2020).
35. SWCA, Environmental Mitigation Costs Study - Final Report for the Western Electricity Coordination Council, (Western Electricity Coordination Council (WECC)), Technical report (2013).
36. C ISO, Participating transmission owner per unit costs (2020).
37. KL Hall, Out of Sight, Out of Mind 2012 - An Updated Study on the Undergrounding Of Overhead Power Lines, (Edison Electric Institute), Technical report (2013).

38. ESRI, Cost Path As Polyline (year?).
39. P Beiter, et al., The Cost of Floating Offshore Wind Energy in California Between 2019 and 2032, (National Renewable Energy Lab, Golden, CO), Technical Report NREL/TP-5000-77384 (2020).
40. NYD of Public Service Staff, New York State Energy Research and Development Authority Staff, TB Group, P Consulting, Initial Report on the New York Power Grid Study, (New York State Public Service Commission), Technical report (2021).
41. NREL (National Renewable Energy Laboratory), 2020 Annual Technology Baseline, (Golden, CO: National Renewable Energy Laboratory), Technical report (2020).
42. JH Williams, et al., Carbon-Neutral Pathways for the United States. *AGU Adv.* **2**, e2020AV000284 (2021) 00007 _eprint: https://agupubs.onlinelibrary.wiley.com/doi/pdf/10.1029/2020AV000284.
43. BM Sleeter, et al., An integrated approach to modeling changes in land use, land cover, and disturbance and their impact on ecosystem carbon dynamics: a case study in the Sierra Nevada Mountains of California. *AIMS Environ. Sci.* **2**, 577–606 (2015) 00000.
44. S Ong, C Campbell, P Denholm, R Margolis, G Heath, Land-Use Requirements for Solar Power Plants in the United States, (National Renewable Energy Laboratory, Golden, CO), Technical Report NREL/TP-6A20-56290 (2013).
45. RR Hernandez, MK Hoffacker, ML Murphy-Mariscal, GC Wu, MF Allen, Solar energy development impacts on land cover change and protected areas. *Proc. Natl. Acad. Sci.* **112**, 13579–13584 (2015) 00053.
46. WECC, Black & Veatch Corp., Energy and Environmental Economics, WECC 2019 Transmission Capital Cost Tool - with E3 Updates (2019) 00000.
47. SE Baker, et al., Getting to Neutral: Options for negative carbon emissions in California, (Lawrence Livermore National Laboratory), Technical Report LLNL-TR-796100 (2020).
48. WL Rooney, J Blumenthal, B Bean, JE Mullet, Designing sorghum as a dedicated bioenergy feedstock. *Biofuels, Bioprod. Biorefining* **1**, 147–157 (2007) 00703 _eprint: https://onlinelibrary.wiley.com/doi/pdf/10.1002/bbb.15.
49. ME Salassi, et al., Farm-Scale Cost of Producing Perennial Energy Cane as a Biofuel Feedstock. *BioEnergy Res.* **7**, 609–619 (2014) 00019.
50. KB Hall, et al., A Growth and Yield Model for Eucalyptus benthamii in the Southeastern United States. *For. Sci.* **66**, 25–37 (2020) 00003.
51. RT Belote, et al., Identifying Corridors among Large Protected Areas in the United States. *PLOS ONE* **11**, e0154223 (2016) 00029.
52. MC Reeves, JE Mitchell, Extent of Coterminous US Rangelands: Quantifying Implications of Differing Agency Perspectives. *Rangel. Ecol. & Management* **64**, 585–597 (2011) 00014.
53. CDC/ATSDR, 2018 Social Vulnerability Index (SVI) (2018).
54. AN Rose, JJ McKee, ML Urban, EA Bright, KM Sims, LandScan 2018 (2019) Edition: 2018 Place: Oak Ridge, TN Section: July 1, 2019.
55. RG Sullivan, et al., Wind Turbine Visibility and Visual Impact Threshold Distances in Western Landscapes, (Argonne National Laboratory), Technical report (year?).
56. RG Sullivan, JM Abplanalp, Utility-scale solar energy facility visual impact characterization and mitigation study, (Argonne National Laboratory), Technical report (2013).
57. RG Sullivan, et al., Visual Impacts of Utility-scale Solar Energy Facilities on Southwestern Desert Landscapes, (Argonne National Laboratory), Technical report (year?).
58. RG Sullivan, et al., Electric transmission visibility and visual contrast threshold distances in western landscapes, (Argonne National Laboratory), Technical report (year?).
59. B Cohen, C Shank, California Industrial-Size Solar Energy Generation Arrays (2018) 00000.
60. Bureau of Land Management, BLM Western Solar Energy Program (WSEP) (2012) 00000.
61. Western Electricity Coordinating Council, ICF, WECC Environmental Data Viewer and Risk Mapping (year?) 00000.
